# Political and affective polarisation in a democracy in crisis: The E-Dem panel survey dataset (Spain, 2018–2019)

**DOI:** 10.1016/j.dib.2020.106059

**Published:** 2020-07-23

**Authors:** Mariano Torcal, Andrés Santana, Emily Carty, Josep Maria Comellas

**Affiliations:** a Universitat Pompeu Fabra, Plaça de la Mercè, 08002, Barcelona, Spain; b Universidad Autónoma de Madrid, 28049, Madrid, Spain; c Universidad de Salamanca, 37008, Salamanca, Spain; d Universitat Pompeu Fabra, Plaça de la Mercè, 08002, Barcelona, Spain

**Keywords:** Dataset, Affective polarisation, Political polarisation, Elections, Voting behaviour, Challenger parties, Crisis, Spain

## Abstract

The E-DEM dataset provides information on the evolution of political and affective polarisation and electoral behaviour in the aftermath of the political crisis that shook the Spanish party system starting in 2014. The dataset is formed by a four-wave online panel survey of the Spanish voting age population between late October 2018 and May 2019. The four waves coincide with key moments in Spanish political life including local, regional, national, and European elections, as well as the conviction of Catalan secessionist leaders. It also covers the six-month period of the surge of Spain's new radical right party, Vox, spanning from shortly before its first major electoral success in Spain's most populous region, Andalusia, to its consolidation in the May 2019 European elections. The sample, which reflects the general population in terms of age, gender, and geographical province, consists of 1,484 panellists who completed the four waves, while the samples for individual waves are larger, ranging from 1,659 to 2,501 respondents. The data is especially useful for researchers who wish to explore dynamics of ideological and affective polarisation, factors that explain the rise of new parties, and for those investigating the evolution of political attitudes in general.

**Specifications Table**

**Table:** 

**Subject**	Sociology and Political Science
**Specific subject area**	Political and affective polarisation; Electoral behaviour; Public opinion; Mass and social media consumption, Survey experiments
**Type of data**	Table, Matrix
**How data were acquired**	Four-wave online panel survey of a sample of the voting-age population in Spain. Four different original survey experiments were embedded into the survey that taps into political trust as well as exposure to news and social media. Individuals were randomly selected to form the different treatment groups. The recruitment of respondents from an online panel and the data collection process was administered by Netquest.
**Data format**	Raw data in .dta format (STATA 15).
**Parameters for data collection**	Spanish voting-age (18 years or older) population at the time of data collection. To accurately reflect characteristics of the national general population, the sample was selected with the application of the following quotas: gender, age, and geographical province. Wave 1: 2,501 interviews, 25 October 7 November 2018. Wave 2: 1,890 interviews, 12-19 February 2019. Wave 3: 1,659 interviews, 23-26 April 2019. Wave 4: 2,059 interviews, 17-25 May 2019.
**Description of data collection**	Data were collected in a panel survey, the four waves of which were implemented before the Andalusian regional elections (held on December 2, 2018), after those elections, before the Spanish General elections (held on April 28, 2019) and before the concurrent European Parliament and Spanish local and regional elections (all held on April 26, 2019). Sample recruitment and the data collection process were carried out by Netquest. The overall participation rate among those who were invited was 91.6%, and the overall completion rate among those who participated was 75.9%.
**Data source location**	Institution: Universitat Pompeu Fabra City/Town/Region: Barcelona, Catalonia Country: Spain
**Data accessibility**	Repository name: Mendeley Data Data identification number: http://dx.doi.org/10.17632/6bt6r8cn2r.3 The data protocol & codebook, as well as the questionnaires for each of the four waves, are provided as supplementary files, and are also available at the Mendely Data site.

## Value of the Data

• This dataset consists of a four-wave micro-panel that were implemented during a period of important political crisis and change in Spain. Because of this, the data are useful to assess the degree of contextual changes on public opinion, allowing us also to estimate individual-level variation (within variation) as well as across-individual differences (between variation).
• To the best of our knowledge, the data contain the most exhaustive list of indicators of affective and ideological polarisation in Europe to the date, allowing for the construction of many individual-level indicators of polarisation and for the exploration of their “between” and “within” variation. For established scholars, practitioners, and graduate students, this is a perfect instrument with which to study this topic.
• Those researching electoral behaviour can benefit from these data to understand the micro-foundations and contextual factors that could alter affective political polarisation in today's democracies, especially in regards to the influence of media consumption, and Internet and social network activity.
• There are four survey experiments embedded in the four waves that provide additional information on the causality behind affective polarisation, media effects, and social media exposure.
• These data can be used to gain further insight into the effects of polarisation on electoral behaviour. They also permit a more detailed look into the interactions between party supply, social media exposure and polarisation.
• These data are also helpful for individuals interested in the study of the sudden rise of a new radical right party as the associated consequences.

## 1 Data description

The E-DEM dataset is a micro-level online panel survey of the Spanish voting age population comprised of four waves carried out over a six-month period between late October 2018 and May 2019. It is available at the Mendely Data site (http://dx.doi.org/10.17632/6bt6r8cn2r.3). The E-DEM project comprises as well four experiments, respectively embedded in the first, second, third and fourth waves of the online panel survey. The experimental dataset is available in the same Mendeley Data site, which also offers and integrated dataset merging the online panel survey and the experimental datasets. The three datasets (online panel survey, experimental, integrated) are available in three alternative formats: tab-separated delimited text, tab-separated rawtext, and Stata 15.0 (.dta).

**Fig. 1:**
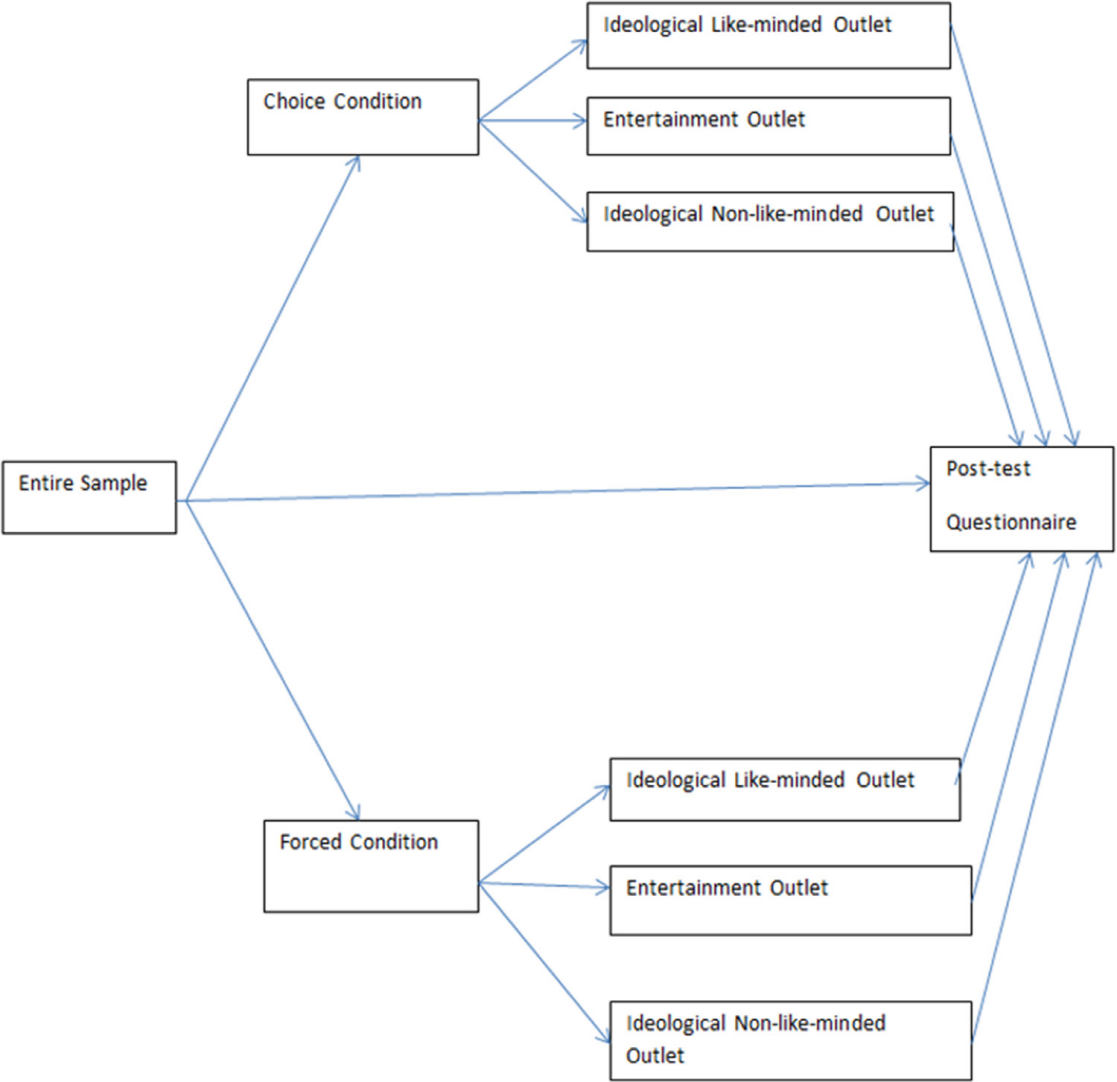
Basic graphical representation of the experimental (*post*-*test*) design. *Source*: own elaboration.

The data protocol & codebook, as well as the questionnaires for each of the four waves, are also available at the Mendely Data site. They are moreover provided as supplementary files of this *Data in Brief* article, which also includes five tables and a figure: a specifications’ table; Table 1, with a list of the main variables in the dataset; Table 2, on the timing of the waves and related major political events in Spain; Table 3, with the structure of the data (invitations, participation, and discarded interviews); Table 4, with the details on attrition; and Fig. 1, displaying the research design of the second experiment.

**Table 1:** List of main variables organized by topics.

*Time-variant variables (all waves):*
Indicators of affective polarisation: a) Feelings towards and trust in supporters of different parties b) Feelings towards different political parties c) Feelings towards leaders of different parties d) Feelings towards and trust in different subnational and regional identity groups e) Feelings towards and trust in different social, religious, or economic groups Left-right scale (self and party placements) Trust in (with and without experiments): a) The Spanish Parliament b) The Spanish government c) Regional parliaments d) Regional governments e) Spanish politicians and political parties f) The Spanish police g) Spanish courts h) The European Parliament i) The European Commission Three indicators of social trust Political interest Satisfaction with the functioning of democracy Satisfaction with the current economic situation Opinions on issues and current problems: a) Three most important problems in the country b) Current evaluation of main issues in the country c) Individual position on a set of relevant issues Media use and offline/online political participation: a) Frequency of exposure to offline media in general b) Frequency of exposure to online media in general c) Use of different social media d) Use of media and social media to obtain political information e) List of political activities done on the Internet and social media f) Agreement/disagreement with opinions on social media and other media sources Party identification and expressive partisanship Personal economic condition index (ISSP) Evaluation of income situation for family household Occupation
*Time-invariant or almost invariant variables (1st wave only):*
Frequency of, and agreement with others in political discussions offline Internal and external efficacy Membership and organizational activity (wave 2) Attachment to/identification with different territories/communities:
a) City or town b) Region c) Spain d) Europe Demographic and other social characteristics Socio-economic status Political knowledge I (civic knowledge) Political knowledge II (topical questions) Vote recall in the last national elections waves 1 and 4)
Time-variant variables (rotated in 1st, 2nd, 3rd, and 4th waves):
Frequency and agreement with others in political discussion taking place offline and online (wave 2) Interest in the electoral campaign (waves 3 and 4) Interest in the coverage of electoral campaigns by different mass media (waves 3 and 4) Interest in the coverage of electoral campaigns by different social media (waves 3 and 4) Evaluation of the economy (retrospective and prospective) (waves 2, 3, and 4). Satisfaction with the political situation (waves 3 and 4). Satisfaction with the job performance of the national government and the opposition (waves 2, 3, and 4) Evaluation of the main national and regional party leaders (waves 2, 3, and 4) Modes of political participation online and offline (waves 1 and 2) Attitudes towards the European Union (EU) (wave 4): a) Pro/anti EU scale (self and party placements) b) Opinions on European integration c) Satisfaction with democracy in the EU Self-reported probability to vote in the general and EU elections (waves 3 and 4). Self-reported probability of vote for different parties (waves 2 and 4) Leaders’ evaluation traits (waves 3 and 4) Negative partisanship (waves 3 and 4) Vote intention (waves 2, 3, and 4)

**Table 2:** Timing of the waves and related major political events in Spain.

Wave	Begin	End	Days	Gap	Major political events in Spain
Wave 1	25/10/2018	07/11/2018	14	n.a.	Andalusian regional elections (2/12/2018)
Wave 2	12/02/2019	19/02/2019	8	97	Formation of the Andalusian regional government (16/01/2019)
Wave 3	23/04/2019	26/04/2019	4	63	Spanish general elections (28/04/2019)
Wave 4	17/05/2019	24/05/2019	8	21	Spanish local, regional, and European elections (26/05/2019)
All	25/10/2018	24/05/2019	34	181	

*Notes*: Days = The number of days during which survey responses were collected. Gap = time elapsed, in days, from the last day of data collection of the previous wave to the first day of response collection of the current wave; n.a.: not applicable, since in the first wave there is no previous wave with respect to which a time gap may be calculated.

**Table 3:** Invitations, participation, and data cleaning in the four waves.

Wave	Wave 1	Wave 2	Wave 3	Wave 4	Sum
Rejected and accepted invitations					
Invited	4762	2506	1892	2506	11,666
Rejected	589	85	127	181	982
Accepted	4173	2421	1765	2325	10,684
Participation rate	87.6%	96.6%	93.3%	92.8%	91.6%
Discarded and completed interviews					
Accepted	4173	2421	1765	2325	10,684
Discarded	1672	531	106	266	2575
Declined	0	19	15	26	60
ISO unmet	37	35	21	26	119
Incomplete	259	77	62	116	514
Invalid	0	390	1	45	436
Closed	1130	10	7	4	1151
Quota full	246	0	0	49	295
Completed	2501	1890	1659	2059	8109
Completion rate	59.9%	78.1%	94.0%	88.6%	75.9%

*Notes*: Invited = invited to do the survey or redirected to it from another survey (only 76 participants were redirected, all of them in the first wave). Accepted = accepted the invitation and entered the application to see the survey description. Participation rate = the proportion of those that accepted after they were invited. Declined = entered the application and, when seeing the description of the study and the associated research team, preferred not to do the survey. ISO unmet = failed to meet at least one of the three ISO criteria. Incomplete: started, but did not finish the interview. Invalid: responses were invalidated because the application did not save the answers of some questions. Closed = accessed or completed the survey when the data collection window had already been closed. Completed = accepted – (declined + ISO unmet + incomplete + invalid + closed + quota full). Completion rate = the proportion of those individuals who successfully completed the survey after accepting the invitation.

**Table 4:** Wave attrition.

Wave	Wave 1	Wave 2	Wave 3	Wave 4
Completed	2501	1890	1659	2059
Consecutive completion	n.a.	1890	1659	1484
Immediate permanence rate	n.a.	75.6%	87.8%	89.5%
Cumulative completion	2501	1890	1659	1484
Cumulative permanence rate	100.0%	75.6%	66.3%	59.3%

*Notes*: Completed = accepted – (declined + ISO unmet + incomplete + invalid + closed + quota full). Consecutive completion = completed the current and the immediately previous wave. Immediate permanence rate = consecutive completion / completed. Cumulative completion = completed the current wave and all the previous ones. Cumulative permanence rate = cumulative completion / completed in wave 1. n.a.: not applicable.

Table 1 shows a list of the main variables organized by topics. The surveys include questions on socio-demographic characteristics, self-reported voting behaviour and intentions, non-electoral political participation, membership and involvement in a broad array of social and political associations and organisations, an equally ample catalogue on media consumption and internet and social networks usage, an especially rich battery of measures of political and affective polarisation, a wealth of variables tapping into political opinions, attitudes, and orientations, a series of indicators on trust in political parties and institutions, as well as in people of several relevant social groups, and a profuse set of evaluations of the economic and political situation. Most of these questions are asked in all the waves, enabling both the study of their evolution and the assessment of the impact of their changes at the individual level on other variables. The battery of political and affective polarisation indicators is especially rich in that it considers attitudes towards candidates, parties, and individuals belonging to relevant social and political groups, as is the battery on political opinions which inquires about the placement of the most important Spanish political parties on a wide array of dimensions.

Of note is that the socio-demographic characteristics of the participants is remarkably stable along the surveys and very close to official statistical records of the Spanish Institute of Statistics, with a slight underrepresentation of women and of respondents of the youngest and the oldest age groups.

## 2 Survey and experimental design, sampling and other methodological issues

### 2.1 The survey panel design and the timing of the waves

The data are comprised of a four-wave online panel survey of the Spanish voting age population conducted between October 2018 and May 2019. Table 2 displays the timing of the waves, all of which took place in the short time span of half a year. This period of time covers four key political moments in the recent evolution of Spanish politics and party system. The first wave was carried out in late October and early November 2018, before the Andalusian regional elections in which the new radical right party VOX achieved its first major electoral success, thereby securing its entrance to Spain's most populated region. Wave 2 took place in February 2019, shortly after the newly-elected Andalusian regional government formed and took office with VOX as a key member of the governing coalition. Wave 3 was administered in late April 2019, the week before the Spanish general elections of April 2019. Finally, the data for wave 4 were collected in late May 2019, the week before the concurrent local, regional, and European elections in Spain.

### 2.2 The survey administration and data collection

The survey was administered by Netquest using their large online non-probabilistic panel. Netquest (https://www.netquest.com/es/home/encuestas-online-investigacion) is an online people-based data collection company with 17 years of experience. Founded in Barcelona, Netquest currently conducts public opinion studies in 27 countries in Europe and the Americas. To do so, the company relies on online opt-in panels of people that are willing to participate in surveys and to share data about their online activity. Netquest currently works with various market research companies, public institutions and universities worldwide.

### 2.3 The sample weights and data cleaning process

Sample weights were applied to ensure that the sample aligns with charateristics of the general population in terms of region of residency, gender, and age. For each wave, Table 3 shows the number of invited participants, those who accepted the invitations, and those who failed to complete the questionnaire due to various reasons. The overall acceptance rate to participate was close to 91.6% and the overall completion rate reached 75.9%. In accordance with Netquest's standard procedures, the original data retrieved from the participants were cleaned to conform to ISO procedures. In particular, some interviews were discarded either because the socio-demographic profiles did not match those in the database in terms of gender or age, because the time a respondent took to complete the whole survey was at least 20 percent lower than its estimated duration, or because individuals failed to pass an attention check or ‘trick’ question aimed at confirming that the participant was paying attention. The combined number of interviews dropped due to any of these ISO criteria being unmet was remarkably low, ranging from 21 in wave 3 to 37 in wave 1. A somewhat larger number of interviews was discarded because they were incomplete, i.e., they had been started, but not finished, or were invalidated because the program did not save the answers to some questions. Finally, respondents were also removed because they had been completed after the data collection window had closed (this was only relevant in wave 1); or because the quota for a respondent's profile had already been filled (again, this was only consequential in wave 1). After taking into account all of these circumstances, the effective number of completed surveys oscillated between slightly over 1650 in wave 3 and 2500 in wave 1.

### 2.4 Wave attrition

To illustrate the rates of attrition across waves, Table 4 displays of the number of respondents who completed questionnaires in each wave (row 1) and each pair of consecutive waves (row 2), the immediate rate of permanence from one wave to the next (row 3), the number of respondents who completed a wave's questionnaire and all the former ones (row 4), and the corresponding cumulative rate of permanence (row 5). The second wave is nested in the first one, in that respondents of the second wave are a subsample of the first one: hence, the second wave's figures of row 1, row 2 and row 4 are identical (n_2_ = 1890). Likewise, the third wave is nested in the second wave and therefore also on the first one (n_3_ = 1659). To allow for a larger number of responses in the fourth wave, all the panellists who had completed the first wave were invited to participate in the fourth one, even if they had not completed the two previous ones; in practical terms, this means that the fourth wave is nested in the first but not in the second and third waves; and that the fourth-wave's quantity of completed interviews (n_4_ = 2059) outnumbers the amount of consecutive and cumulative interviews (*m* = 1484). The immediate rates of permanence in row 3 capture the proportion of panellists in each wave who completed the survey in the next one. These rates are considerably high, ranging from three out of four cases in the second wave to nine out of ten in the fourth one. For instance, the immediate rate of permanence of the fourth wave indicates that 89.5 per-cent of those who completed the third wave also completed the fourth. The cumulative rates of permanence in row 5 capture the percentage of first-wave panellists who completed each wave; hence, the higher they are, the lower the attrition in the panel, which is one of the main concerns with micro-panel survey data. The figure for the cumulative rate of permanence of the fourth wave indicates that 59.3% of those who completed the first wave also completed the second, third and four waves.

### 2.5 Basic strategy for DK/NA

In the design of this panel survey we paid special attention to uncertain responses (i.e. “don't know”, “no option” or “decline to answer”). Following the recommendations for web questionnaires provided by Couper [1] and Callegaro [2], in our design we attempted to reduce item nonresponse without contributing to other sources of error.

As has been shown in previous studies, providing explicit options for uncertain responses seems to increase the proportion of respondents who select them, as it will not only give an option to people who do not know what to answer, but also to those who aim to minimize their effort to answer the questionnaire. However, forcing a respondent to answer every question does not seem to be the most appropriate option either. On the one hand, this is likely to diminish the quality of the data as a result of increasing respondent frustration at being required to provide an answer which may not be in line with her/his actual opinion. More importantly, as Couper [1] and Dillman [3] show, forcing respondents to select a response (or a limited range of responses) raises ethical concerns, violating basic norms of voluntary participation.

Following this preceding discussion, we decided not to provide explicit uncertain response options, while giving the chance for skipping a question. However, skipping the question is not a comfortable option either, especially for interactive self-administered surveys [1]. With this type of survey, we can remind the respondent the importance of her/his response and request that they confirm if she/he wants to keep advancing the questionnaire anyway. As Derouvray and Couper explain [4], this strategy does not produce break-offs or abandonments and at the same time it does not force a non-desired response. Yet, if a “don't know” option is necessary, as in the case of knowledge questions in which this is in itself a possible answer, then we combined it with another friendly probe, similar to the pop-up reminder. In brief, in our web questionnaire for the 4 waves:

1. We refrained from explicitly providing “I prefer not answer” responses.
2. We limited the use of “don't know” options to questions that require some kind of knowledge, such as locating all the political parties in a scale (for instance, left-right).
3. We allowed respondents to not answer a question by skipping it and proceeding to the next one, although they had to confirm their choice by responding to a pop-up alert.
4. Respondents received a similar alert in knowledge questions if they answered “don't know”.

### 2.6 The experiments embedded in the different waves

Four different survey experiments were embedded in different waves of the surveys:

a) Wave 1: An experiment to measure political trust.
b) Wave 2: An experiment on “Ideological preferences, selective exposure to media political information, framing and affective polarisation”.
c) Wave 3: An experiment on “Ideological preferences, selective exposure to social media and affective polarisation in national elections”.
d) Wave 4: An experiment on “Ideological preferences, selective exposure to social media and affective polarisation in European elections”.

a) *Wave 1: Experiment to measure political trust:*

The problem of the dimensionality of political trust is not only theoretical but also a methodological one. Aside from the standard method to measure political trust used by the American National Election Study (ANES), criticized on account of its vagueness and the imprecision of its object, there are two basic methods for measuring political trust. One is the common indicator used by the General Social Survey (GSS), Gallup, and the World and European Values Surveys (WVS/EVS), which ask people how much confidence they have in “the people running the institutions in the country”. These measures have the virtue of being flexible enough to be applied to different institutions, but they still present some problems. First, these measures use the concept of confidence instead of trust, suggesting both concepts are interchangeable. Secondly, it contains an unclear reference to the object of trust (the institution vis-a-vis the people temporarily running them).

More recently, the Eurobarometer (employing binary indicators) and the ESS (using a 0–10 scale) have adopted a battery of questions tapping trust in a set of institutions, which is becoming the most accepted way to measure political trust. However, the use of a sequential grid of institutions might also present a problem. As it has been proven by the literature on survey methodology [5,6], correlations (satisficing) amongst these items tend to decrease substantially when items are separated. An alternative is to present this battery of institutions in different settings.

Additionally, there are promising alternative approaches to conceptualize political trust and mistrust in terms of perceived institutional trustworthiness, which requires identifying the domains upon which this trustworthiness is based. As Carlin [7] argues, survey research in political trust should adopt these approaches by extending the response scale of items to tap distrust in more institutions and by tackling the differential weighting inherent to the criteria in each domain.

In an experiment embedded in wave 1, we used an algorithm to create six different random subsamples and assigned them six different treatments to address the dimensionality and measurement problem of political trust:

1. Subsample 1: We used the complete battery of institutions in a grid. The order of the institutions in the grid was rotated randomly. This group (or sample) constitutes the baseline.
2. Subsample 2: We used of the same list of institutions, but they were presented sequentially instead of showing the whole grid. The order of the institutions was rotated randomly.
3. Subsample 3: We used different batteries in separate grids but organized the grids according to potential dimensions of political trust at the national, subnational and EU levels of government. The order of the institutions in each grid was rotated randomly.
4. Subsample 4. We exposed this group to a text extracted from a real report about the degree of turnover of representatives in the national parliament in Spain and how this renovation has resulted in one of the youngest parliaments in the EU. We used the complete grid of institutions, as in sample 1.
5. Subsample 5. We exposed this group to a text extracted from a real report about the level of education and good technical training of the political class in Spain at all levels of government. Again, we used the complete grid of institutions.
6. Subsample 6: We exposed this group to a text extracted from a real report about the efficiency of courts and degree of accessibility to them for citizens in Spain compared with many other EU countries. We used the complete grid of institutions, as in sample 1.

b) Wave 2: An experiment on Ideological preferences, selective exposure to media political information, framing and affective polarisation

The evidence of the effects of media content on political polarisation and political trust is inconclusive. According to Brosius, van Elsas and de Vreese [8], a possible explanation for these mixed findings is that most studies rely on self-reported media use which may not be accurate representations of actual media consumption. Additionally, most studies do not take the content of the media to which respondents have been exposed into account. As reported by Geiβ and Schäfer [9], media visibility (the sheer amount of coverage of a topic) (agenda setting) and media tone (the evaluation of a topic) (priming) can have complementary effects on political attitudes and behaviour. Additionally, as Gaines and collaborators noted [10], the preceding arguments are based on the assumption that citizens are attentive to and capable of (or willing to) capture the overall sentiment of the content they read or receive from the media. However, as Gunther and collaborators [11] remarked, it could be the case that media content interacts with the source brand, overriding the content effect. Thus, according to Prior [12], the study of the distinctive effects of news brands vis-a-vis news contents on political polarisation is important, because both might not always coincide.

To improve our understanding and better address these problems, we designed an experiment building on and trying to extend existing research on media exposure, partisan news and attitude polarisation [13], [14], [15], [16], [17]. To do this, we followed the more recent innovative designs proposed by Gaines and Kuklinski [18], Arceneaux and Johnson [19] and Feldman et al. [20], often referred to as “preference trial” or “participant preference”.

The basic structure of this post-test design is presented in Fig. 1. As the first step, participants are randomly exposed to the experimental stimulus, which consists of being assigned to either a “forced” condition or a “choice” condition, leaving a third group as a control group without any exposure to news media. Random assignment was produced by a computer algorithm. In the “forced” condition, a second algorithm assigns subjects randomly to one of these options: a) a like-minded ideological outlet; b) a non-like-minded ideological outlet, or c) an entertainment outlet. In the “choice” condition, respondents are instead asked to choose amongst the same list of outlets. In each of these outlets, the stimulus is a written article on immigration although the content (overall sentiment) of the articles does not always correspond with the general ideological line of each outlet. This design allows distinguishing content news effect vis-à-vis outlet branding.

In the final step, subjects completed a post-experiment questionnaire to provide us with indicators of their affective polarisation towards immigrants (thermometer of feelings and trust levels). We added two post treatment questions about the stimulus: topic of the news they read and the general overall sentiment they think the news conveyed. In addition, the dataset contains a variable reflecting the individual time each respondent spent in reading the news (this variable was automatically generated by the system).

The randomization procedure was managed by an algorithm. Information about pre-existing ideological preferences was provided by co-variate information obtained before the administration of the experiment.

c) Wave 3: An experiment on “Ideological preferences, selective exposure to social media and affective polarisation in national elections”

The purpose of this experiment was to test the effect of exposure to six national leaders’ Twitter accounts on affective polarisation. Participation in the experiment was restricted to invitation and to those who had existing Twitter accounts (consent in both cases was required by law). In this case, there was no randomization procedure and the invitation was valid for three days. Because of this, we could only test the self-selection exposure to these accounts on affective polarisation. The experiment concluded with questions about the participants’ exposure to and the content of the selected Twitter accounts. To verify how much time respondents spent looking at those Twitter accounts, we collected information on their activity with a passive behavioural metre.

d) Wave 4: An experiment on “Ideological preferences, selective exposure to social media and affective polarisation in European elections”

In the fourth wave, we repeated the preceding experiment (the social media and affective polarisation experiment embedded in Wave 3) in the context of the elections to the European Parliament. Again, participation was restricted via invitation and only to those who had pre-existing Twitter accounts. In this experiment, however, we created an additional experimental group that was asked to choose amongst a list of five EU institutional Twitter accounts we selected. Assignment to the first list, containing the most significant national politicians’ accounts, or to the second one, with the above-mentioned institutional Twitter accounts, was randomized by a computer algorithm. As with the previous experiment, to verify how much time respondents spent looking into those Twitter accounts, we collected information with a passive behavioural metre.

## Declaration of Competing Interest

The authors declare that they have no known competing financial interests or personal relationships which have, or could be perceived to have, influenced the work reported in this article.

## Appendix Supplementary materials

Image, application 1

Image, application 2

Image, application 3

Image, application 4

Image, application 5
