# Supplementary material for: Political and affective polarisation in a democracy in crisis: The E-Dem panel survey dataset (Spain, 2018–2019)
Source: Data Brief. 2020 Jul 23;32:106059. doi: 10.1016/j.dib.2020.106059 (PMC7451797; doi:10.1016/j.dib.2020.106059)
Supplement: Supplementary file 1 [file mmc1.pdf]

Online Political Participation and Deliberation in a Democracy in Crisis

A New Methodological Approach (E-Dem) (2017-2020)

Data protocol

Funding

This research was supported by competitive grant funding from the Spanish Ministry of Economy and Competitivity. Ministerio de Economía y Competitividad, Programa Estatal de Fomento de la Investigación Científica y Técnica de Excelencia (CSO2016-79772-P, 2017-2019, Principal Investigador: Mariano Torcal). The views expressed herein are those of the authors and are not necessarily those of the Spanish Ministry of Economy and Competitiveness, State Program for the Promotion of Scientific and Technical Research of Excellence. The PI of the project is also grateful for the funding provided by the Advance Research Fellowship Programme ICREA, funded by the Catalonian Government.

Index

Index of Tables **5**

Technical Information **7**

1. Citation, Research Team and Contact **7**

Citation 7

Research Team 7

Contact 7

2. Data Description **9**

Overview 9

Files 9

3. General Sample Design of the Survey **11**

Field 11

Universe 11

Sample size 11

Fieldwork 11

Sampling Method 11

Fieldwork Information 12

4. Structure of the Sample **13**

Distribution of Shares 13

Attrition 15

Quota Distribution 16

5. Coding, Naming, and Labelling Protocols **19**

Coding of Missing, Non-Response and Non-Applicable values 19

Protocol for Naming Variables 20

Protocol for Labelling Variables 22

Protocol for Labelling Variable Values 23

Naming and Labelling Language 24

Survey variables **25**

6. Variable List **25**

Global Variables 26

Wave-Specific Variables 27

Socio-Demographic Variables 28

Opinion, Attitudinal and Beliefs Variables 30

Trust in Political Institutions Non-Experimental Variables 48

First Experiment Variables (Trust in Political Institutions) 49

Second Experiment Variables (Traditional Media and Framing) 52

Third Experiment Variables (Social Media in National Elections) 53

Fourth Experiment Variables (Social Media in European Elections) 55

7. Codes for Categorical Variables 57
Global and Wave-Specific Categorical Variables 57
Socio-Demographic Categorical Variables 57
Opinion or Attitudinal Categorical Variables 62
Experimental Categorical Variables 72

Index of Tables

Table 1 Timing of the Waves 12
Table 2 Structure of the Sample 13
Table 3 Wave Attrition 15
Table 4 Socio-Demographic Characteristics of the Participants, by Wave 16
Table 5 Examples of Variable Names (Non-Experimental Variables) 21
Table 6 Examples of Variable Names (Experimental Variables) 22
Table 7 List of Global Variables 26
Table 8 List of Wave-Specific Variables 27
Table 9 List of Socio-Demographic Variables 28
Table 10 List of Opinion and other “p” Variables 30
Table 11 List of Trust in Political Institutions Non-Experimental Variables 48
Table 12 List of Variables for the First Experiment (Trust in Political Institutions) 49
Table 13 List of Variables for the Second Experiment (Traditional Media and Framing) 52
Table 14 List of Variables for the Third Experiment (Social Media during National Elections) ... 53
Table 15 List of Variables for the Fourth Experiment (Social Media in European elections) 55

E-Dem 2017-2020 Dataset

Technical Information

1. Citation, Research Team and Contact

Citation

This dataset is provided free of charge for all those who wish to use it. Designing this study, retrieving the data, cleaning it, and preparing it for public use meant a lot of work. We are therefore grateful for your acknowledgment of our efforts by citing the database when you use it. The suggested citation is the following:

Torcal, Mariano, Andrés Santana, Emily Carty and Josep Maria Comellas (2020) Political and affective polarisation in a democracy in crisis: The E-Dem panel survey dataset (Spain, 2018-2019), *Data in Brief*, forthcoming.

Research Team

Mariano Torcal (Universitat Pompeu Fabra).
Andrés Santana (Universidad Autónoma de Madrid).
Javier Lorenzo (Universidad Carlos III de Madrid),
Emily Carty (Universidad de Salamanca),
Melanie Revilla (Universitat Pompeu Fabra),
Pedro Riera (Universidad Carlos III de Madrid),
Josep Maria Comellas (Universitat Pompeu Fabra),
Daniela Tamayo (Universidad Autónoma de Madrid),
Sergio Martini (Università degli Studi di Siena),

Contact

Mariano Torcal
Mail: mariano.torcal@upf.edu

Additionally, you can contact:
Andrés Santana
Mail: andres.santana@uam.es
Twitter: https://twitter.com/SantanaAndres3

2. Data Description

Overview

The E-DEM dataset is a micro-level online panel survey of the Spanish voting age population comprised of four waves carried out over a six-month period between late October 2018 and May 2019 (the detailed timing of each wave will be presented in Table 1). The survey waves coincide with key moments in Spanish political life (including local, regional, national, and European elections, as well as the conviction of Catalan secessionist leaders). It also covers the six-month period of the surge of Spain’s new radical right party, Vox, spanning from shortly before its first major electoral success in Spain’s most populate region, Andalusia, to its consolidation in the May 2019 European elections. In addition, the project comprises a series of survey experiments, embedded in the different waves, regarding measures on confidence in institutions, exposure to media and social networks, as well as on political behaviour and polarization attitudes based on passive data, captured with software that the interviewees installed on their mobile devices.

The following protocol contains technical information concerning the online panel survey methodological approach.

Files

Data protocol and codebook (.pdf file)

E-DEM integrated four-waves panel and experimental data (Stata 15.0 .dta file)

E-DEM integrated four-waves panel and experimental data (tab-separated delimited text)

E-DEM integrated four-waves panel and experimental data (tab-separated rawtext)

E-DEM four-waves panel survey (Stata 15.0 .dta file)

E-DEM four-waves panel survey (tab-separated delimited text)

E-DEM four-waves panel survey (tab-separated rawtext)

E-DEM experimental database (Stata 15.0 .dta file)

E-DEM experimental database (tab-separated delimited text)

E-DEM experimental database (tab-separated rawtext)

E-DEM Behavioural data collected with Passive Meter (forthcoming)

3. General Sample Design of the Survey

Field

National (Spain).

Universe

General population of more than 18 years, with the software to capture behaviour in internet installed, after consent, on one of its electronic devices.

Sample size

8,109 interviews completed.

Fieldwork

Administrated by Netquest, a non-probabilistic panel with more than 70,000 panellists in Spain. It currently conducts public opinion studies in other 27 countries in Europe and the Americas.

Sampling Method

Non-probability quota sampling.

Fieldwork Information

Performed between 25/10/2018 and 24/05/2019. Table 1 details the exact fieldwork period of each wave.

Table 1 Timing of the Waves

Wave	Begin	End	Days	Gap
Wave 1	25/10/2018	07/11/2018	14	n.a.
Wave 2	12/02/2019	19/02/2019	8	97
Wave 3	23/04/2019	26/04/2019	4	63
Wave 4	17/05/2019	24/05/2019	8	21
ALL WAVES	25/10/2018	24/05/2019	34	181

Source: own elaboration.

Notes: Gap: number of days elapsed between the end date of the previous wave and the beginning of the current wave's interviews; n.a.: not applicable, as there was no previous wave.

4. Structure of the Sample

Distribution of Shares

Table 2 shows the overall structure of the sample, disaggregated by wave. The upper panel shows the total number of invitations (including 76 respondents who are redirected from other surveys in the first wave) and disaggregates between those that are rejected and accepted. The ensuing participation or acceptance rate (i.e., the proportion of those who accepted after being invited) is close to ninety percent in all waves.

Table 2 Structure of the Sample

Wave	Wave 1	Wave 2	Wave 3	Wave 4	Sum
Rejected and accepted invitations					
Invited	4,762	2,506	1,892	2,506	11,666
Rejected	589	85	127	181	982
Accepted	4,173	2,421	1,765	2,325	10,684
Participation rate	87.6%	96.6%	93.3%	92.8%	91.6%
Discarded and completed interviews					
Accepted	4,173	2,421	1,765	2,325	10,684
Discarded	1,672	531	106	266	2,575
Declined	0	19	15	26	60
ISO unmet	37	35	21	26	119
Incomplete	259	77	62	116	514
Invalid	0	390	1	45	436
Closed	1,130	10	7	4	1,151
Quota full	246	0	0	49	295
Completed	2,501	1,890	1,659	2,059	8,109
Completion rate	59.9%	78.1%	94.0%	88.6%	75.9%

Source: own elaboration.

Accepted invitations constitute the starting point of the lower panel of the table, and are in turn disaggregated between interviews that are completed and those that are discarded on accounts of different criteria:

a. *Declined participation*: a small fraction of those who had initially accepted the invitation (overall, less than 0.6%) declined to participate after learning the goals

of the questionnaire or the institution responsible for the study.

b. *ISO unmet*: some interviews (overall, 1.1% of those who had accepted to participate) where discarded because they failed to meet ISO quality standards. Participations are labelled as “ISO unmet” when they fail to meet at least one of the following criteria: 1) the information on gender or age provided in the survey is not consistent with the one previously available in the database; 2) the response time is considered as fraudulent, i.e., the survey is completed in less than 20% of the estimated time; 3) the individuals failed to pass an attention check or ‘trick’ question.

c. *Uncompleted interview*: a somewhat larger number of interviews (overall, 514, i.e., 4.8% of those who had accepted to participate) were discarded because they were not fully completed.

d. *Invalidated interview*: a similar figure (436 or 4.1% of those who had accepted to participate) were discarded due to software issues (i.e. the program did not save the answers to some questions)

e. *Closed*: by far, the largest group of discarded interviews (1,151 or 10.8% of those who had accepted to participate) was made up of those who completed the interview but did so only after the field had been closed.

f. *Quota full*: finally, 295 interviews (2.8% of those who had accepted to participate) were discarded because the quota for a respondent’s profile had been already filled.

The completion rate (i.e., the proportion of those who successfully completed the survey after accepting the invitation) ranges from 59.9% in the first wave to 94.0% in the third one, with an average of 75.9%.

Attrition

The samples for individual waves range from 1,659 completed interviews in wave 3 to 2,501 in wave 1. Attrition across waves is reported in Table 3.

The four waves were initially designed to be successively nested. The 2,501 completed interviews in wave 1 is also the cumulative number of completed interviews at this stage. Wave 2 was effectively nested in wave 1. Therefore, all those who completed wave 2 (1,890) had also completed wave 1. This means that 1,890 is also the figure of *consecutively completed interviews* (i.e., of those who completed the current wave, in this case, wave 2, and the immediately previous wave, in this case, wave 1). Moreover, 1,890 is also the number of *cumulatively completed interviews* (i.e., of those who completed the current wave and all the previous ones).

Again, wave 3 was effectively nested in wave 2, meaning that the number of completed interviews in wave 3 (1,659) is also the number of consecutively completed interviews at this stage and, given that wave 2 was in turn was nested in wave 1, it is also the number of cumulatively completed interviews.

Unfortunately, due to time constrains for the proximity of the National elections, not all the participants in wave 2 were contacted in wave 3. Thus, to avoid the risk of a low number of respondents in wave 4, all those who had participated in wave 1 were re-contacted in wave 4, which is therefore not nested in the two previous waves. While 2,059 individuals completed wave 4, only 1,484 of them had also completed wave 3. Thus, despite the important increase of participants in wave 4, given the nesting of wave 3 in wave 2 and of wave 2 in wave 1, the number of panellists who completed the four waves is only 1,484.

Table 3 Wave Attrition

Wave	Wave 1	Wave 2	Wave 3	Wave 4
Completed	2,501	1,890	1,659	2,059
Consecutive completion	n.a.	1,890	1,659	1,484
Immediate permanence rate	n.a.	75.6%	87.8%	89.5%
Cumulative completion	2,501	1,890	1,659	1,484
Cumulative permanence rate	100.0%	75.6%	66.3%	59.3%

Source: own elaboration.

Notes: Completed = accepted – (declined + ISO unmet + incomplete + invalid + closed + quota full). Immediate permanence rate = consecutive completion / completed. Cumulative permanence rate = cumulative completion / completed in wave 1. n.a.: not applicable.

Quota Distribution

Sampling quotas were applied to ensure that the sample reflects the characteristics of the general population in terms of region of residency, gender, and age (the quotas were derived from Spanish official statistics). Table 4 displays the main socio-demographic characteristics of the participants, by wave.

Table 4 Socio-Demographic Characteristics of the Participants, by Wave

Characteristics	Target	Wave 1 Pct/N	Wave 2 Pct/N	Wave 3 Pct/N	Wave 4 Pct/N
**Sex**					
Man	49.80	50.70	51.27	52.08	52.02
		(1,268)	(969)	(864)	(1,071)
Woman	50.20	49.30	48.73	47.92	47.98
		(1,233)	(921)	(795)	(988)
Total	100.00	100.00	100.00	100.00	100.00
		(2,501)	(1,890)	(1,659)	(2,059)
**Age group**					
18_24	9.60	9.84	8.31	7.05	6.99
		(246)	(157)	(117)	(144)
25_34	17.20	19.03	19.15	18.14	19.67
		(476)	(362)	(301)	(405)
35_44	23.50	24.23	23.97	23.93	24.53
		(606)	(453)	(397)	(505)
45_54	21.70	22.23	22.38	23.03	22.78
		(556)	(423)	(382)	(469)
55_+	28.00	24.67	26.19	27.85	26.03
		(617)	(495)	(462)	(536)
Total	100.00	100.00	100.00	100.00	100.00
		(2,501)	(1,890)	(1,659)	(2,059)
**Region**					
Andalucía	18.10	18.11	18.31	18.02	17.97
		(453)	(346)	(299)	(370)
Aragón	2.80	2.76	2.91	2.83	2.57
		(69)	(55)	(47)	(53)
Asturias	2.30	2.32	1.90	1.81	2.48
		(58)	(36)	(30)	(51)
Islas Baleares	2.50	2.08	1.90	1.75	2.04

Characteristics	Target	Wave 1<br>Pct/N	Wave 2<br>Pct/N	Wave 3<br>Pct/N	Wave 4<br>Pct/N
		(52)	(36)	(29)	(42)
Canarias	4.80	3.96	4.18	4.22	3.74
		(99)	(79)	(70)	(77)
Cantabria	1.30	1.32	1.06	1.02	1.36
		(33)	(20)	(17)	(28)
Castilla y León	5.30	5.40	5.71	6.09	5.93
		(135)	(108)	(101)	(122)
Castilla-La Mancha	4.40	4.04	3.86	3.80	3.74
		(101)	(73)	(63)	(77)
Cataluña	15.80	15.99	15.71	16.03	15.83
		(400)	(297)	(266)	(326)
Comunidad Valenciana	10.80	10.80	10.95	10.85	10.93
		(270)	(207)	(180)	(225)
Extremadura	2.40	2.56	2.75	2.71	2.23
		(64)	(52)	(45)	(46)
Galicia	5.90	6.20	5.77	6.09	5.83
		(155)	(109)	(101)	(120)
Madrid	13.80	14.55	14.71	14.23	14.96
		(364)	(278)	(236)	(308)
Murcia	3.10	3.16	3.44	3.68	3.45
		(79)	(65)	(61)	(71)
Navarra	1.40	1.40	1.32	1.39	1.31
		(35)	(25)	(23)	(27)
País Vasco	4.60	4.56	4.55	4.64	4.91
		(114)	(86)	(77)	(101)
La Rioja	0.70	0.80	0.95	0.84	0.73
		(20)	(18)	(14)	(15)
**Total**	100.00	100.00	100.00	100.00	100.00
		(2,501)	(1,890)	(1,659)	(2,059)

Source: own elaboration.

5. Coding, Naming, and Labelling Protocols

Information in the dataset follows a series of protocols to optimize the size of the database and to facilitate the users' access to and understanding of the information. The following subsections share the naming, labelling, and coding protocols employed in the E-DEM database.

Coding of Missing, Non-Response and Non-Applicable values

Uncertain responses (i.e. "don't know", "I prefer not to answer") have received special treatment. For starters, the surveys refrained for explicitly providing "decline to response" options. Instead, participants were allowed to skip the question. The use of "don't know" options was limited to knowledge questions. Finally, a pop-up alert was established to confirm no opinion responses.

The coding of non-response categories ("does not know", "does not answer", "does not apply / not applicable", "belongs to the control group of an experiment", and "not re-contacted in a given wave") has been standardised for all the questions in the database, so that each type of missing response receives a unique code throughout the database and that code is not used for any other purpose. Their labelling has followed equally systematic criteria. The coding and labelling protocols are as follows:

- Does not know: coded as ".a", labelled as "[DK]".
- Does not answer: coded as ".b", labelled as "[DA]"
- Does not apply: coded as ".c", labelled as "[NA]".
- Belongs to the control group of an experiment: coded as ".y", labelled as "[NA: control group]".
- Not re-contacted in a given wave: codes as ".z", labelled as "[NA: not in wave]".

Notice that when labels are applied for these categories, they always appear between square brackets, to mark visually that they refer to non-response categories. Of note also is that the two latter codes, "." and ".a", are different types of missing values, and hence are not considered by most Stata commands unless otherwise and explicitly specified.

Protocol for Naming Variables

The variable naming is structured in three different parts:

- A prefix letter, indicating the group to which the variable belongs.
- The variable number.
- A suffix, indicating the wave to which the variable belongs.

First, **the prefix letter indicates the group** to which the variable belongs. The database distinguishes between five groups of non-experimental variables:

- **"g" = global** variables, which apply to all waves, such as the panellists' unique identification numbers.
- **"w" = wave-specific** variables, which capture characteristics of the interviews carried out in each wave, such as the interview's duration.
- **"s" = sociodemographic** variables.
- **"p" = all the other** opinion questions, except for the "trust" battery (see right below).
- **"trust" =** battery of **trust in political institutions non-experimental** variables (asked in waves 2, 3, and 4), based on the same battery as the "et" experimental variables of experiment 1, which was conducted in wave 1 (see below; bear in mind that other trust measures, i.e., those that do not relate to this battery of political institutions, are included among the "p" variables).

The E-DEM database also includes a series of experimental variables. All their prefixes start with "e":

- **"et" = experimental variables of experiment 1** (conducted in wave 1) to measure trust in a battery of political institutions.
- **"em" = experimental variables of experiment 2** (conducted in wave 2), on ideological preferences, selective exposure to (traditional) media political information, framing and affective polarisation.
- **"esm" = experimental variables of experiment 3 and experiment 4** (conducted in wave 3 and 4, respectively), on ideological preferences, selective exposure to social media and affective polarisation in *national* elections (experiment 3) and *European* elections (experiment 4).

Second, **the numbers given to the variables in each group are organized in numerical order within each of the groups**: s1, s2, s3, s4, and so on for the "s" variables; p1, p2, p3, p4, and so on for the "p" variables, etcetera. Variables that are related receive the same number, plus a letter to differentiate them:

- **Lowercase letters are assigned in alphabetical order to differentiate among different variables pertaining to a battery of questions**, i.e., "p7a" (PP ideology), "p7b" (PSOE ideology), "p7c" (UP ideology), and so on. This

convention is also applied to closely related questions, i.e., “s14” (belongs to a religion), “s14a” (religious denomination), “s14b” (church attendance).

- **An upper case “R” is added for recoded variables**, i.e., “s2R_1” (age group).
- **An upper case “T” is added for time variables**, measuring the time spent to answer certain questions, i.e., “p36aT_1” (TIME to answer p36a).
- **An upper case “V” is added for follow-up questions** (together with the value of the “others” category motivating them), i.e., “p81V24” (Vote intention for the May 26th Regional Elections, which other; in “p81”, value 24 = “others”).
- **An upper case “P” is added for all the post-experimental variables**.

Third and finally, **all the variables have a suffix whose number reflects the wave** of the panel to which that question belongs (“_1”; “_2”; “_3”; “_4”). The (few) global variables in the “g” group are assigned suffix “_0” to mark the fact that they refer to the database as a whole, instead of to any specific wave.

Taking all this into account, Table 5 displays some examples of variable names, also indicating their meaning and the group and wave to which they pertain. When adequate, a clarifying comment is also included:

Table 5 Examples of Variable Names (Non-Experimental Variables)

**Variable**	**Meaning**	**Group**	**Wave**	**Comment**
Standard non-experimental variables				
g1d_0	Panellist unique numerical Id	“g”	“0”	
w3_1	Interview's duration in seconds	“w”	1	
s1_1	Sex	“s”	1	
p1_2	Political interest	“p”	2	
trust1a_2<br>trust1b_3<br>trust1j_4	Trust in the Spanish Parliament<br>Trust in the Spanish Government<br>Trust in the European Commission	“trust”	2<br>3<br>4	non-experimental replicas of experiment 1
Related variables (filter, recoded, sequence, time, and value variables)				
s2_3<br>s2**R**_3	Age<br>Age group	“s”	3	Recoded variable
p36e_1<br>p36e**T**_1	Norway is a member of the EU<br>TIME to answer p36e	“p”	1	Time variable
p80_4<br>p80**V11**_4	Vote intention, May 26th EP Elections<br>Vote intention, EP Elections, which other	“p”	4	Value variable

Source: own elaboration.

Table 6 displays examples of names of experimental (and post-experimental) variables, together with their meaning, group, and wave:

Table 6 Examples of Variable Names (Experimental Variables)

Variable	Meaning	Group	Wave	Comment
Experimental variables				
et0_1	Group in experiment 1	"et"	1	Experiment 1
et1a_1	Trust in the Spanish Parliament	"et"	1	Experiment 1 group 1 (control group)
et6a_1	Trust in the Spanish Parliament	"et"	1	Experiment 1 group 6 (treatment 5)
em0_2	Group in experiment 2	"em"	2	Experiment 2
esm0_3	Group in experiment 3	"esm"	3	Experiment 3
esm0_4	Group in experiment 4	"esm"	4	Experiment 4
Experiments: post-experimental variables				
et6P2_1	Institutions alluded in judicial news	"et"	1	Experiment 1, group 5
et6P2_1	Institutions alluded in judicial news	"et"	1	Experiment 1, group 6
emP2_2	News' topic	"em"	2	Experiment 2
esmP6a_3<br>esmP6c_3	Hashtag associated to Pablo Casado<br>Hashtag associated to Pablo Iglesias	"esm"	3	Experiment 3
esmP9_4	Topics discussed in the account	"esm"	4	Experiment 4

Source: own elaboration.

Protocol for Labelling Variables

Variable labeling seeks a balance between being informative and not being excessively long. None of them includes abbreviations in the names (party labels instead of party names are used, though).

Given that the variables' names all include information on the wave, this information is not repeated in the variables' labels. Thus, for any given variable available in different waves, all the variable labels are the same. For instance, "s8_1", "s8_2", "s8_3", and "s8_4" are all labelled as "Occupation".

Protocol for Labelling Variable Values

Protocol of assignment of value labels to variables:

The assignment or not of value labels follows a precise protocol in the E-DEM dataset.

1. *If a variable includes non-response categories, it will at least have a generic value label to clarify the meaning of those responses* (i.e., to clarify that 888 means “does not know”). The most usual non-response categories are “does not know”, “does not apply”, and “does not remember”. This rule takes precedence over all the others, irrespective of the type of variable involved.
2. *Quantitative variables and scales of ten or more values have no value labels* (except if they include non-response categories). In particular, we have not assigned value labels to any variable for the sole sake of clarifying its polarity. Thus, instead of having a label informing only of the meaning of the two extremes of its eleven-point scale, "p13a_1” (trust in family) has a note stating that “0 = Do not trust at all, 10 = Trust Completely".
3. *Ordinal variables of seven categories or more in which the categories are degrees of intensity of a property with no specific substantive meaning do not have value labels either* (as long as they do not include non-response categories). For instance, “trust1a_4” has no value labels but a note clarifying that "1 = No trust at all, 7 = Complete trust".
4. *Ordinal variables always have value labels when each of the categories of the scale has a substantive meaning*. This is the case, for instance, of “s14b_1” (church attendance). Its seven response categories all have a substantive meaning, so it has a value label spelling out those meanings (0 = “never”, 1 = “only occasionally”, 2 = “only on special holidays”, and so on).
5. *Ordinal variables of six categories or less, nominal variables and binary variables always have value labels*, as information on the meaning of each response category of these variables is always necessary.

Variables of different waves share a common value label, instead of each one of them having their own, but identical, value labels. For instance, variables “s1_1”, “s1_2”, “s1_3”, and “s1_4” (sex) share a common value label.

Variable-specific value labels take the name of the variables they refer to, but without the suffix indicating the wave. For instance, the common value label for the sex variables above is named simply as “s1”.

A considerable large fraction of the E-DEM dataset requires the same value labels. Instead of creating them many times with many different names, the following **generic label values** have been created to label “yes/no” responses, “agreement-disagreement” responses, and “does not know”, “does not apply “responses:

- “dkda” (.a = “[DK]”, .b = “[DA]”, .c = “[NA]”, .y = “[NA: control group]”, .z = “[NA: not in wave]”)
- “yndk” (1 = "Yes", 2 = "No", + “dkda” value labels)

- “nydk” (0 = "No", 1 = "Yes", + “dkda” value labels)
- “agree5i” (1 = "Agree strongly", 2 = "Somewhat agree", 3 = "Neither agree nor disagree", 4 = "Somewhat disagree", 5 = "Disagree strongly", + “dkda” value labels)

Notice that the “yndk”, “nydk” and “agree5i” generic value labels all include their own specific value labels plus those of the “dkda” generic value labels; for instance, the “yndk” also includes value labels to clarify that .a = “[DK]”, .b = “[DA]”, and so on.

Naming and Labelling Language

Variable names, variable labels and value labels are all in English except when they refer to proper nouns, such as the names of regions (i.e., Andalucía) and politicians (i.e., Pedro Sánchez) or the abbreviations of political parties’ names (i.e., UP, for Unidas Podemos), which are maintained in Spanish.

6. Variable List

In this section, the complete list of non-behavioural variables available in the integrated dataset (i.e., of non-experimental survey variables retrieved in one or more of the four waves of the panel surveys as well as of experimental and post-experimental variables) is presented.

The list of variables is presented in tables, whereby the first column includes information on the variable names (when a variable is available in several waves, only the name of the first wave in which it appears is displayed), the second column displays the value label names (for all the variables that have value labels), the third column shows the variable labels (which clarify the contents of the variables), and columns four through seven inform of the wave or waves in which each variable is available (a capital “X” in a variable * wave cell indicates that the variable is available in the wave, and a blank space means that it is not).

To facilitate the navigation through the variable list, the information is presented in a series of tables, each of which referring to one group of variables: Table 7, list of “global” or “g” variables (with information on general characteristics of the dataset); Table 8, list of “wave” or “w” variables (interview’s characteristics in each wave); Table 9, list of “socio-demographic” or “s” variables (participants’ socio-demographic and socio-economic characteristics); Table 10, list of “opinion” and other “p” variables (broad range of opinions, attitudes, beliefs, evaluations, reported and intended behaviour of participants); Table 11, list of “trust” variables (non-experimental variables on trust in political institutions); Table 12, list of “et” variables (first experiment); Table 13, list of “em” variables (second experiment); Table 14, list of “ems” variables (third experiment) and Table 15, list of “ems” variables (fourth experiment).

Global Variables

Table 7 shows the list of global variables, which contain information on general characteristics of the survey. There are only six global variables, including the panelist' unique id number, a longer unique id used by *Netquest*, three variables informing of whether the panellist completed the second, third and four waves (remember that all the panellists in the E-DEM dataset completed wave 1) and a variable informing of whether the panellist completed all the four waves or not. The six global variables end with the "_0" suffix:

Table 7 List of Global Variables

**Variable name**	**Value label**	**Variable label**
g1a_0		Panellist unique Id
g1b_0		Panellist unique long Id
g2_0	nydk	Completed wave 2 (nested in wave 1)
g3_0	nydk	Completed wave 3 (nested in waves 2 & 1)
g4_0	nydk	Completed wave 4 (nested only in wave 1)
g4a_0	nydk	Completed all waves (w3 nested in w2 & w1)

Source: own elaboration.

Wave-Specific Variables

Table 8 shows the list of wave-specific variables, which contain information on the interview's characteristics in each wave. There are five wave-specific variables per wave (the interview's access count, start and end days, duration in seconds, and the device used for the interview), and they are all available for the four waves:

Table 8 List of Wave-Specific Variables

**Variable**	**Value label**	**Variable label**	**W1**	**W2**	**W3**	**W4**
w1_1		Number of accesses to the interview (access count)	X	X	X	X
w2a_1		Interview's start day	X	X	X	X
w2b_1		Interview's end day	X	X	X	X
w3_1		Interview's duration in seconds	X	X	X	X
w4_1	w4	Device used for the interview	X	X	X	X

Source: own elaboration.

Notes: variable names of wave 1 shown in the first column; the names for the other waves only differ as regards the wave suffix.

Socio-Demographic Variables

Table 9 shows the list of socio-demographic and socio-economic variables. Some of them are available in all the waves. The ones on top are the filtering variables (region, sex, and age), size of habitat and education. Additionally, we have included in all the waves questions s8-s11a, which could vary overtime. All of the remaining socio-demographic variables (like marital status, number of children, or religious belonging, denomination and attendance) have only been asked in the first wave, as they do not tend to vary much in the short six-months span in which the four surveys took place:

Table 9 List of Socio-Demographic Variables

Variable	Value label	Variable label	W1	W2	W3	W4
s0_1	s0	Region	X	X	X	X
s1_1	s1	Sex	X	X	X	X
s2_1		Age	X	X	X	X
s2R_1	s2R	Age group	X	X	X	X
s3a_1	s3a	Habitat (number of inhabitants)	X	X	X	X
s3b_1	s3b	Place of residence	X			
s4a_1	s4a	Education, 8 levels	X	X	X	X
s4b_1	s4b	Education, 28 categories	X			
s4bV27_1		Education, which other	X			
s5_1	s5	Marital status	X			
s6_1		Number of children	X			
s7_1		Number living in household	X			
s8_1	s8	Occupation	X	X	X	X
s9_1	s9	Subjective wellbeing	X	X	X	X
s10_1	yndk	Lost job in last year	X	X	X	X
s11a_1	s11a	Concern about bills	X	X	X	X
s11b_1	s11b	Concern about reducing lifestyle	X	X	X	X
s11c_1	s11c	Concern about getting a job	X	X	X	X
s11d_1	s11d	Concern about loans and mortgages	X	X	X	X
s12_1	s12	Income	X			
s12a_1	s12a	Expresses income in weeks, months, or years	X			
s13_1		Satisfaction with household economy	X			

Variable	Value label	Variable label	W1	W2	W3	W4
s14_1	nydk	Belongs to a religion	X			
s14a_1	s14a	Religious Denomination	X			
s14b_1	s14b	Church attendance	X			

Source: own elaboration.

Notes: variable names of wave 1 shown in the first column; the names for the other waves only differ as regards the wave suffix.

Opinion, Attitudinal and Beliefs Variables

Table 10 shows the list of opinion, attitudinal and beliefs variables, i.e., of all the variables that belong to the "p" variables.

Some of them are available in all the waves, some others are available in several waves, and yet some others are only available in a given wave. For instance, the question on political interest is available in the four waves ("p1_1", "p1_2", "p1_3", "p1_4"); the question on whether the respondent signed a petition is available in waves 1 and 2 ("p32a_1" and "p32a_2"), and the same is true for the other questions of the battery on non-electoral political participation. The questions of the political efficacy battery are only available in the first wave (this is the case of "p16e_1", which captures the respondent's perception on how much do politicians care about people's opinions), and the battery on membership and participation in associations is only available in the second wave (this is the case of "p39a1_2", on whether the respondent belongs to a church). In the "variable name" column, we have always chosen to display the name of the variable in the earliest wave in which it appears (for instance, for political interest, we display the name of the first wave, "p1_1").

Finally, many of the questions belong to batteries. Whenever this is the case, we have remarked it in the table by (a) introducing a row before the first question of the battery indicating the topic of the battery; and (b) adding a column in Table 10 to the left of the variable's name where the name of the battery is clearly indicated.

Notice that some of the battery questions may have been only asked in some waves. For instance, the battery on Spain's economic and social situation: whereas the first six questions of the battery (on the unemployment, education, healthcare, immigration, pensions and corruption situations, respectively) are asked since the first wave through the fourth one, the seventh item of the battery (on the situation of violence against women) is introduced in the second wave and maintained in the third and fourth waves, while the eighth and last item (on the situation in Catalonia) is introduced even later, in the third wave.

Table 10 List of Opinion and other "p" Variables

Battery	Variable name	Value label	Variable label	W1	W2	W3	W4
	p1_1	p1	Political Interest	X	X	X	X
	p2_1		Satisfaction with Economy	X	X	X	X
	p3_1	p3	Spain's main problem	X	X	X	X
	p3V19_1		Spain's main problem, which other problem	X	X	X	X
BATTERY: Spain's economic and social situation							
p4 battery	p4a_1	dkda	Unemployment situation	X	X	X	X
	p4b_1	dkda	Education situation	X	X	X	X

Battery	Variable name	Value label	Variable label	W1	W2	W3	W4
	p4c_1	dkda	Healthcare situation	X	X	X	X
	p4d_1	dkda	Immigration situation	X	X	X	X
	p4e_1	dkda	Pensions situation	X	X	X	X
	p4f_1	dkda	Corruption situation	X	X	X	X
	p4g_2	dkda	Situation of violence against women		X	X	X
	p4h_3	dkda	Situation in Catalonia			X	X
	p5_1	dkda	Satisfaction with Democracy in Spain	X	X		
	p6_1	dkda	Ideology	X	X	X	
BATTERY: ideology attributed to parties							
p7 battery	p7a_1	dkda	PP Ideology	X	X	X	
	p7b_1	dkda	PSOE Ideology	X	X	X	
	p7c_1	dkda	UP Ideology	X	X	X	
	p7d_1	dkda	IU Ideology	X	X		
	p7e_1	dkda	Cs Ideology	X	X	X	
	p7f_1	dkda	ERC Ideology	X	X	X	
	p7g_1	dkda	PDeCAT Ideology	X	X	X	
	p7h_1	dkda	EAJ-PNV Ideology	X	X	X	
	p7i_1	dkda	EH-Bildu Ideology	X	X	X	
	p7j_1	dkda	GBAI Ideology	X	X		
	p7k_1	dkda	BNG Ideology	X	X		
	p7l_2	dkda	VOX Ideology		X	X	
	p7m_3	dkda	Compromís Ideology			X	
	p7n_3	dkda	CC Ideology			X	
	p7o_3	dkda	En Marea Ideology			X	
	p8_1	dkda	Opinion on government decentralisation	X	X	X	X
BATTERY: parties' positions on decentralization							
p9 battery	p9a_1	dkda	PP position on decentralisation	X	X	X	X
	p9b_1	dkda	PSOE position on decentralisation	X	X	X	X
	p9c_1	dkda	UP position on decentralisation	X	X	X	X
	p9d_1	dkda	IU position on decentralisation	X	X		
	p9e_1	dkda	Cs position on decentralisation	X	X	X	X

Battery	Variable name	Value label	Variable label	W1	W2	W3	W4
	p9f_1	dkda	ERC position on decentralization	X	X	X	X
	p9g_1	dkda	PDeCAT position on decentralisation	X	X	X	X
	p9h_1	dkda	PNV position on decentralisation	X	X	X	X
	p9i_1	dkda	EH-Bildu position on decentralisation	X	X	X	X
	p9j_1	dkda	GBAI position on decentralisation	X	X		
	p9k_1	dkda	BNG position on decentralisation	X	X		
	p9l_2	dkda	VOX position on decentralisation		X	X	X
	p9m_3	dkda	CC position on decentralisation			X	X
	p9n_3	dkda	Compromís position on decentralisation			X	X
	p9o_3	dkda	En Marea position on decentralisation			X	X
BATTERY: respondents' opinions on social issues							
p10 battery	p10a_1	Dkda	Opinion on cultural assimilation of migrants	X	X	X	X
	p10b_1	Dkda	Opinion on State intervention in the economy	X	X	X	X
	p10c_1	Dkda	Opinion on same-sex marriage	X	X	X	X
	p10d_1	Dkda	Opinion on provision of public services	X	X	X	X
	p10e_1	Dkda	Opinion on women abortion right	X	X	X	X
	p10f_1	Dkda	Opinion on fairness of wealth distribution	X	X	X	X
	p10g_1	Dkda	Opinion on women working	X	X	X	X
	p10h_1	Dkda	Opinion on immigration level	X	X	X	X
	p10i_3	Dkda	Opinion on the solution to the Catalan conflict			X	X
BATTERY: feelings towards people from certain regions, voters of certain parties, and party leaders							
p11 battery	p11a_1	Dkda	Feelings towards Basques	X	X	X	X
	p11b_1	Dkda	Feelings towards Catalans	X	X	X	X
	p11c_1	Dkda	Feelings towards Madrileños	X	X	X	X
	p11d_1	Dkda	Feelings towards Andalusians	X	X	X	X
	p11e_1	Dkda	Feelings towards Refugees	X	X	X	X
	p11f_1	dkda	Feelings towards PP voters	X	X	X	X
	p11g_1	dkda	Feelings towards PSOE voters	X	X	X	X
	p11h_1	dkda	Feelings towards Cs voters	X	X	X	X
	p11i_1	dkda	Feelings towards UP voters	X	X	X	X

Battery	Variable name	Value label	Variable label	W1	W2	W3	W4
	p11j_1	dkda	Feelings towards Pablo Casado	X	X	X	X
	p11k_1	dkda	Feelings towards Pedro Sánchez	X	X	X	X
	p11l_1	dkda	Feelings towards Albert Rivera	X	X	X	X
	p11m_1	dkda	Feelings towards Pablo Iglesias	X	X	X	X
	p11n_1	dkda	Feelings towards Íñigo Urkullu	X	X	X	X
	p11o_1	dkda	Feelings towards Carles Puigdemont	X	X	X	X
	p11p_1	dkda	Feelings towards Oriol Junqueras	X	X	X	X
	p11q_2	dkda	Feelings towards Santiago Abascal		X	X	X
	p11r_2	dkda	Feelings towards VOX voters		X	X	X
	p11s_2	dkda	Feelings towards immigrants		X	X	X
BATTERY: respondents' perceptions about other people							
p12 battery	p12a_1	dkda	Trust in people	X	X	X	X
	p12b_1	dkda	Perception about honesty of people	X	X	X	X
	p12c_1	dkda	Perception about social solidarity	X	X	X	X
BATTERY: respondents' trust in relevant others							
p13 battery	p13a_1	dkda	Trust in Family	X	X	X	X
	p13b_1	dkda	Trust in your neighbours	X	X	X	X
	p13c_1	dkda	Trust in people you know personally	X	X	X	X
	p13d_1	dkda	Trust in people you meet for the first time	X	X	X	X
	p13e_1	dkda	Trust in people form another religion	X	X	X	X
	p13f_1	dkda	Trust in people from another nationality	X	X	X	X
	p13g_1	dkda	Trust in Catalans	X	X	X	X
	p13h_1	dkda	Trust in Basques	X	X	X	X
	p13i_1	dkda	Trust in Madrileños	X	X	X	X
	p13j_1	dkda	Trust in Andalusians	X	X	X	X
	p13k_2	dkda	Migrants living in Spain		X	X	X
BATTERY: trust in voters of different political parties							
p14 battery	p14a_1	dkda	Trust in PP voters	X	X	X	X
	p14b_1	dkda	Trust in PSOE voters	X	X	X	X
	p14c_1	dkda	Trust in Cs voters	X	X	X	X

Battery	Variable name	Value label	Variable label	W1	W2	W3	W4
	p14d_1	dkda	Trust in UP & IU voters	X	X	X	X
	p14e_1	dkda	Trust in ERC voters	X	X	X	X
	p14f_1	dkda	Trust in PDeCAT voters	X	X	X	X
	p14g_1	dkda	Trust in EAJ-PNV voters	X	X	X	X
	p14h_2	dkda	Trust in VOX voters		X	X	X
BATTERY: identification with different territorial units							
p15 battery	p15a_1	dkda	Identification with city or town	X	X		
	p15b_1	dkda	Identification with Region	X	X		
	p15c_1	dkda	Identification with Spain	X	X		
	p15d_1	dkda	Identification with Europe	X	X		
BATTERY: political efficacy							
p16 battery	p16a_1	dkda	The system allows to have a say in government decisions	X			
	p16b_1	dkda	Feels able to take an active role in a political group	X			
	p16c_1	dkda	System allows people's influence on politics	X			
	p16d_1	dkda	Self-confidence to participate in politics	X			
	p16e_1	dkda	Politicians care about people's opinions	X			
BATTERY: sources used for political information							
p17 battery	p17a_1	p17a	Use of newspapers for political information	X	X	X	
	p17b_1	p17b	Use of radio for political information	X	X	X	
	p17c_1	p17c	Use of magazines for political information	X	X	X	
	p17d_1	p17d	Use of Tv for political information	X	X	X	
	p17e_1	p17e	Use of social networks for political information	X	X	X	
	p18_1	p18	Use of internet as source of information	X			
BATTERY: whether respondent has accounts on different social networks							
p19 battery	p19a_1	nydk	Twitter account	X	X	X	X
	p19a1_3	yndk	Follows the electoral campaign on Twitter			X	X
	p19b_1	nydk	Facebook account	X	X	X	X
	p19c_1	nydk	Google+ account	X	X	X	X
	p19d_1	nydk	Linkedin account	X	X	X	X

Battery	Variable name	Value label	Variable label	W1	W2	W3	W4
	p19e_1	nydk	Instagram account	X	X	X	X
	p19f_1	nydk	Flickr account	X	X	X	X
	p19g_1	nydk	Youtube account	X	X	X	X
	p19h_1	nydk	Another account	X	X	X	X
	p19hV1_1		Social network, which other account	X	X	X	X
	p19i_3	nydk	Whatsapp account			X	X
BATTERY: frequency of connection to the different social networks							
p20 battery	p20a_1	p20a	Twitter frequency	X			
	p20b_1	p20b	Facebook frequency	X			
	p20c_1	p20c	Google+ frequency	X			
	p20d_1	p20d	Linkedin frequency	X			
	p20e_1	p20e	Instagram frequency	X			
	p20f_1	p20f	Flickr frequency	X			
	p20g_1	p20g	YouTube frequency	X			
	p20h_1	p20h	Another social network frequency	X			
	p21_1	p21	Frequency of political news found on social networks	X			
BATTERY: degree to which internet and social networks help to relate to certain groups of people							
p22 battery	p22a_1	p22a	Internet and social networks help to increase participation in your groups	X			
	p22b_1	p22b	Internet and social networks help to increase participation in new groups	X			
	p22c_1	p22c	Internet and social networks help to relate with people with same interests	X			
	p22d_1	p22d	Internet and social networks help to relate with people with same religion	X			
	p22e_1	p22e	Internet and social networks help to relate with people with same political idea	X			
	p22f_1	p22f	Internet and social networks help to relate with people from different cultures	X			
	p22g_1	p22g	Internet and social networks help to relate with people of different ages	X			
	p22h_1	p22h	Internet and social networks help to relate with people from different countries	X			
	p23a_1	dkda	Affinity with political opinions found on Twitter	X			
	p23b_1	p23b_1	Ideology of opinions found on Twitter	X			
	p24a_1	dkda	Affinity with political opinions found on	X			

Battery	Variable name	Value label	Variable label	W1	W2	W3	W4
			Facebook				
	p24b_1	dkda	Ideology of opinions found on Facebook	X			
	p25_1	p25	Political accounts followed on Twitter	X			
	p26a_1	p26a	Frequency of political discussions with family and friends	X	X	X	
	p26b_1	p26b	Frequency of political discussions on social networks	X	X	X	X
	p27a_1	p27a	Frequency of agreement when discussing about politics	X	X	X	
	p27b_1	p27b	Frequency of agreement when discussing about politics on social networks	X	X	X	X
	p28a_1	p28a	Frequency of disagreement when discussing about politics	X	X	X	
	p28b_1	p28b	Frequency of disagreement when discussing about politics on social networks	X	X	X	X
	p29a_1	p29a	Political preferences of people I discuss politics with	X	X	X	
	p29b_1	p29b	Political preferences of people I discuss politics with on social networks	X	X	X	X
	p30_1	yndk	Voted in June 2016 national election	X			
	p30a_1	p30a	Vote recall of the June 2016 National Elections	X			
	p30aV11_1		Vote recall of the June 2016 National Elections, which other	X			
	p31_1	p31	Vote intention if you had voted in the last National Elections	X			
	p31V11_1		Vote intention if you had voted in the last National Elections, which other	X			
BATTERY: non-electoral political participation							
p32 battery	p32a_1	p32a	Signed a petition	X	X		
	p32b_1	p32b	Boycotted certain products	X	X		
	p32c_1	p32c	Displayed a campaign badge/sticker	X	X		
	p32d_1	p32d	Took part in a lawful public demonstration	X	X		
	p32e_1	p32e	Took part in a political rally	X	X		
	p32f_1	p32f	Contacted a politician or government official	X	X		
	p32g_1	p32g	Contacted or appeared in the media	X	X		
BATTERY: online political participation							
p33 battery	p33a_1	nydk	Visited the web page of a political party, group, or candidate	X	X		
	p33b_1	nydk	Followed on Facebook a political party,	X	X		

Battery	Variable name	Value label	Variable label	W1	W2	W3	W4
			group, or candidate				
	p33c_1	nydk	Sent an email to a political party, group, or candidate	X	X		
	p33d_1	nydk	Tweeted to a political party, group, or candidate	X	X		
	p33e_1	nydk	Posted about politics on social media	X	X		
	p33f_1	nydk	Debated about politics in a forum or blog	X	X		
	p33g_1	nydk	Signed an online petition	X	X		
	p33h_1	nydk	Used internet to persuade people to vote	X	X		
	p33i_1	nydk	Used social networks to persuade people whom to vote for	X	X		
	p33j_1	nydk	Used email to persuade people whom to vote for	X	X		
	p33k_1	nydk	Took part in a political event which invitation you got by internet	X	X		
	p33l_1	nydk	Took part of viral campaigns based on political irony on Tw or Fb	X	X		
BATTERY: online political reactions							
p34 battery	p34_2	dkda	Activities during the last 12 months		X		
	p34a_1	p34a	Started following people with different political opinions	X	X		
	p34b_1	p34b	Marked as liked a political comment or tweet	X	X		
	p34c_1	p34c	Shared political comments or tweets posted by others	X	X		
	p34d_1	p34d	Commented positively a political tweet or Fb post from others	X	X		
	p34e_1	p34e	Commented negatively a political tweet or Fb post from others	X	X		
	p34f_1	p34f	Unfollowed or blocked contacts for political reasons	X	X		
	p34g_1	p34g	Decided not to post political content to avoid offending others	X	X		
	p34h_1	p34h	Decided not to post political content to avoid public exposure	X	X		
	p34i_1	p34i	Changed your mind after taking part on a Fb or Tw political discussion	X	X		
	p34j_1	p34j	Increased political participation after a debate on social networks	X	X		
	p34k_1	p34k	Decreased political participation after a debate on social networks	X	X		
BATTERY: party identity							
p35 battery	p35_1	nydk	Party identity	X	X	X	X
	p35a_1	p35a	Party id specification	X	X	X	X

Battery	Variable name	Value label	Variable label	W1	W2	W3	W4
	p35aV12_1		Party id others	X	X	X	X
	p35b_1	p35b	Degree of closeness to political party	X	X	X	X
	p35c_1	p35c	Importance of Party id	X	X	X	
	p35d_1	p35d	How well does your Party id describe you	X	X	X	
	p35e_1	p35e	Use of We when referring the political party of your preference	X	X	X	
	p35f_1	p35f	Feels as part of the political party	X	X	X	
BATTERY: political knowledge battery, plus time to answer the political knowledge questions							
p36 battery	p36a_1	p36a	Margarita Robles is the Spanish Minister of Defence	X			
	p36aT_1		TIME to answer p36a (in seconds)	X			
	p36b_1	p36b	The Spanish Congress has 525 members	X			
	p36bT_1		TIME to answer p36b (in seconds)	X			
	p36c_1	p36c	To run in the Spanish National Elections you must be at least 25 years old	X			
	p36cT_1		TIME to answer p36c (in seconds)	X			
	p36d_1	p36d	EU countries elect the same number of MPs to the EP	X			
	p36dT_1		TIME to answer p36d (in seconds)	X			
	p36e_1	p36e	Norway is a member of the EU	X			
	p36eT_1		TIME to answer p36e (in seconds)	X			
	p36f_1	p36f	Including Great Britain, there are 28 EU member countries	X			
	p36fT_1		TIME to answer p36f (in seconds)	X			
	p37a_2	p37a	Spanish economic situation in the last 12 months		X	X	X
	p37b_2	p37b	Household economic situation in the last 12 months		X	X	X
	p38a_2	dkda	Satisfaction with government		X	X	
	p38b_2	dkda	Satisfaction with PP as the main opposition party		X	X	
BATTERY OF BATTERIES: belonging to and participation in associations and organisations							
SUB-BATTERY: belonging to and participation in church							
p39a battery	p39a0_2	nydk	Does not belong to a church		X		
	p39a1_2	nydk	Belongs to a church		X		
	p39a2_2	nydk	Took part in activities of a church		X		
	p39a3_2	nydk	Donated to a church		X		

Battery	Variable name	Value label	Variable label	W1	W2	W3	W4
	p39a4_2	nydk	Volunteered in a church		X		
	p39a9_2	nydk	DK/DA, a church		X		
SUB-BATTERY: belonging to and participation in a sports organization							
p39b battery	p39b0_2	nydk	Does not belong to a sports organization		X		
	p39b1_2	nydk	Belongs to a sports organization		X		
	p39b2_2	nydk	Took part in activities of a sports organization		X		
	p39b3_2	nydk	Donated to a sports organization		X		
	p39b4_2	nydk	Volunteered in a sports organization		X		
	p39b9_2	nydk	DK/DA, a sports organization		X		
SUB-BATTERY: belonging to and participation in a music organization							
p39c battery	p39c0_2	nydk	Does not belong to an educational, art or music organization		X		
	p39c1_2	nydk	Belongs to an educational, art or music organization		X		
	p39c2_2	nydk	Took part in activities of an educational, art or music organization		X		
	p39c3_2	nydk	Donated to an educational, art or music organization		X		
	p39c4_2	nydk	Volunteered in an educational, art or music organization		X		
	p39c9_2	nydk	DK/DA, an educational, art or music organization		X		
SUB-BATTERY: belonging to and participation in a labour union							
p39d battery	p39d0_2	nydk	Does not belong to a labour union		X		
	p39d1_2	nydk	Belongs to a labour union		X		
	p39d2_2	nydk	Took part in activities of a labour union		X		
	p39d3_2	nydk	Donated to a labour union		X		
	p39d4_2	nydk	Volunteered in a labour union		X		
	p39d9_2	nydk	DK/DA, a labour union		X		
SUB-BATTERY: belonging to and participation in a political party							
p39e battery	p39e0_2	nydk	Does not belong to a political party		X		
	p39e1_2	nydk	Belongs to a political party		X		
	p39e2_2	nydk	Took part in activities of a political party		X		
	p39e3_2	nydk	Donated to a political party		X		
	p39e4_2	nydk	Volunteered in a political party		X		

Battery	Variable name	Value label	Variable label	W1	W2	W3	W4
	p39e9_2	nydk	DK/DA, a political party		X		
SUB-BATTERY: belonging to and participation in an environmental organization							
p39f battery	p39f0_2	nydk	Does not belong to an environmental organization		X		
	p39f1_2	nydk	Belongs to an environmental organization		X		
	p39f2_2	nydk	Took part in activities of an environmental organization		X		
	p39f3_2	nydk	Donated to an environmental organization		X		
	p39f4_2	nydk	Volunteered in an environmental organization		X		
	p39f9_2	nydk	DK/DA, an environmental organization		X		
SUB-BATTERY: belonging to and participation in a professional organization							
p39g battery	p39g0_2	nydk	Does not belong to a professional association		X		
	p39g1_2	nydk	Belongs to a professional association		X		
	p39g2_2	nydk	Took part in activities of a professional association		X		
	p39g3_2	nydk	Donated to a professional association		X		
	p39g4_2	nydk	Volunteered in a professional association		X		
	p39g9_2	nydk	DK/DA, a professional association		X		
SUB-BATTERY: belonging to and participation in a charitable or humanitarian organization							
p39h battery	p39h0_2	nydk	Does not belong to a charitable or humanitarian organization		X		
	p39h1_2	nydk	Belongs to a charitable or humanitarian organization		X		
	p39h2_2	nydk	Took part in activities of a charitable or humanitarian organization		X		
	p39h3_2	nydk	Donated to a charitable or humanitarian organization		X		
	p39h4_2	nydk	Volunteered in a charitable or humanitarian organization		X		
	p39h9_2	nydk	DK/DA, a charitable or humanitarian organization		X		
SUB-BATTERY: belonging to and participation in a consumer's organization							
p39i battery	p39i0_2	nydk	Does not belong to a consumers' organization		X		
	p39i1_2	nydk	Belongs to a consumers' organization		X		
	p39i2_2	nydk	Took part in activities of a consumers' organization		X		
	p39i3_2	nydk	Donated to a consumers' organization		X		

Battery	Variable name	Value label	Variable label	W1	W2	W3	W4
	p39i4_2	nydk	Volunteered in a consumers' organization		X		
	p39i9_2	nydk	DK/DA, a consumers' organization		X		
SUB-BATTERY: belonging to and participation in an aid and social services organization							
p39l battery	p39l0_2	nydk	Does not belong to an aid and social services organization		X		
	p39l1_2	nydk	Belongs to an aid and social services organization		X		
	p39l2_2	nydk	Took part in activities of an aid and social services organization		X		
	p39l3_2	nydk	Donated to an aid and social services organization		X		
	p39l4_2	nydk	Volunteered in an aid and social services organization		X		
	p39l9_2	nydk	DK/DA, an aid and social services organization		X		
SUB-BATTERY: belonging to and participation in any other organization							
p39m battery	p39m0_2	nydk	Does not belong to any other organization		X		
	p39m1_2	nydk	Belongs to any other organization		X		
	p39m2_2	nydk	Took part in activities of any other organization		X		
	p39m3_2	nydk	Donated to any other organization		X		
	p39m4_2	nydk	Volunteered in any other organization		X		
	p39m9_2	nydk	DK/DA, any other organization		X		
	p39mV_2		Other organizations, which ones		X		
BATTERY: knowledge of national party leaders							
p40 battery	p40a_2	yndk	Knows Pablo Casado		X	X	X
	p40b_2	yndk	Knows Pedro Sánchez		X	X	X
	p40c_2	yndk	Knows Albert Rivera		X	X	X
	p40d_2	yndk	Knows Pablo Iglesias		X	X	X
	p40e_2	yndk	Knows Alberto Garzón		X	X	X
	p40f_2	yndk	Knows Íñigo Urkullu		X	X	X
	p40g_2	yndk	Knows Carles Puigdemont		X	X	X
	p40h_2	yndk	Knows Oriol Junqueras		X	X	X
	p40i_4	yndk	Knows Santiago Abascal				X
BATTERY: rating of national party leaders							
p41 battery	p41a_2	dkda	Rate the political activity of Pablo Casado		X	X	X

Battery	Variable name	Value label	Variable label	W1	W2	W3	W4
	p41b_2	dkda	Rate the political activity of Pedro Sánchez		X	X	X
	p41c_2	dkda	Rate the political activity of Albert Rivera		X	X	X
	p41d_2	dkda	Rate the political activity of Pablo Iglesias		X	X	X
	p41e_2	dkda	Rate the political activity of Alberto Garzón		X	X	X
	p41f_2	dkda	Rate the political activity of Íñigo Urkullu		X	X	X
	p41g_2	dkda	Rate the political activity of Carles Puigdemont		X	X	X
	p41h_2	dkda	Rate the political activity of Oriol Junqueras		X	X	X
	p41i_4	dkda	Rate the political activity of Santiago Abascal				X
	p47_3		Satisfaction with the political situation in Spain			X	X
	p48_3	p48	Interest in the political campaign			X	X
	p49_3	p49	Newspapers as source of political and electoral information			X	X
	p50_3	p50	Tv as source of political and electoral information			X	X
	p51_3	p51	Radio as source of political and electoral information			X	X
	p52_3	p52	Internet as source of political and electoral information			X	X
BATTERY: Whether respondent consults electoral and political information in different websites							
p53 battery	p53a_3	nydk	Electoral and political information from media websites			X	X
	p53b_3	nydk	Electoral and political information from parties or candidates' websites			X	X
	p53c_3	nydk	Electoral and political information from civil organizations websites			X	X
	p53d_3	nydk	Electoral and political information from blogs			X	X
	p53e_3	nydk	Electoral and political information from social networks			X	X
	p53f_3	nydk	Electoral and political information from another website			X	X
	p53fV1_3		Electoral and political information, which other site			X	X
	p53g_3	nydk	Electoral and political information from unknown website			X	X
	p53h_3	nydk	Question about website does not apply			X	X
BATTERY: Personality traits attributed by the respondent to Pablo Casado							
p54 battery	p54a_3	agree5i	Pablo Casado is decided			X	X
	p54b_3	agree5i	Pablo Casado is intelligent			X	X

Battery	Variable name	Value label	Variable label	W1	W2	W3	W4
	p54c_3	agree5i	Pablo Casado is charismatic			X	X
	p54d_3	agree5i	Pablo Casado is incompetent			X	X
	p54e_3	agree5i	Pablo Casado is understanding			X	X
	p54f_3	agree5i	Pablo Casado is trustworthy			X	X
	p54g_3	agree5i	Pablo Casado is dishonest			X	X
	p54h_3	agree5i	Pablo Casado is arrogant			X	X
	p55_3	p55	Similar qualities to those of Pablo Casado			X	X
	p56_3	p56	Admires the qualities of Pablo Casado			X	X
BATTERY: Identification with Pablo Casado							
p57 battery	p57a_3	agree5i	Feels identified with supporters of Pablo Casado			X	X
	p57b_3	agree5i	Feels identified with Pablo Casado			X	X
	p57c_3	agree5i	Shares beliefs with Pablo Casado			X	X
	p57d_3	agree5i	I have no problem admitting I voted for Pablo Casado			X	X
	p57e_3	agree5i	The ideals of his party are represented by Pablo Casado			X	X
BATTERY: Personality traits attributed by the respondent to Pedro Sánchez							
p58 battery	p58a_3	agree5i	Pedro Sánchez is decided			X	X
	p58b_3	agree5i	Pedro Sánchez is intelligent			X	X
	p58c_3	agree5i	Pedro Sánchez is charismatic			X	X
	p58d_3	agree5i	Pedro Sánchez is incompetent			X	X
	p58e_3	agree5i	Pedro Sánchez is understanding			X	X
	p58f_3	agree5i	Pedro Sánchez is trustworthy			X	X
	p58g_3	agree5i	Pedro Sánchez is dishonest			X	X
	p58h_3	agree5i	Pedro Sánchez is arrogant			X	X
	p59_3	p59	Similar qualities to those of Pedro Sánchez			X	X
	p60_3	p60	Admires the qualities of Pedro Sánchez			X	X
BATTERY: Identification with Pedro Sánchez							
p61 battery	p61a_3	agree5i	Feels identified with supporters of Pedro Sánchez			X	X
	p61b_3	agree5i	Feels identified with Pedro Sánchez			X	X
	p61c_3	agree5i	Shares beliefs with Pedro Sánchez			X	X

Battery	Variable name	Value label	Variable label	W1	W2	W3	W4
	p61d_3	agree5i	I have no problem admitting I voted for Pedro Sánchez			X	X
	p61e_3	agree5i	The ideals of his party are represented by Pedro Sánchez			X	X
BATTERY: Personality traits attributed by the respondent to Albert Rivera							
62 battery	p62a_3	agree5i	Albert Rivera is decided			X	X
	p62b_3	agree5i	Albert Rivera is intelligent			X	X
	p62c_3	agree5i	Albert Rivera is charismatic			X	X
	p62d_3	agree5i	Albert Rivera is incompetent			X	X
	p62e_3	agree5i	Albert Rivera is understanding			X	X
	p62f_3	agree5i	Albert Rivera is trustworthy			X	X
	p62g_3	agree5i	Albert Rivera is dishonest			X	X
	p62h_3	agree5i	Albert Rivera is arrogant			X	X
	p63_3	p63	Similar qualities to those of Albert Rivera			X	X
	p64_3	p64	Admires the qualities of Albert Rivera			X	X
BATTERY: Identification with Albert Rivera							
p65 battery	p65a_3	agree5i	Feels identified with supporters of Albert Rivera			X	X
	p65b_3	agree5i	Feels identified with Albert Rivera			X	X
	p65c_3	agree5i	Shares beliefs with Albert Rivera			X	X
	p65d_3	agree5i	I have no problem admitting I voted for Albert Rivera			X	X
	p65e_3	agree5i	The ideals of his party are represented by Albert Rivera			X	X
BATTERY: Personality traits attributed by the respondent to Pablo Casado							
p66 battery	p66a_3	agree5i	Pablo Iglesias is decided			X	X
	p66b_3	agree5i	Pablo Iglesias is intelligent			X	X
	p66c_3	agree5i	Pablo Iglesias is charismatic			X	X
	p66d_3	agree5i	Pablo Iglesias is incompetent			X	X
	p66e_3	agree5i	Pablo Iglesias is understanding			X	X
	p66f_3	agree5i	Pablo Iglesias is trustworthy			X	X
	p66g_3	agree5i	Pablo Iglesias is dishonest			X	X
	p66h_3	agree5i	Pablo Iglesias is arrogant			X	X
	p67_3	p67	Similar qualities to those of Pablo Iglesias			X	X

Battery	Variable name	Value label	Variable label	W1	W2	W3	W4
	p68_3	p68	Admires the qualities of Pablo Iglesias			X	X
BATTERY: Identification with Pablo Iglesias							
p69 battery	p69a_3	agree5i	Feels identified with supporters of Pablo Iglesias			X	X
	p69b_3	agree5i	Feels identified with Pablo Iglesias			X	X
	p69c_3	agree5i	Shares beliefs with Pablo Iglesias			X	X
	p69d_3	agree5i	I have no problem admitting I voted for Pablo Iglesias			X	X
	p69e_3	agree5i	The ideals of his party are represented by Pablo Iglesias			X	X
BATTERY: Frequency with which s/he view political information in the social networks of different people							
p70 battery	p70a_3	p70a	Political information from the social networks of family and friends			X	X
	p70b_3	p70b	Political information from the social networks of parties and candidates			X	X
	p70c_3	p70c	Political information from the social networks of media			X	X
	p70d_3	p70d	Political information from the social networks of journalists			X	X
	p70e_3	p70e	Political information from the social networks of famous people			X	X
BATTERY: Trust in the information shared on social networks by different people							
p71 battery	p71a_3	p71a	Trust in the information shared on social networks by family and friends			X	X
	p71b_3	p71b	Trust in the information shared on social networks by parties and candidates			X	X
	p71c_3	p71c	Trust in the information shared on social networks by media			X	X
	p71d_3	p71d	Trust in the information shared on social networks by journalists			X	X
	p71e_3	p71e	Trust in the information shared on social networks by famous people			X	X
BATTERY: Opinion about each party							
p72 battery	p72a_3	p72a	Opinion about PP			X	X
	p72b_3	p72b	Opinion about PSOE			X	X
	p72c_3	p72c	Opinion about UP			X	X
	p72e_3	p72e	Opinion about Cs			X	X
	p72f_3	p72f	Opinion about ERC			X	X
	p72g_3	p72g	Opinion about JxCAT			X	X
	p72h_3	p72h	Opinion about EAJ-PNV			X	X
	p72i_3	p72i	Opinion about EH-Bildu			X	X

Battery	Variable name	Value label	Variable label	W1	W2	W3	W4
	p72l_3	p72l	Opinion about VOX			X	X
	p72m_3	p72m	Opinion about Compromís			X	X
	p72n_3	p72n	Opinion about CC			X	X
	p72o_3	p72o	Opinion about En Marea			X	X
	p73_3	dkda	Probability to vote on April 28th National Elections / on May 26th Elections			X	X
BATTERY: Probability of ever voting for each party							
p74 battery	p74a_3	dkda	Probability of ever voting PP			X	X
	p74b_3	dkda	Probability of ever voting PSOE			X	X
	p74c_3	dkda	Probability of ever voting UP			X	X
	p74e_3	dkda	Probability of ever voting Cs			X	X
	p74f_3	dkda	Probability of ever voting ERC			X	X
	p74g_3	dkda	Probability of ever voting JxCAT			X	X
	p74h_3	dkda	Probability of ever voting EAJ-PNV			X	X
	p74i_3	dkda	Probability of ever voting EH-Bildu			X	X
	p74l_3	dkda	Probability of ever voting VOX			X	X
	p74m_3	dkda	Probability of ever voting Compromís			X	X
	p74n_3	dkda	Probability of ever voting CC			X	X
	p74o_3	dkda	Probability of ever voting En Marea			X	X
	p74p_3	dkda	Probability of ever voting FAC			X	X
	p74q_3	dkda	Probability of ever voting PRC			X	X
	p75_3	p75	Vote intention for the general elections of April 28th			X	
	p76_4	p76	Satisfaction with democracy in the EU				X
	p77_4	p77	Spain as part of the EU, opinion				X
	p78_4	dkda	Opinion on EU Integration				X
BATTERY: Position attributed to each party regarding EU integration							
p79 battery	p79a_4	dkda	PP position on EU integration				X
	p79b_4	dkda	PSOE position on EU integration				X
	p79c_4	dkda	UP position on EU integration				X
	p79e_4	dkda	Cs position on EU integration				X
	p79f_4	dkda	ERC position on EU integration				X

Battery	Variable name	Value label	Variable label	W1	W2	W3	W4
	p79g_4	dkda	JxCAT position on EU integration				X
	p79h_4	dkda	EAJ-PNV position on EU integration				X
	p79i_4	dkda	EH-Bildu position on EU integration				X
	p79l_4	dkda	VOX position on EU integration				X
	p79m_4	dkda	Compromís position on EU integration				X
	p79n_4	dkda	CC position on EU integration				X
	p79o_4	dkda	En Marea position on EU integration				X
	p80_4	p80	Vote intention for the May 26th EP Elections				X
	p80V11_4		Vote intention for the May 26th EP Elections, which other				X
	p81_4	p81	Vote intention for the May 26th Regional Elections				X
	p81V24_4		Vote intention for the May 26th Regional Elections, which other				X

Trust in Political Institutions Non-Experimental Variables

Table 11 shows the list of trust in political institutions for the non-experimental variables. They consist of a single battery in the second, third and fourth waves, including ten questions per wave to capture how much do respondents trust a series of ten political institutions.

Table 11 List of Trust in Political Institutions Non-Experimental Variables

Variable name	Value label	Variable label	W1	W2	W3	W4
trust1a_2	dkda	Trust in the Spanish Parliament		x	x	x
trust1b_2	dkda	Trust in the Spanish Government		x	x	x
trust1c_2	dkda	Trust in the Regional Parliament		x	x	x
trust1d_2	dkda	Trust in the Regional Government		x	x	x
trust1e_2	dkda	Trust in Spanish politicians		x	x	x
trust1f_2	dkda	Trust in Spanish political parties		x	x	x
trust1g_2	dkda	Trust in the Spanish Police		x	x	x
trust1h_2	dkda	Trust in the Spanish Judiciary		x	x	x
trust1i_2	dkda	Trust in the European Parliament		x	x	x
trust1j_2	dkda	Trust in the European Commission		x	x	x

First Experiment Variables (Trust in Political Institutions)

Table 12 shows the experimental variables of EXPERIMENT 1, carried out in the first wave. Remember that, in this experiment,

- The control group (CG) was shown the full grid of institutions.
- The first treatment group (TR1) was shown no grid of institutions, as these were shown sequentially instead.
- The second treatment group (TR2) was shown separate grids of institutions.
- The third treatment group (TR3) received a previous framing by means of an article on parliament.
- The fourth treatment group (TR4) received a previous framing by means of an article on politicians.
- And the fifth treatment group (TR5) received a previous framing by means of an article on the judiciary.

Table 12 List of Variables for the First Experiment (Trust in Political Institutions)

Variable name	Value label	Variable label	W1
et0_1	et0	Group in EXPERIMENT 1 (trust in political institutions)	X
et1a_1	dkda	Trust in the Spanish Parliament (CG)	X
et1b_1	dkda	Trust in the Spanish Government (CG)	X
et1c_1	dkda	Trust in the Regional Parliament (CG)	X
et1d_1	dkda	Trust in the Regional Government (CG)	X
et1e_1	dkda	Trust in Spanish politicians (CG)	X
et1f_1	dkda	Trust in Spanish political parties (CG)	X
et1g_1	dkda	Trust in the Spanish Police (CG)	X
et1h_1	dkda	Trust in the Spanish Judiciary (CG)	X
et1i_1	dkda	Trust in the European Parliament (CG)	X
et1j_1	dkda	Trust in the European Commission (CG)	X
et2a_1	dkda	Trust in the Spanish Parliament (TR1)	X
et2b_1	dkda	Trust in the Spanish Government (TR1)	X
et2c_1	dkda	Trust in the Regional Parliament (TR1)	X
et2d_1	dkda	Trust in the Regional Government (TR1)	X

et2e_1	dkda	Trust in Spanish politicians (TR1)	X
et2f_1	dkda	Trust in Spanish political parties (TR1)	X
et2g_1	dkda	Trust in the Spanish Police (TR1)	X
et2h_1	dkda	Trust in the Spanish Judiciary (TR1)	X
et2i_1	dkda	Trust in the European Parliament (TR1)	X
et2j_1	dkda	Trust in the European Commission (TR1)	X
et3a_1	dkda	Trust in the Spanish Parliament (TR2)	X
et3b_1	dkda	Trust in the Spanish Government (TR2)	X
et3c_1	dkda	Trust in the Regional Parliament (TR2)	X
et3d_1	dkda	Trust in the Regional Government (TR2)	X
et3e_1	dkda	Trust in Spanish politicians (TR2)	X
et3f_1	dkda	Trust in Spanish political parties (TR2)	X
et3g_1	dkda	Trust in the Spanish Police (TR2)	X
et3h_1	dkda	Trust in the Spanish Judiciary (TR2)	X
et3i_1	dkda	Trust in the European Parliament (TR2)	X
et3j_1	dkda	Trust in the European Commission (TR2)	X
et4a_1	dkda	Trust in the Spanish Parliament (TR3)	X
et4b_1	dkda	Trust in the Spanish Government (TR3)	X
et4c_1	dkda	Trust in the Regional Parliament (TR3)	X
et4d_1	dkda	Trust in the Regional Government (TR3)	X
et4e_1	dkda	Trust in Spanish politicians (TR3)	X
et4f_1	dkda	Trust in Spanish political parties (TR3)	X
et4g_1	dkda	Trust in the Spanish Police (TR3)	X
et4h_1	dkda	Trust in the Spanish Judiciary (TR3)	X
et4i_1	dkda	Trust in the European Parliament (TR3)	X
et4j_1	dkda	Trust in the European Commission (TR3)	X
et4P1_1	dkda	Positive tone/sentiment of parliamentary news' content (TR3)	X
et4P2_1	et4P2	Institutions alluded in parliamentary news (TR3)	X
et4P3_1	dkda	Importance of parliamentary news' topic for democracy (TR3)	X
et5a_1	dkda	Trust in the Spanish Parliament (TR4)	X
et5b_1	dkda	Trust in the Spanish Government (TR4)	X
et5c_1	dkda	Trust in the Regional Parliament (TR4)	X

et5d_1	dkda	Trust in the Regional Government (TR4)	X
et5e_1	dkda	Trust in Spanish politicians (TR4)	X
et5f_1	dkda	Trust in Spanish political parties (TR4)	X
et5g_1	dkda	Trust in the Spanish Police (TR4)	X
et5h_1	dkda	Trust in the Spanish Judiciary (TR4)	X
et5i_1	dkda	Trust in the European Parliament (TR4)	X
et5j_1	dkda	Trust in the European Commission (TR4)	X
et5P1_1	dkda	Positive tone/sentiment of politicians’ news content (TR4)	X
et5P2_1	et5P2	Institutions alluded in politicians’ news (TR4)	X
et5P3_1	dkda	Importance of politicians’ news topic for democracy (TR4)	X
et6a_1	dkda	Trust in the Spanish Parliament (TR5)	X
et6b_1	dkda	Trust in the Spanish Government (TR5)	X
et6c_1	dkda	Trust in the Regional Parliament (TR5)	X
et6d_1	dkda	Trust in the Regional Government (TR5)	X
et6e_1	dkda	Trust in Spanish politicians (TR5)	X
et6f_1	dkda	Trust in Spanish political parties (TR5)	X
et6g_1	dkda	Trust in the Spanish Police (TR5)	X
et6h_1	dkda	Trust in the Spanish Judiciary (TR5)	X
et6i_1	dkda	Trust in the European Parliament (TR5)	X
et6j_1	dkda	Trust in the European Commission (TR5)	X
et6P1_1	dkda	Positive tone/sentiment of judicial news' content (TR5)	X
et6P2_1	et6P2	Institutions alluded in judicial news (TR5)	X
et6P3_1	dkda	Importance of judicial news' topic for democracy (TR5)	X

Second Experiment Variables (Traditional Media and Framing)

Table 13 shows eight experimental variables (the last four of them, post-experimental ones) of experiment 2 (EXP 2), all of which corresponding to the second wave of the survey.

Table 13 List of Variables for the Second Experiment (Traditional Media and Framing)

Variable name	Value label	Variable label	W2
em0_2	em0	Group in EXPERIMENT 2	X
em0a_2		News' start day (EXP 2)	X
em0b_2		News' end day (EXP 2)	X
em0c_2		Exposure: news' duration in seconds (EXP 2)	X
emP1_2	emP1	Journal chosen or assigned (EXP 2)	X
emP2_2	emP2	News' topic (EXP 2)	X
emP3_2	emP3	News' negative tone/sentiment (EXP 2)	X
emP4_2	emP4	The news helped position on the subject (EXP 2)	X

Third Experiment Variables (Social Media in National Elections)

Table 14 shows the variables in the third experiment (EXP 3), all of them corresponding to the third wave of the survey. This experiment aimed at capturing the effect of exposure to social media during National Elections.

Table 14 List of Variables for the Third Experiment (Social Media during National Elections)

Variable name	Value label	Variable label	W3
esm0_3	esm0	Group in EXPERIMENT 3	X
esm0a_3	yndk	Willingness to participate (EXP 3)	X
esmP1_3	yndk	Finally did EXPERIMENT 3	X
esmP2a_3	nydk	Followed the account of Pablo Casado (EXP 3)	X
esmP2b_3	nydk	Followed the account of Pedro Sánchez (EXP 3)	X
esmP2c_3	nydk	Followed the account of Pablo Iglesias (EXP 3)	X
esmP2d_3	nydk	Followed the account of Albert Rivera (EXP 3)	X
esmP2e_3	nydk	Followed the account of Santiago Abascal (EXP 3)	X
esmP2f_3	nydk	Followed the account of Carles Puigdemont (EXP 3)	X
esmP3_3	esmP3	Followed the accounts for EXPERIMENT 3 or before	X
esmP3a_3	nydk	Started following the account of Pablo Casado (EXP 3)	X
esmP3b_3	nydk	Started following the account of Pedro Sánchez (EXP 3)	X
esmP3c_3	nydk	Started following the account of Pablo Iglesias (EXP 3)	X
esmP3d_3	nydk	Started following the account of Albert Rivera (EXP 3)	X
esmP3e_3	nydk	Started following the account of Santiago Abascal (EXP 3)	X
esmP3f_3	nydk	Started following the account of Carles Puigdemont (EXP 3)	X
esmP4a_3	esmP4a	How frequently did Pablo Casado tweet (EXP 3)	X
esmP4b_3	esmP4b	How frequently did Pedro Sánchez tweet (EXP 3)	X
esmP4c_3	esmP4c	How frequently did Pablo Iglesias tweet (EXP 3)	X
esmP4d_3	esmP4d	How frequently did Albert Rivera tweet (EXP 3)	X
esmP4e_3	esmP4e	How frequently did Santiago Abascal tweet (EXP 3)	X
esmP4f_3	esmP4f	How frequently did Carles Puigdemont tweet (EXP 3)	X
esmP5a1_3	nydk	Pablo Casado mainly used text (EXP 3)	X
esmP5a2_3	nydk	Pablo Casado mainly used images (EXP 3)	X
esmP5a3_3	nydk	Pablo Casado mainly used videos (EXP 3)	X
esmP5a4_3	nydk	Pablo Casado mainly used links to news (EXP 3)	X

esmP5a8_3	nydk	Pablo Casado mainly used [dk] (EXP 3)	X
esmP5b1_3	nydk	Pedro Sánchez mainly used text (EXP 3)	X
esmP5b2_3	nydk	Pedro Sánchez mainly used images (EXP 3)	X
esmP5b3_3	nydk	Pedro Sánchez mainly used videos (EXP 3)	X
esmP5b4_3	nydk	Pedro Sánchez mainly used links to news (EXP 3)	X
esmP5b8_3	nydk	Pedro Sánchez mainly used [dk] (EXP 3)	X
esmP5c1_3	nydk	Pablo Iglesias mainly used text (EXP 3)	X
esmP5c2_3	nydk	Pablo Iglesias mainly used images (EXP 3)	X
esmP5c3_3	nydk	Pablo Iglesias mainly used videos (EXP 3)	X
esmP5c4_3	nydk	Pablo Iglesias mainly used links to news (EXP 3)	X
esmP5c8_3	nydk	Pablo Iglesias mainly used [dk] (EXP 3)	X
esmP5d1_3	nydk	Albert Rivera mainly used text (EXP 3)	X
esmP5d2_3	nydk	Albert Rivera mainly used images (EXP 3)	X
esmP5d3_3	nydk	Albert Rivera mainly used videos (EXP 3)	X
esmP5d4_3	nydk	Albert Rivera mainly used links to news (EXP 3)	X
esmP5d8_3	nydk	Albert Rivera mainly used [dk] (EXP 3)	X
esmP5e1_3	nydk	Santiago Abascal mainly used text (EXP 3)	X
esmP5e2_3	nydk	Santiago Abascal mainly used images (EXP 3)	X
esmP5e3_3	nydk	Santiago Abascal mainly used videos (EXP 3)	X
esmP5e4_3	nydk	Santiago Abascal mainly used links to news (EXP 3)	X
esmP5e8_3	nydk	Santiago Abascal mainly used [dk] (EXP 3)	X
esmP5f1_3	nydk	Carles Puigdemont mainly used text (EXP 3)	X
esmP5f2_3	nydk	Carles Puigdemont mainly used images (EXP 3)	X
esmP5f3_3	nydk	Carles Puigdemont mainly used videos (EXP 3)	X
esmP5f4_3	nydk	Carles Puigdemont mainly used links to news (EXP 3)	X
esmP5f8_3	nydk	Carles Puigdemont mainly used [dk] (EXP 3)	X
esmP6a_3	esmP6a	Hashtag associated to Pablo Casado (EXP 3)	X
esmP6b_3	esmP6b	Hashtag associated to Pedro Sánchez (EXP 3)	X
esmP6c_3	esmP6c	Hashtag associated to Pablo Iglesias (EXP 3)	X
esmP6d_3	esmP6d	Hashtag associated to Albert Rivera (EXP 3)	X
esmP6e_3	esmP6e	Hashtag associated to Santiago Abascal (EXP 3)	X
esmP6f_3	esmP6f	Hashtag associated to Carles Puigdemont (EXP 3)	X

Fourth Experiment Variables (Social Media in European Elections)

Table 15 shows the variables in the fourth experiment (EXP 4), all of them corresponding to the fourth wave of the survey. This experiment aimed at capturing the effect of exposure to social media in European elections.

Table 15 List of Variables for the Fourth Experiment (Social Media in European elections)

Variable name	Value label	Variable label	W4
esm0_4	esm0	Group in EXPERIMENT 4	X
esm0a_4	yndk	Willingness to participate (EXP 4)	X
esmP1_4	yndk	Finally did the experiment (EXP 4)	X
esmP7_4	esmP7	Account followed (in EXP 4)	X
esmP8_4	esmP8	Followed the account for the experiment (EXP 4) or before	X
esmP9_4	esmP9	Topics discussed in the account (EXP 4)	X
esmP10_4	agree5i	Agreement with the opinions in the account (EXP 4)	X
esmP11_4	esmP11	Tone of the discussions in the account (EXP 4)	X
esmP12_4	esmP12	Trustworthiness of the accounts' information (EXP 4)	X

7. Codes for Categorical Variables

Below, we show the correspondence between the coding and labels of each of the variables having a non-generic label (we also display the coding of some categorical variables with generic value labels). When several consecutive variables (most often, of the same battery) have the same coding, after showing the names of all the variables, their coding is shown only once:

Global and Wave-Specific Categorical Variables

No global variables have specific value labels. Four of them, though, have generic value labels. As to wave-specific variables, only “w4” (in the four waves) is categorical. The coding of these variables is as follows:

g2_0 (Completed wave 2 (nested in wave 1)):
g3_0 (Completed wave 3 (nested in waves 2 & 1)):
g4_0 (Completed wave 4 (nested only in wave 1)):
g4a_0 (Completed all waves (w3 nested in w2 & w1)):
Minimum: 0. Maximum: 1
0 = No
1 = Yes

w4_1 (Device used for the interview):
Minimum: 1. Maximum: 3
1 = Desktop
2 = Tablet
3 = Mobile

Socio-Demographic Categorical Variables

s0_1 (Region):
Minimum: 1. Maximum: 17
1 = Andalucía
2 = Aragón
3 = Asturias
4 = Islas Baleares
5 = Canarias
6 = Cantabria
7 = Castilla y León
8 = Castilla-La Mancha
9 = Cataluña
10 = Comunidad Valenciana

11 = Extremadura
12 = Galicia
13 = Madrid
14 = Murcia
15 = Navarra
16 = País Vasco
17 = La Rioja

s1_1 (Sex):
Minimum: 1. Maximum: 2
1 = Man
2 = Woman

s2R_1 (Age group):
Minimum: 2. Maximum: 6
2 = 18_24
3 = 25_34
4 = 35_44
5 = 45_54
6 = 55_+

s3a_1 (Habitat (number of inhabitants)):
Minimum: 1. Maximum: 3
1 = Less than 50,000
2 = Between 50,000 and 200,000
3 = More than 200,000

s3b_1 (Place of residence):
Minimum: 1. Maximum: 5
1 = Big city
2 = Suburb City
3 = Medium sized town
4 = Small town
5 = Rural area or village

s4a_1 (Education, 8 levels):
Minimum: 1. Maximum: 8
1 = Without education
2 = Primary education
3 = Lower secondary education
4 = Upper secondary education
5 = Bachelor's, 3 years
6 = Bachelor's, 5 years
7 = Master's
8 = Doctorate

s4b_1 (Education, 28 categories):

Minimum: 1. Maximum: 27

1 = Less than 5 years of School(uncomplete primary education)
2 = Certified Former Primary Education
3 = 5th Grade of Basic Primary Education
4 = Primary Education (LOGSE)
5 = Elementary Degree in Music and Dance
6 = Elementary High School
7 = Basic Primary Education(EGB)
8 = Compulsory Secondary Education (ESO)
9 = Senior High School(BUP)
10 = Pre-University Course (PREU,COU)
11 = High School Degree (LOGSE)
12 = Initial Professional Training
13 = Social Guarantee Programmes or Initial Professional Qualification Programme
14 = Officer Professional Training
15 = First-Level Professional Training (FPI)
16 = Intermediate Technical Training Course
17 = Intermediate Training Course in Plastic Arts and Design
18 = Intermediate Degree in Music and Dance
19 = Master Professional Training
20 = Second-Level Professional Training(FPII)
21 = Advanced Technical Training Course
22 = Advanced Degree Training Course in Art
23 = Expert, former nursing, teaching or social work schools
24 = Diploma, Degree (Bologna), Engineering or Technical Architecture,3 years undergraduate
25 = Bachelor of Arts, Master's Degree (Bologna), Higher Engineering, Architecture, Higher Degree
26 = Doctorate
27 = Others

s5_1 (Marital status):

Minimum: 1. Maximum: 6

1 = Married
2 = Civil partner
3 = Legally Separated
4 = Divorced
5 = Widowed
6 = Single

s8_1 (Occupation):

Minimum: 1. Maximum: 8

1 = Salaried employee
2 = Self-employed
3 = Student
4 = Unemployed, searching for a job

5 = Unemployed, not searching
6 = Disabled
7 = Retired
8 = Housework

s9_1 (Subjective wellbeing):
Minimum: 1. Maximum: 4
1 = Living comfortably on present income
2 = Coping on present income
3 = Finding it difficult on present income
4 = Finding it very difficult on present income

s10_1 (Lost job in last year):
Minimum: 1. Maximum: 2
1 = Yes
2 = No

s11a_1 (Concern about bills):
Minimum: 0. Maximum: 3
0 = Not at all concerned
1 = Barely concerned
2 = Quite concerned
3 = Very much concerned

s11b_1 (Concern about reducing lifestyle):
Minimum: 0. Maximum: 3
0 = Not at all concerned
1 = Barely concerned
2 = Quite concerned
3 = Very much concerned

s11c_1 (Concern about getting a job):
Minimum: 0. Maximum: 3
0 = Not at all concerned
1 = Barely concerned
2 = Quite concerned
3 = Very much concerned

s11d_1 (Concern about loans and mortgages):
Minimum: 0. Maximum: 3
0 = Not at all concerned
1 = Barely concerned
2 = Quite concerned
3 = Very much concerned

s12_1 (Income):

Minimum: 1. Maximum: 10

1 = 780 € or less
2 = More than 780 € to 1000 €
3 = More than 1001 € to 1250 €
4 = More than 1251 € to 1500 €
5 = More than 1501 € to 1800 €
6 = More than 1801 € to 2200 €
7 = More than 2201 € to 2500 €
8 = More than 2501 € to 2850 €
9 = More than 2851 € to 3700 €
10 = More than 3701 €

s12a_1 (Expresses income in weeks, months, or years):

Minimum: 1. Maximum: 3

1 = Weekly
2 = Monthly
3 = Yearly

s14_1 (Belongs to a religion):

Minimum: 0. Maximum: 1

0 = No
1 = Yes

s14a_1 (Religious Denomination):

Minimum: 1. Maximum: 8

1 = Roman Catholic
2 = Protestant
3 = Eastern Orthodox
4 = Other Christian denomination
5 = Jewish
6 = Islamic
7 = Eastern Religions
8 = Other non-Christian religions
. = .

s14b_1 (Church attendance):

Minimum: 0. Maximum: 6

0 = Never
1 = Only occasionally
2 = Only on special holidays
3 = At least once a month
4 = Once a week
5 = More than once a week
6 = Every day

Opinion or Attitudinal Categorical Variables

There are many opinion and attitudinal variables ("p" variables) that are categorical, often with non-generic value labels. We show them below. Incidentally, it is worthwhile to mention that all non-experimental trust-in-political-institutions categorical variables range from 1 (No trust at all) to 7 (Complete trust), and none of them is labelled.

p1_1 (Political Interest):
Minimum: 1. Maximum: 4
1 = Very interested
2 = Quite interested
3 = Hardly interested
4 = Not at all interested
.a = [DK]

p3_1 (Spain's main problem):
Minimum: 1. Maximum: 19
1 = Unemployment
2 = Drugs
3 = Health services
4 = Housing
5 = Education
6 = National Terrorism (ETA)
7 = International Terrorism (ISIS)
8 = Corruption
9 = Immigration
10 = Euro
11 = Violence against women
12 = Political Instability
13 = Refugee crisis
14 = Environmental problems
15 = Pensions
16 = Public insecurity
17 = Taxes
18 = Political Parties and Politicians
19 = Others
.a = [DK]

p17a_1 (Use of newspapers for political information):
p17b_1 (Use of radio for political information):
p17c_1 (Use of magazines for political information):
p17d_1 (Use of Tv for political information):
p17e_1 (Use of social networks for political information):
Minimum: 0. Maximum: 8
0 = Never
1 = Less than monthly

2 = Monthly
3 = Several times per month
4 = Weekly
5 = Several times per week
7 = Daily
8 = Several times per day
.a = [DK]

p18_1 (Use of internet as source of information):
Minimum: 0. Maximum: 7
0 = Never
1 = Less than monthly
2 = Monthly
3 = Several times per month
4 = Weekly
5 = Several times per week
6 = Daily
7 = Several times per day
.a = [DK]
.c = [NA]

p20a_1 (Twitter frequency):
p20b_1 (Facebook frequency):
p20c_1 (Google+ frequency):
p20d_1 (Linkedin frequency):
p20e_1 (Instagram frequency):
p20f_1 (Flickr frequency):
p20g_1 (YouTube frequency):
p20h_1 (Another social network frequency):
Minimum: 0. Maximum: 7
0 = Never
1 = Less than monthly
2 = Monthly
3 = Several times per month
4 = Weekly
5 = Several times per week
6 = Daily
7 = Several times per day
. = .
.a = [DK]

p21_1 (Frequency of political news found on social networks):
Minimum: 0. Maximum: 4
0 = Never
1 = Sometimes

2 = Often
3 = Very often
4 = Always
.a = [DK]

p22a_1 (Internet and social networks help to increase participation in your groups):
p22b_1 (Internet and social networks help to increase participation in new groups):
p22c_1 (Internet and social networks help to relate with people with same interests):
p22d_1 (Internet and social networks help to relate with people with same religion):
p22e_1 (Internet and social networks help to relate with people with same political idea):
p22f_1 (Internet and social networks help to relate with people from different cultures):
p22g_1 (Internet and social networks help to relate with people of different ages):
p22h_1 (Internet and social networks help to relate with people from different countries):
Minimum: 0. Maximum: 3
0 = Not at all
1 = Very Little
2 = Some
3 = A lot
.a = [DK]

p25_1 (Political accounts followed on Twitter):
Minimum: 0. Maximum: 4
0 = Do not follow political parties or politicians
1 = Only of one political party
2 = Only of one candidate
3 = Of several candidates of the same party
4 = Of several parties and candidates
.a = [DK]

p26a_1 (Frequency of political discussions with family and friends):
p26b_1 (Frequency of political discussions on social networks):
Minimum: 0. Maximum: 6
0 = Never
1 = Less than monthly
2 = Monthly
3 = Several times per month
4 = Weekly
5 = Several times per week
6 = Daily
.a = [DK]

p27a_1 (Frequency of agreement when discussing about politics):
p27b_1 (Frequency of agreement when discussing about politics on social networks):
p28a_1 (Frequency of disagreement when discussing about politics):
p28b_1 (Frequency of disagreement when discussing about politics on social networks):
Minimum: 0. Maximum: 3

0 = Never
1 = Sometimes
2 = Often
3 = Always
.a = [DK]

p29a_1 (Political preferences of people I discuss politics with):
p29b_1 (Political preferences of people I discuss politics with on social networks):
Minimum: 0. Maximum: 3
0 = They do not support any party
1 = They support a different party
2 = They divide their support between different parties
3 = They support the same party
.a = [DK]

p30a_1 (Vote recall of the June 2016 National Elections):
Minimum: 1. Maximum: 11
1 = PP (Partido Popular)
2 = PSOE (Partido Socialista Obrero Español)
3 = UP (Podemos y listas locales y municipales-IU-Equo)
4 = Cs-Partido de la Ciudadanía (C's - Ciutadans)
5 = ERC (Esquerra Republicana de Catalunya)
6 = CDC (Convergencia Democrática de Catalunya)
7 = EAJ - PNV (Euzko Alderdi Jeltzalea - Partido Nacionalista Vasco)
8 = EH - Bildu (Euskal Herria - Bildu)
9 = BNG (Bloque Nacionalista Galego)
10 = CC (Coalición Canaria)
11 = Others
.a = [DK]

p31_1 (Vote intention if you had voted in the last National Elections):
Minimum: 1. Maximum: 11
1 = PP (Partido Popular)
2 = PSOE (Partido Socialista Obrero Español)
3 = UP (Podemos y listas locales y municipales-IU-Equo)
4 = Cs-Partido de la Ciudadanía (C's - Ciutadans)
5 = ERC (Esquerra Republicana de Catalunya)
6 = CDC (Convergencia Democrática de Catalunya)
7 = EAJ - PNV (Euzko Alderdi Jeltzalea - Partido Nacionalista Vasco)
8 = EH - Bildu (Euskal Herria - Bildu)
9 = BNG (Bloque Nacionalista Galego)
10 = CC (Coalición Canaria)
11 = Others
. = .
.a = [DK]

p35a_1 (Party id specification):

Minimum: 1. Maximum: 12

1 = PP (Partido Popular)
2 = PSOE (Partido Socialista Obrero Español)
3 = UP (En Comú Podem, En Marea, Ahora Madrid)
4 = IU (Izquierda Unida)
5 = Cs (C’s - Ciutadans)
6 = ERC (Esquerra Republicana de Catalunya)
7 = PDeCAT (Partit Demòcrata Europeu Català)
8 = EAJ - PNV (Euzko Alderdi Jeltzalea - Partido Nacionalista Vasco)
9 = EH - Bildu (Euskal Herria - Bildu)
10 = BNG (Bloque Nacionalista Galego)
11 = CC (Coalición Canaria)
12 = Others
.a = [DK]

p35b_1 (Degree of closeness to political party):

Minimum: 0. Maximum: 3

0 = Not at all close
1 = Not close
2 = Quite close
3 = Very close
.a = [DK]

p35c_1 (Importance of Party id):

Minimum: 0. Maximum: 3

0 = Not at all important
1 = Not very important
2 = Very important
3 = Extremely important
.a = [DK]

p35d_1 (How well does your Party id describe you):

Minimum: 0. Maximum: 3

0 = Not at all
1 = Not so well
2 = Very well
3 = Extremely well
.a = [DK]

p35e_1 (Use of We when referring the political party of your preference):

Minimum: 0. Maximum: 3

0 = Never
1 = Sometimes
2 = Most of the times
3 = Always

.a = [DK]

p35f_1 (Feels as part of the political party):
Minimum: 0. Maximum: 3
0 = Not at all
1 = Very little
2 = Somewhat
3 = A lot
.a = [DK]

p36a_1 (Margarita Robles is the Spanish Minister of Defence):
p36b_1 (The Spanish Congress has 525 members):
p36c_1 (To run in the Spanish National Elections you must be at least 25 years old):
p36d_1 (EU countries elect the same number of MPs to the EP):
p36e_1 (Norway is a member of the EU):
p36f_1 (Including Great Britain, there are 28 EU member countries):
Minimum: 1. Maximum: 2
1 = True
2 = False
.a = [DK]

p32a_2 (Signed a petition):
p32b_2 (Boycotted certain products):
p32c_2 (Displayed a campaign badge/sticker):
p32d_2 (Took part in a lawful public demonstration):
p32e_2 (Took part in a political rally):
p32f_2 (Contacted a politician or government official):
p32g_2 (Contacted or appeared in the media):
Minimum: 0. Maximum: 1
0 = No
1 = Yes
2 = [Does not remember]
.a = [DK]

p34a_2 (Started following people with different political opinions):
p34b_2 (Marked as liked a political comment or tweet):
p34c_2 (Shared political comments or tweets posted by others):
p34d_2 (Commented positively a political tweet or Fb post from others):
p34e_2 (Commented negatively a political tweet or Fb post from others):
p34f_2 (Unfollowed or blocked contacts for political reasons):
p34g_2 (Decided not to post political content to avoid offending others):
p34h_2 (Decided not to post political content to avoid public exposure):
p34i_2 (Changed your mind after taking part on a Fb or Tw political discussion):
p34j_2 (Increased political participation after a debate on social networks):
p34k_2 (Decreased political participation after a debate on social networks):
Minimum: 0. Maximum: 1

0 = No
1 = Yes
2 = Does not remember
.a = [DK]

p37a_2 (Spanish economic situation in the last 12 months):
p37b_2 (Household economic situation in the last 12 months):
Minimum: 1. Maximum: 5
1 = A lot worse
2 = A little worse
3 = It is the same
4 = A little better
5 = A lot better
. = .
.a = [DK]
.z = [NA: not in wave]

p42_2 (Shares twitter account):
Minimum: 1. Maximum: 1
1 = Yes, agrees to share account
.a = [DK]
.c = [NA]
.z = [NA: not in wave]

p48_3 (Interest in the political campaign):
Minimum: 1. Maximum: 4
1 = Not at all interested
2 = A little bit interested
3 = Quite interested
4 = Very interested
.a = [DK]
.z = [NA: not in wave]

p49_3 (Newspapers as source of political and electoral information):
p50_3 (Tv as source of political and electoral information):
p51_3 (Radio as source of political and electoral information):
p52_3 (Internet as source of political and electoral information):
Minimum: 1. Maximum: 5
1 = Daily or almost daily
2 = Several times per week
3 = Only on weekends
4 = Sometimes
5 = Never or hardly ever
.a = [DK]
.z = [NA: not in wave]

p55_3 (Similar qualities to those of Pablo Casado):
p56_3 (Admires the qualities of Pablo Casado):
p59_3 (Similar qualities to those of Pedro Sánchez):
p60_3 (Admires the qualities of Pedro Sánchez):
p63_3 (Similar qualities to those of Albert Rivera):
p64_3 (Admires the qualities of Albert Rivera):
p67_3 (Similar qualities to those of Pablo Iglesias):
p68_3 (Admires the qualities of Pablo Iglesias):
Minimum: 1. Maximum: 5
1 = A lot
2 = Quite a lot
3 = More or less
4 = Somewhat
5 = Not at all
. = .
.a = [DK]
.z = [NA: not in wave]

p70a_3 (Political information from the social networks of family and friends):
p70b_3 (Political information from the social networks of parties and candidates):
p70c_3 (Political information from the social networks of media):
p70d_3 (Political information from the social networks of journalists):
p70e_3 (Political information from the social networks of famous people):
Minimum: 1. Maximum: 777
1 = Daily or almost daily
2 = Several times per week
3 = Only on weekends
4 = Sometimes
5 = Never or hardly ever
777 = [Not re-contacted]
.a = [DK]
.c = [NA]
.z = [NA: not in wave]

p71a_3 (Trust in the information shared on social networks by family and friends):
p71b_3 (Trust in the information shared on social networks by parties and candidates):
p71c_3 (Trust in the information shared on social networks by media):
p71d_3 (Trust in the information shared on social networks by journalists):
p71e_3 (Trust in the information shared on social networks by famous people):
Minimum: 1. Maximum: 777
1 = Trusts completely
2 = Trusts a lot
3 = Trusts somewhat
4 = Does not trust very much
5 = Does not trust at all
777 = [Not re-contacted]

.a = [DK]
.z = [NA: not in wave]

p75_3 (Vote intention for the general elections of April 28th):
Minimum: 1. Maximum: 24
1 = PP
2 = PSOE
3 = UP
4 = En Comú Podem
5 = Cs
6 = ERC
7 = JxCat
8 = PNV-EAJ
9 = EH-Bildu
11 = CC
12 = Other
13 = VOX
14 = Compromís
15 = En Marea
20 = Blank vote
21 = Will not vote
22 = No right to vote
23 = Does not know
24 = Will not tell
.z = [NA: not in wave]

p76_4 (Satisfaction with democracy in the EU):
Minimum: 1. Maximum: 4
1 = Extremely satisfied
2 = Very satisfied
3 = Not very satisfied
4 = Not at all satisfied
. = .
.a = [DK]
.b = [DA]
.z = [NA: not in wave]

p77_4 (Spain as part of the EU, opinion):
Minimum: 1. Maximum: 3
1 = It is a good thing
2 = It is a bad thing
3 = None of the above
. = .
.a = [DK]
.b = [DA]
.z = [NA: not in wave]

p80_4 (Vote intention for the May 26th EP Elections):

Minimum: 1. Maximum: 15

1 = PSOE
2 = PP
3 = Cs
4 = UP
5 = VOX
6 = PACMA
7 = Ahora Repúblicas
8 = CEUS
9 = Junts
10 = Compromís per Europa
11 = Other
12 = Void vote
13 = Blank vote
14 = Will not vote
15 = I have not decided yet
. = .
.a = [DK]
.c = [NA]
.z = [NA: not in wave]

p81_4 (Vote intention for the May 26th Regional Elections):

Minimum: 1. Maximum: 28

1 = PSOE
2 = PP
3 = Cs
4 = UP
5 = Podemos
6 = IU
8 = VOX
9 = PACMA
10 = NA+
11 = GEBAI
12 = EH-Bildu
13 = CC
14 = NCa
15 = FAC
16 = CHA
17 = PAR
19 = PSM
20 = PRC
23 = Más Madrid
24 = Other
25 = Void vote

26 = Blank vote
27 = Will not vote
28 = Has not decided yet
. = .
.a = [DK]
.b = [DA]
.z = [NA: not in wave]

Experimental Categorical Variables

et0_1 (Group in EXPERIMENT 1 (trust in political institutions)):
Minimum: 1. Maximum: 6
1 = Control group, full institutional grid
2 = Treatment 1, institutions sequentially
3 = Treatment 2, institutions separate grids
4 = Treatment 3, framing, parliamentary news
5 = Treatment 4, framing, politicians news
6 = Treatment 5, framing, judicial news

et4P2_1 (Institutions alluded in parliamentary news (EXPERIMENT 1)):
et5P2_1 (Institutions alluded in politicians news (EXPERIMENT 1)):
et6P2_1 (Institutions alluded in judicial news (EXPERIMENT 1)):
Minimum: 1. Maximum: 10
1 = Spanish Parliament
2 = Spanish Government
3 = Regional Parliament
4 = Regional Government
5 = Spanish politicians
6 = Spanish political parties
7 = Spanish police
8 = Spanish judicial system
9 = European Parliament
10 = European Commission
. = .
.a = [DK]

em0_2 (Group in EXPERIMENT 2 (traditional media exposure)):
Minimum: 1. Maximum: 3
1 = Treatment 1, chosen media exposure
2 = Treatment 2, forced media exposure
3 = Control group, no media exposure
.z = [NA: not in wave]

emP1_2 (Journal chosen or assigned (EXPERIMENT 2, media exposure)):
Minimum: 1. Maximum: 5

1 = ABC
2 = El Mundo
3 = El País
4 = Infolibre
5 = Marca
.y = [NA: control group]
.z = [NA: not in wave]

emP2_2 (News' topic (EXPERIMENT 2, media exposure)):
Minimum: 1. Maximum: 99
1 = Immigration
2 = Economic crisis
3 = Unemployment
4 = Job insecurity
5 = Violence against women
6 = European integration
99 = None of the above
.a = [DK]
.z = [NA: not in wave]

emP3_2 (News' negative tone/sentiment (EXPERIMENT 2, media exposure)):
Minimum: 1. Maximum: 5
1 = Very positive
2 = Somewhat positive
3 = Neither positive nor negative
4 = Somewhat negative
5 = Very negative
.a = [DK]
.z = [NA: not in wave]

emP4_2 (The news helped position on the subject (EXPERIMENT 2, media exposure)):
Minimum: 1. Maximum: 4
1 = A lot
2 = Quite a lot
3 = A little bit
4 = Not at all
.a = [DK]
.z = [NA: not in wave]

esm0_3 (Group in EXPERIMENT 3):
Minimum: 1. Maximum: 2
1 = Control group, does not participate (group 3/1)
2 = Treatment, participates (group 3/0)
.z = [NA: not in wave]

esm0a_3 (Willingness to participate (EXPERIMENT 3)):

Minimum: 1. Maximum: 2
  1   = Yes
  2   = No
  .c   = [NA]
  .z   = [NA: not in wave]

esmP1_3 (Finally did EXPERIMENT 3):
Minimum: 1. Maximum: 2
  1   = Yes
  2   = No
  .y   = [NA: control group]
  .z   = [NA: not in wave]

esmP2a_3 (Followed the account of Pablo Casado (EXPERIMENT 3)):
esmP2b_3 (Followed the account of Pedro Sánchez (EXPERIMENT 3)):
esmP2c_3 (Followed the account of Pablo Iglesias (EXPERIMENT 3)):
esmP2d_3 (Followed the account of Albert Rivera (EXPERIMENT 3)):
esmP2e_3 (Followed the account of Santiago Abascal (EXPERIMENT 3)):
esmP2f_3 (Followed the account of Carles Puigdemont (EXPERIMENT 3)):
Minimum: 0. Maximum: 1
  0   = No
  1   = Yes
  .y   = [NA: control group]
  .z   = [NA: not in wave]

esmP3_3 (Followed the accounts for EXPERIMENT 3 or before):
Minimum: 1. Maximum: 3
  1   = Already followed before the experiment
  2   = Followed only now
  3   = Followed some and now follow some more
  .c   = [NA]
  .y   = [NA: control group]
  .z   = [NA: not in wave]

esmP3a_3 (Started following the account of Pablo Casado (EXPERIMENT 3)):
esmP3b_3 (Started following the account of Pedro Sánchez (EXPERIMENT 3)):
esmP3c_3 (Started following the account of Pablo Iglesias (EXPERIMENT 3)):
esmP3d_3 (Started following the account of Albert Rivera (EXPERIMENT 3)):
esmP3e_3 (Started following the account of Santiago Abascal (EXPERIMENT 3)):
esmP3f_3 (Started following the account of Carles Puigdemont (EXPERIMENT 3)):
Minimum: 0. Maximum: 1
  0   = No
  1   = Yes
  .y   = [NA: control group]
  .z   = [NA: not in wave]

esmP4a_3 (How frequently did Pablo Casado tweet (EXPERIMENT 3)):
esmP4b_3 (How frequently did Pedro Sánchez tweet (EXPERIMENT 3)):
esmP4c_3 (How frequently did Pablo Iglesias tweet (EXPERIMENT 3)):
esmP4d_3 (How frequently did Albert Rivera tweet (EXPERIMENT 3)):
esmP4e_3 (How frequently did Santiago Abascal tweet (EXPERIMENT 3)):
esmP4f_3 (How frequently did Carles Puigdemont tweet (EXPERIMENT 3)):
Minimum: 1. Maximum: 4
1 = Once per day
2 = 1 to 4 times a day
3 = 5 to 10 times a day
4 = More than 10 times a day
.a = [DK]
.c = [NA]
.y = [NA: control group]
.z = [NA: not in wave]

esmP5a1_3 (Pablo Casado mainly used text (EXPERIMENT 3)):
esmP5a2_3 (Pablo Casado mainly used images (EXPERIMENT 3)):
esmP5a3_3 (Pablo Casado mainly used videos (EXPERIMENT 3)):
esmP5a4_3 (Pablo Casado mainly used links to news (EXPERIMENT 3)):
esmP5a8_3 (Pablo Casado mainly used [dk] (EXPERIMENT 3)):
esmP5b1_3 (Pedro Sánchez mainly used text (EXPERIMENT 3)):
esmP5b2_3 (Pedro Sánchez mainly used images (EXPERIMENT 3)):
esmP5b3_3 (Pedro Sánchez mainly used videos (EXPERIMENT 3)):
esmP5b4_3 (Pedro Sánchez mainly used links to news (EXPERIMENT 3)):
esmP5b8_3 (Pedro Sánchez mainly used [dk] (EXPERIMENT 3)):
esmP5c1_3 (Pablo Iglesias mainly used text (EXPERIMENT 3)):
esmP5c2_3 (Pablo Iglesias mainly used images (EXPERIMENT 3)):
esmP5c3_3 (Pablo Iglesias mainly used videos (EXPERIMENT 3)):
esmP5c4_3 (Pablo Iglesias mainly used links to news (EXPERIMENT 3)):
esmP5c8_3 (Pablo Iglesias mainly used [dk] (EXPERIMENT 3)):
esmP5d1_3 (Albert Rivera mainly used text (EXPERIMENT 3)):
esmP5d2_3 (Albert Rivera mainly used images (EXPERIMENT 3)):
esmP5d3_3 (Albert Rivera mainly used videos (EXPERIMENT 3)):
esmP5d4_3 (Albert Rivera mainly used links to news (EXPERIMENT 3)):
esmP5d8_3 (Albert Rivera mainly used [dk] (EXPERIMENT 3)):
esmP5e1_3 (Santiago Abascal mainly used text (EXPERIMENT 3)):
esmP5e2_3 (Santiago Abascal mainly used images (EXPERIMENT 3)):
esmP5e3_3 (Santiago Abascal mainly used videos (EXPERIMENT 3)):
esmP5e4_3 (Santiago Abascal mainly used links to news (EXPERIMENT 3)):
esmP5e8_3 (Santiago Abascal mainly used [dk] (EXPERIMENT 3)):
esmP5f1_3 (Carles Puigdemont mainly used text (EXPERIMENT 3)):
esmP5f2_3 (Carles Puigdemont mainly used images (EXPERIMENT 3)):
esmP5f3_3 (Carles Puigdemont mainly used videos (EXPERIMENT 3)):
esmP5f4_3 (Carles Puigdemont mainly used links to news (EXPERIMENT 3)):
esmP5f8_3 (Carles Puigdemont mainly used [dk] (EXPERIMENT 3)):

Minimum: 0. Maximum: 1
0 = No
1 = Yes
.c = [NA]
.y = [NA: control group]
.z = [NA: not in wave]

esmP6a_3 (Hashtag associated to Pablo Casado (EXPERIMENT 3)):
esmP6b_3 (Hashtag associated to Pedro Sánchez (EXPERIMENT 3)):
esmP6c_3 (Hashtag associated to Pablo Iglesias (EXPERIMENT 3)):
esmP6d_3 (Hashtag associated to Albert Rivera (EXPERIMENT 3)):
esmP6e_3 (Hashtag associated to Santiago Abascal (EXPERIMENT 3)):
Minimum: 2. Maximum: 6
2 = #EspañaLoPrimero
3 = #SoyLiberal
4 = #SíSePuede
5 = #LaEspañaQueQuieres
6 = #ValorSeguro
.a = [DK]
.c = [NA]
.y = [NA: control group]
.z = [NA: not in wave]

esmP6f_3 (Hashtag associated to Carles Puigdemont (EXPERIMENT 3)):
Minimum: 1. Maximum: 1
1 = #FreeTothom
.a = [DK]
.c = [NA]
.y = [NA: control group]
.z = [NA: not in wave]

esm0_4 (Group in EXPERIMENT 4 (exposure to social media, EU elections)):
Minimum: 11. Maximum: 13
11 = Control group, group 4/2, does not participate
12 = Treatment 1, group 4/0, option A, leading candidates to EP
13 = Treatment 2, group 4/1, option B, EU institutions/Euronews
.z = [NA: not in wave]

esm0a_4 (Willingness to participate (EXPERIMENT 4)):
Minimum: 1. Maximum: 2
1 = Yes
2 = No
.c = [NA]
.z = [NA: not in wave]

esmP1_4 (Finally did EXPERIMENT 4):

Minimum: 1. Maximum: 2

1 = Yes
2 = No
.y = [NA: control group]
.z = [NA: not in wave]

esmP7_4 (Account followed in EXPERIMENT 4):

Minimum: 1. Maximum: 13

1 = Josep Borrell Fontelles @JosepBorrellF
2 = Maria Eugenia Rodríguez Palop @MEugeniaRPalop
3 = Luis Garicano Gabilondo @lugaricano
4 = Jorge Buxadé Villalba @Jorgebuxade
5 = Dolors Montserrat Montserrat @DolorsMM
6 = Jordi Sebastià Talavera (COMPROMÍS) @Sebastia_Jordi
7 = Oriol Junqueras @junqueras
8 = Carles Puigdemont @KRLS
9 = Izaskun Bilbao (EAJ-PNV) @IzaskunBilbaoB
10 = Parlamento Europeo @PE_Espana
11 = Parlamento Europeo @Europarl_ES
12 = Comisión Europea @UEmadrid
13 = Euronews español
.c = [NA]
.y = [NA: control group]
.z = [NA: not in wave]

esmP8_4 (Followed the account for EXPERIMENT 4 or before):

Minimum: 1. Maximum: 2

1 = I already followed it before
2 = I have followed it only now
.c = [NA]
.y = [NA: control group]
.z = [NA: not in wave]

esmP9_4 (Topics discussed in the account (EXPERIMENT 4)):

Minimum: 1. Maximum: 7

1 = The EU
2 = Catalonia and the independence process
3 = The unity of Spain
4 = The economic situation in Spain
5 = The social situation in Spain
6 = The territorial model in Spain
7 = Other current topics
.c = [NA]
.y = [NA: control group]
.z = [NA: not in wave]

esmP10_4 (Agreement with the opinions in the account (EXPERIMENT 4)):
Minimum: 1. Maximum: 5
1 = Agree strongly
2 = Somewhat agree
3 = Neither agree nor disagree
4 = Somewhat disagree
5 = Disagree strongly
.c = [NA]
.y = [NA: control group]
.z = [NA: not in wave]

esmP11_4 (Tone of the discussions in the account (EXPERIMENT 4)):
Minimum: 1. Maximum: 6
1 = Interesting
2 = Very intolerant
3 = Boring
4 = Disrespectful
5 = Informative
6 = None of the former
.a = [DK]
.c = [NA]
.y = [NA: control group]
.z = [NA: not in wave]

esmP12_4 (Trustworthiness of the accounts' information (EXPERIMENT 4)):
Minimum: 1. Maximum: 4
1 = Very trustworthy
2 = Somewhat trustworthy
3 = Little trustworthy
4 = No trustworthy at all
.c = [NA]
.y = [NA: control group]
.z = [NA: not in wave]
