# Supplementary material for: Political and affective polarisation in a democracy in crisis: The E-Dem panel survey dataset (Spain, 2018–2019)
Source: Data Brief. 2020 Jul 23;32:106059. doi: 10.1016/j.dib.2020.106059 (PMC7451797; doi:10.1016/j.dib.2020.106059)
Supplement: Supplementary file 2 [file mmc2.pdf]

Online political participation and deliberation in a democracy in crisis

Wave I Questionnaire

September 2018

Project: *Online Political Participation and Deliberation in a Democracy in Crisis: A New Methodological Approach (E-Dem) (2017-2020)*

PI: Mariano Torcal. Ministry of Economy and Competitiveness, State Programme for the Promotion of Scientific and Technical Research of Excellence, 2017. Ref: CSO2016-79772-P.

upf. Universitat Pompeu Fabra Barcelona

RECSM Research and Expertise Centre for Survey Methodology

[Instructions for Programmers]

1. Do not use the "I prefer not to answer".

2. Limit the use of "I don't know" to knowledge questions.

3. Do not force the panellist to answer all the questions.

4. Allow them not to answer the questions by moving on to the next one, although a warning message must be displayed to confirm that choice.

5. Respondents will receive a similar alert for selecting "don't know" for the knowledge questions.

6. In the data file, the name of variables must appear exactly as in the questionnaire.

7. It is also important to start the questionnaire with a short introduction:

A group of national and international researchers is interested in studying the opinion of people like you on issues related to our political system and our society. To this end, you are invited to participate in this survey which lasts approximately 20-25 minutes. About 2,500 people from all over Spain will participate in the study. Your answers are very important and will help us to learn the opinions of the Spanish people on the current political situation and the functioning of democracy in the country, and will remain anonymous for any data processing for scientific purposes.

8. Keep in mind the importance of your honest and timely answers.

9. Remember, throughout the online questionnaire, the respondent should be referred to in the informal "tú" (in Spanish) and not in formal "usted".

---

p1_1 To begin with, how much are you interested in politics? A lot, a fair amount, a little or not at all?

1 A lot
2 A fair amount
3 A little
4 Not at all

p2_1 To what extent are you satisfied with the general economic situation in Spain? Please indicate your answer on a scale from 0 to 10 where 0 is "Completely dissatisfied" and 10 is "Completely satisfied".

Completely dissatisfied										Completely satisfied
0	1	2	3	4	5	6	7	8	9	10

p3_1 In your opinion, what is the main problem that currently exists in Spain? Please choose one of the following options:

[PROGRAMMER: RANDOMLY ROTATE THE ORDER OF THE TOPICS FOR EACH RESPONDENT]

1 Unemployment
2 Drugs
3 The healthcare system
4 Housing
5 Education

6 Domestic ETA terrorism
7 International terrorism (Islamic State/ISIS)
8 Corruption
9 Immigration
10 The Euro
11 Violence against women
12 Political instability
13 The refugee crisis
14 Environmental problems
15 Pensions
16 Citizen insecurity
17 Taxes
18 Parties and politicians in general
19 Others_______
888 I don't know

In your opinion, how would you rate the situation in Spain with respect to the following issues? Please indicate your answer on a scale from 0 to 10 where 0 is "Very bad" and 10 is "Very good"

[PROGRAMMER: RANDOMLY ROTATE THE ORDER OF THE TOPICS FOR EACH RESPONDENT, SEPARATED INTO SCREENS WITH TWO ITEMS EACH FOR A TOTAL OF 3 SCREENS]

p4a_1 Unemployment

Very bad										Very good
0	1	2	3	4	5	6	7	8	9	10

p4b_1 Education

Very bad										Very good
0	1	2	3	4	5	6	7	8	9	10

p4c_1 Health

Very bad										Very good
0	1	2	3	4	5	6	7	8	9	10

p4d_1 Immigration

Very bad										Very good
0	1	2	3	4	5	6	7	8	9	10

p4e_1 The pension system

Very bad										Very good
0	1	2	3	4	5	6	7	8	9	10

p4f_1 Corruption

Very bad										Very good
0	1	2	3	4	5	6	7	8	9	10

p5_1 To what extent are you satisfied with the functioning of democracy in Spain? Please indicate your answer on a scale from 0 to 10 where 0 is "Completely dissatisfied" and 10 is "Completely satisfied"

Completely dissatisfied									Completely satisfied
0	1	2	3	4	5	6	7	8	9	10

p6_1 When talking about politics, people talk about "left" and "right". Could you please tell us where you would position yourself on a scale of 0 to 10 where 0 means "left" and 10 means "right"?

Left										Right
0	1	2	3	4	5	6	7	8	9	10

And where would you place each of the following political parties on this same scale?

[PROGRAMMER: RAMDONLY ROTATE THE ORDER OF THE PARTIES FOR EACH RESPONDENT]

		Left										Right
**p7a_1**	PP (People's Party)	0	1	2	3	4	5	6	7	8	9	10
**p7b_1**	PSOE (Spanish Socialist Workers' Party)	0	1	2	3	4	5	6	7	8	9	10
**p7c_1**	Podemos (En comú podem, Compromís, Equo, Iniciativa Catalunya Verds, En marea/	0	1	2	3	4	5	6	7	8	9	10
**p7d_1**	IU (United Left)	0	1	2	3	4	5	6	7	8	9	10
**p7e_1**	Ciuadanos (C's - Ciutadans)	0	1	2	3	4	5	6	7	8	9	10
**p7f_1**	ERC (Esquerra Republicana de Catalunya)	0	1	2	3	4	5	6	7	8	9	10
**p7g_1**	PDeCAT (Partit Demòcrata Europeu Català)	0	1	2	3	4	5	6	7	8	9	10
**p7h_1**	EAJ-PNV (Euzko Alderdi Jeltzalea, Basque Nationalist Party)	0	1	2	3	4	5	6	7	8	9	10
**p7i_1**	EH-Bildu (Euskal Herria- Bildu)	0	1	2	3	4	5	6	7	8	9	10
**p7j_1**	GBAI (Geroa Bai)	0	1	2	3	4	5	6	7	8	9	10
**p7k_1**	BNG (Galician Nationalist Block)	0	1	2	3	4	5	6	7	8	9	10

p8_1 Nowadays, the Autonomous Communities can legislate, together with the Government and the National Legislature, on some aspects of the citizens' daily life, such as health and education. However, not everyone considers that this should be the case.

On this subject, could you tell me where you would position yourself on the following scale from 0 to 10?

The Spanish Government should regain its powers										The Autonomous Communities should be able to legislate on major issues in citizens' daily lives
0	1	2	3	4	5	6	7	8	9	10

And on this same issue, where do you think the following political parties are positioned on this same scale?

[PROGRAMMER: RANDOMLY ROTATE THE ORDER OF THE PARTIES FOR EACH RESPONDENT]

		The Spanish Government should regain its powers										The Autonomous Communities should be able to legislate on major issues in citizens' daily lives
**p9a_1**	PP (People's Party)	0	1	2	3	4	5	6	7	8	9	10
**p9b_1**	PSOE (Spanish	0	1	2	3	4	5	6	7	8	9	10

	Socialist Workers' Party)											
**p9c_1**	Podemos (En comú podem, Compromís Equo; Iniciativa Catalunya Verds, En marea/ Anova)	0	1	2	3	4	5	6	7	8	9	10
**p9d_1**	IU (United Left)	0	1	2	3	4	5	6	7	8	9	10
**p9e_1**	Ciudadanos (C's - Ciutadans)	0	1	2	3	4	5	6	7	8	9	10
**p9f_1**	ERC (Esquerra Republicana de Catalunya)	0	1	2	3	4	5	6	7	8	9	10
**p9g_1**	PDeCAT (Partit Demòcrata Europeu Català)	0	1	2	3	4	5	6	7	8	9	10
**p9h_1**	EAJ-PNV (Euzko Alderdi Jeltzalea - Basque Nationalist Party)	0	1	2	3	4	5	6	7	8	9	10
**p9i_1**	EH-Bildu (Euskal Herria- Bildu)	0	1	2	3	4	5	6	7	8	9	10
**p9j_1**	GBAI (Geroa Bai)	0	1	2	3	4	5	6	7	8	9	10
**p9k_1**	BNG (Galician Nationalist Block)	0	1	2	3	4	5	6	7	8	9	10

Now we would like to know your opinion on some national issues that are the subject of public debate. Please indicate your response on a scale from 0 to 10

[PROGRAMMER: RANDOMLY ROTATE THE ORDER OF ITEMS p10a_1 to p10h_1 FOR EACH RESPONDENT)

p10a_1 Would you say that, in general, immigrants have to adapt to the customs of Spain and their region or that they should be able to maintain their customs despite living in another country?

They have to adapt to the customs of Spain										They should be able to keep their customs
0	1	2	3	4	5	6	7	8	9	10

p10b_1 And, do you think that private initiative (private companies) or, on the other hand, state intervention is the best way to solve the problems of the Spanish economy?

Private initiative is the best way										State intervention is the best way
0	1	2	3	4	5	6	7	8	9	10

p10c_1 Would you say that same-sex marriages should be prohibited or allowed by law?

They should be forbidden by law										They should be allowed by law
0	1	2	3	4	5	6	7	8	9	10

p10d_1 And, do you think that the main public services should be carried out by private companies or by public institutions of the State?

They should be carried out by private companies										They should be carried out by public institutions
0	1	2	3	4	5	6	7	8	9	10

p10e_1 Would you say that women should have the right to abortion?

Women should not have the right to abortion										Women should have the right to abortion
0	1	2	3	4	5	6	7	8	9	10

p10f_1 Would you say that income and wealth are distributed fairly among regular people in Spain or that wealth should be redistributed more fairly?

Wealth is fairly distributed										Wealth should be redistributed more fairly
0	1	2	3	4	5	6	7	8	9	10

p10g_1 And, do you think a woman should be prepared to give up her job for the sake of her family or should she be able to work?

She should be prepared to quit her job for the sake of her family										She should be able to work
0	1	2	3	4	5	6	7	8	9	10

p10h_1 Would you say that immigration to Spain should be reduced or increased?

Immigration to Spain should be reduced										Immigration to Spain should be increased
0	1	2	3	4	5	6	7	8	9	10

We would also like to know your feelings about some groups of people in Spanish society, using this thermometer.

Ratings between 60 and 100 mean that you have rather favourable feelings toward that group of people, with 100 being very favourable; while ratings between 0 and 40 mean instead that you have no favourable feelings toward the group, with 0 being very unfavourable. If you do not have particularly favourable or unfavourable feelings toward a group you should choose a 50 grade rating.

[PROGRAMMER: ROTATE THE ORDER OF QUESTIONS RANDOMLY WITHIN EACH GROUP]

[SOCIAL GROUPS]

		Unfavourable feelings				No feelings				Favourable feelings
**p11a_1**	The Basques	0	15	30	40	50	60	70	85	100
**p11b_1**	The Catalans	0	15	30	40	50	60	70	85	100
**p11c_1**	The people of Madrid	0	15	30	40	50	60	70	85	100
**p11d_1**	The Andalusians	0	15	30	40	50	60	70	85	100
**p11e_1**	Refugees	0	15	30	40	50	60	70	85	100

[VOTING GROUP] And what about these groups of people?

		Unfavourable				No				Favourable

		feelings				feelings				feelings
**p11f_1**	PP voters	0	15	30	40	50	60	70	85	100
**p11g_1**	PSOE voters	0	15	30	40	50	60	70	85	100
**p11h_1**	Ciudadanos voters	0	15	30	40	50	60	70	85	100
**p11i_1**	Podemos voters	0	15	30	40	50	60	70	85	100

[LEADERSHIP GROUP]

And what are your feelings about these leaders?

		Unfavourable feelings				No feelings				Favourable feelings
**p11j_1**	Pablo Casado	0	15	30	40	50	60	70	85	100
**p11k_1**	Pedro Sanchez	0	15	30	40	50	60	70	85	100
**p11l_1**	Albert Rivera	0	15	30	40	50	60	70	85	100
**p11m_1**	Pablo Iglesias	0	15	30	40	50	60	70	85	100
**p11n_1**	Iñigo Urkullu	0	15	30	40	50	60	70	85	100
**p11o_1**	Carles Puigdemont	0	15	30	40	50	60	70	85	100
**p11p_1**	Oriol Junqueras	0	15	30	40	50	60	70	85	100

PROGRAMMER: HERE WE HAVE DESIGNED TWO EXPERIMENTS ON DIMENSIONS OF POLITICAL TRUST IN POLITICAL AND SOCIAL INSTITUTIONS.

IN THE FIRST EXPERIMENT:
GROUP 1: (1/6 OF THE SAMPLE) ASK QUESTIONS et1a_1 TO et1j_1,
GROUP 2: (1/6 OF THE SAMPLE) QUESTIONS et2a_1 TO et2j_1
GROUP 3: (1/6 OF THE SAMPLE) RANDOMLY ASK QUESTIONS et3a_1 TO et3j_1
IN THE SECOND EXPERIMENT:
GROUP 4: (1/6 OF THE SAMPLE) ASK QUESTIONS et4a_1 TO et4j_1 IN RANDOM ORDER
GROUP 5: (1/6 OF THE SAMPLE) ASK QUESTIONS et5a_1 TO et5j_1 IN RANDOM ORDER
GROUP 6: (1/6 OF THE SAMPLE) ASK QUESTIONS et6a_1 TO et6j_1 IN RANDOM ORDER

THERE ARE THEREFORE A TOTAL OF 6 GROUPS. THE ASSIGNMENT TO EACH GROUP OF THE INDIVIDUALS IN THE SAMPLE HAS TO BE RANDOM.

[EXPERIMENT 1]

[GROUP 1: RANDOM BATTERY - CONTROL]

Could you please tell us on a scale of 0 to 10, where 0 is "I do not trust at all" and 10 is "I completely trust", how much you trust each of the following political institutions?

[PROGRAMMER: ROTATE THE ORDER OF QUESTIONS AT RANDOM FOR EACH INTERVIEWEE]

		Don't trust at									Completely trust

		all										
**et1a_1**	The Spanish Parliament	0	1	2	3	4	5	6	7	8	9	10
**et1b_1**	The Spanish government	0	1	2	3	4	5	6	7	8	9	10
**et1c_1**	The Parliament of **[PROGRAMMER:** AUTONOMOUS COMMUNITY **OF RESIDENCE]**	0	1	2	3	4	5	6	7	8	9	10
**et1d_1**	The government of **[PROGRAMMER:** AUTONOMOUS COMMUNITY **OF RESIDENCE]**	0	1	2	3	4	5	6	7	8	9	10
**et1e_1**	Politicians in Spain	0	1	2	3	4	5	6	7	8	9	10
**et1f_1**	Political parties in Spain	0	1	2	3	4	5	6	7	8	9	10
**et1g_1**	The Spanish police	0	1	2	3	4	5	6	7	8	9	10
**et1h_1**	The Spanish judicial system	0	1	2	3	4	5	6	7	8	9	10
**et1i_1**	The European Parliament	0	1	2	3	4	5	6	7	8	9	10
**et1j_1**	The government of the European Union (The European Commission)	0	1	2	3	4	5	6	7	8	9	10

[GROUP 2: QUESTIONS BY SCREENS]

[PROGRAMMER: ROTATE QUESTIONS RANDOMLY AND PRESENT THEM ON SEPARATE SCREENS]

Could you please tell us on a scale of 0 to 10, where 0 is "I don't trust at all" and 10 is "I completely trust", how much you trust each of the following political institutions...

[PROGRAMMER: PUT ON NEW SCREEN]

et2a_1 ...the Spanish Parliament

**I don't trust it at all**										**I completely trust it**
0	1	2	3	4	5	6	7	8	9	10

et2b_1 ...the Spanish government

[PROGRAMMER: PUT ON NEW SCREEN]

**I don't trust it at all**										**I completely trust it**
0	1	2	3	4	5	6	7	8	9	10

et2c_1 ...the Parliament of **[PROGRAMMER:** AUTONOMOUS COMMUNITY**]**

[PROGRAMMER: PUT ON NEW SCREEN]

**I don't trust it at all**										**I completely trust it**
0	1	2	3	4	5	6	7	8	9	10

et2d_1 ...the government of **[PROGRAMMER:** AUTONOMOUS COMMUNITY**]**

[PROGRAMMER: PUT ON NEW SCREEN]

**I don't trust it at all**										**I completely trust it**
0	1	2	3	4	5	6	7	8	9	10

et2e_1 ...politicians in Spain
[PROGRAMMER: PUT ON NEW SCREEN]

I don't trust them at all										I completely trust them
0	1	2	3	4	5	6	7	8	9	10

et2f_1 ...political parties in Spain

[PROGRAMMER: PUT ON NEW SCREEN]

I don't trust them at all										I completely trust them
0	1	2	3	4	5	6	7	8	9	10

et2g_1 ...the Spanish police

[PROGRAMMER: PUT ON NEW SCREEN]

I don't trust it at all										I completely trust it
0	1	2	3	4	5	6	7	8	9	10

et2h_1 ...the Spanish judicial system

[PROGRAMMER: PUT ON NEW SCREEN]

I don't trust it at all										I completely trust it
0	1	2	3	4	5	6	7	8	9	10

et2i_1 ...the European Parliament

[PROGRAMMER: PUT ON NEW SCREEN]

I don't trust it at all										I completely trust it
0	1	2	3	4	5	6	7	8	9	10

et2j_1 ...the government of the European Union (The European Commission)

[PROGRAMMER: PUT ON NEW SCREEN]

I don't trust it at all										I completely trust it
0	1	2	3	4	5	6	7	8	9	10

[GROUP 3: BLOCK QUESTIONS]

[PROGRAMMER: RANDOMLY ROTATE BLOCKS AND QUESTIONS WITHIN BLOCKS]

[PROGRAMMER: PUT ON A NEW SCREEN AND RANDOMLY ROTATE THE QUESTIONS]

Could you please tell us on a scale of 0 to 10, where 0 is "I do not trust at all" and 10 is "I completely trust", how much you trust each of the following political institutions?

et3a_1 ...the Spanish Parliament

I don't trust it at all										I completely trust it
0	1	2	3	4	5	6	7	8	9	10

et3b_1 ...the Spanish government

I don't trust it at all										I completely trust it
0	1	2	3	4	5	6	7	8	9	10

[PROGRAMMER: PUT ON A NEW SCREEN AND RANDOMLY ROTATE THE QUESTIONS]

And could you tell me the degree to which you trust

et3c_1...the Parliament of **[PROGRAMMER:** AUTONOMOUS COMMUNITY OF RESIDENCE]

I don't trust it at all										I completely trust it
0	1	2	3	4	5	6	7	8	9	10

et3d_1 ...the government of **[PROGRAMMER:** AUTONOMOUS COMMUNITY OF RESIDENCE]

I don't trust it at all										I completely trust it
0	1	2	3	4	5	6	7	8	9	10

[PROGRAMMER: PUT ON A NEW SCREEN AND RANDOMLY ROTATE THE QUESTIONS]

And could you tell me the degree to which you trust

et3e_1 ...politicians in Spain

I don't trust them at all										I completely trust them
0	1	2	3	4	5	6	7	8	9	10

et3f_1 ...political parties in Spain?

I don't trust them at all										I completely trust them
0	1	2	3	4	5	6	7	8	9	10

[PROGRAMMER: PUT ON A NEW SCREEN AND RANDOMLY ROTATE THE QUESTIONS]

And could you tell me the degree to which you trust

et3g_1 ...the Spanish police?

I don't trust it at all										I completely trust it
0	1	2	3	4	5	6	7	8	9	10

et3h_1 ...the Spanish judicial system?

I don't trust it at all										I completely trust it
0	1	2	3	4	5	6	7	8	9	10

And could you tell me the degree to which you trust

et3i_1 ...the European Parliament

[PROGRAMMER: PUT ON A NEW SCREEN AND RANDOMLY ROTATE THE QUESTIONS]

I don't trust it at all										I completely trust it
0	1	2	3	4	5	6	7	8	9	10

et3j_1 ...the government of the European Union (The European Commission)

I don't trust it at all										I completely trust it
0	1	2	3	4	5	6	7	8	9	10

[EXPERIMENT 2]

[GROUP 4: GOOD PERFORMANCE - *ACCOUNTABILITY* AND POLITICAL RENEWAL IN CONGRESS]

Now you will read a news item on some issues written by an expert to help you answer some questions.

One of the most debated issues in Spain today is the lack of renewal of the political class of our political representatives in the Spanish Congress. However, according to public data, the Congress of Deputies experiences significant turnover every term with a percentage of new parliamentarians that usually reaches 50%. Notably, in the last legislature that began in January 2016 there has been a heightened turnover with 62% new deputies, demonstrating the ability of Spanish parliamentary institutions to adapt to the challenges of the economic crisis and the needs of citizens. There has also been a change in terms of age. Since the last legislature, the average age of members of Parliament has decreased and with respect to political leaders, Spain is the country in the European Union with the second most youthful political leaders on average. Overall, according to various studies, we have a national parliament made up of young and well-trained representatives.

[PROGRAMMER: RANDOMLY ROTATE THE ORDER OF QUESTIONS FOR EACH RESPONDENT]

Could you please tell us on a scale of 0 to 10, where 0 is "I don't trust at all" and 10 is "I completely trust", how much you trust each of the following political institutions...

		I don't trust at all									I completely trust
**et4a_1**	the Spanish Parliament	0	1	2	3	4	5	6	7	8	9	10

**et4b_1**	the Spanish government	0	1	2	3	4	5	6	7	8	9	10
**et4c_1**	the Parliament of **[PROGRAMMER: NAME OF** AUTONOMOUS COMMUNITY]]	0	1	2	3	4	5	6	7	8	9	10
**et4d_1**	the government of **[PROGRAMMER: NAME OF** AUTONOMOUS COMMUNITY]	0	1	2	3	4	5	6	7	8	9	10
**et4e_1**	politicians in Spain	0	1	2	3	4	5	6	7	8	9	10
**et4f_1**	political parties in Spain	0	1	2	3	4	5	6	7	8	9	10
**et4g_1**	the Spanish police	0	1	2	3	4	5	6	7	8	9	10
**et4h_1**	the Spanish judicial system	0	1	2	3	4	5	6	7	8	9	10
**et4i_1**	the European Parliament	0	1	2	3	4	5	6	7	8	9	10
**et4j_1**	the government of the European Union (The European Commission)	0	1	2	3	4	5	6	7	8	9	10

[PROGRAMMER: ROTATE THE ORDER OF THE NEXT 3 QUESTIONS RANDOMLY]

Going back to the content of the news article you read earlier...

et4P1_1 Can you evaluate the content of the news on a scale from 0 to 10 where 0 is not at all positive and 10 is very positive?

**Not at all positive**										**Very positive**
0	1	2	3	4	5	6	7	8	9	10

et4P2_1 Can you tell me to which of the following institutions the content of the news refers?

1 the Spanish Parliament
2 the Spanish government
3 the Parliament of [PROGRAMMER: NAME OF AUTONOMOUS COMMUNITY]
4 the government of [PROGRAMMER: NAME OF AUTONOMOUS COMMUNITY]
5 politicians in Spain
6 political parties in Spain
7 the Spanish police
8 the Spanish judicial system
9 the European Parliament
10 the government of the European Union (The European Commission)

et4P3_1 Can you tell me how important the issue is for the functioning of the Spanish democratic system on a scale of 0 to 10 where 0 is "not important at all" and 10 is "very important"?

**Not at all important**										**Very important**
0	1	2	3	4	5	6	7	8	9	10

[GROUP 5: GOOD PERFORMANCE - 'COMPETENCE' OF SPANISH POLITICIANS]

Now you will read a news item on some issues written by an expert to help you answer some questions.

The negative belief that Spanish politicians are not sufficiently educated is not always based on real data. According to existing information on the subject, a high proportion of representatives are college-educated, for example: currently 90% of representatives hold university degrees in the autonomous regions (with the highest percentage in the Canary Islands, Navarre and Galicia) and 93% in the Spanish Congress. The educational credentials of our politicians have not ceased to increase since 1977 and puts Spain above the average of the European Union Member States. With regard to educational levels, the politicians who represent us today have more university graduates than ever before. So much that, for example, the Spanish Congress shows higher levels of education than other chambers of deputies in relevant countries such as Denmark or Finland.

[PROGRAMMER: RANDOMLY ROTATE THE ORDER OF QUESTIONS FOR EACH RESPONDENT]

Could you please tell us on a scale of 0 to 10, where 0 is "I don't trust at all" and 10 is "I completely trust", how much you trust each of the following political institutions...

		I don't trust at all										I completely trust
**et5a_1**	the Spanish Parliament	0	1	2	3	4	5	6	7	8	9	10
**et5b_1**	the Spanish government	0	1	2	3	4	5	6	7	8	9	10
**et5c_1**	the Parliament of **[PROGRAMMER: NAME OF** AUTONOMOUS COMMUNITY]]	0	1	2	3	4	5	6	7	8	9	10
**et5d_1**	the government of **[PROGRAMMER: NAME OF** AUTONOMOUS COMMUNITY]]	0	1	2	3	4	5	6	7	8	9	10
**et5e_1**	politicians in Spain	0	1	2	3	4	5	6	7	8	9	10
**et5f_1**	political parties in Spain	0	1	2	3	4	5	6	7	8	9	10
**et5g_1**	the Spanish police	0	1	2	3	4	5	6	7	8	9	10
**et5h_1**	the Spanish judicial system	0	1	2	3	4	5	6	7	8	9	10
**et5i_1**	the European Parliament	0	1	2	3	4	5	6	7	8	9	10
**et5j_1**	the government of the European Union (The European Commission)	0	1	2	3	4	5	6	7	8	9	10

[PROGRAMMER: ROTATE THE ORDER OF THE NEXT 3 QUESTIONS RANDOMLY]

Going back to the content of the news article you read...

et5P1_1 Can you evaluate the content of the news on a scale of 0 to 10 where 0 is "not at all positive" and 10 is " very positive"?

Not at all positive										Very positive
0	1	2	3	4	5	6	7	8	9	10

et5P2_1 Can you tell me to which of the following state institutions the content of the news refers?

1 the Spanish Parliament
2 the Spanish government
3 the Parliament of [PROGRAMMER: NAME OF AUTONOMOUS COMMUNITY]
4 the government of [PROGRAMMER: NAME OF AUTONOMOUS COMMUNITY]
5 politicians in Spain
6 political parties in Spain
7 the Spanish police
8 the Spanish judicial system
9 the European Parliament
10 the government of the European Union (The European Commission)

et5P3_1 Can you tell me how important the issue is for the functioning of the Spanish democratic system on a scale of 0 to 10 where 0 is "not at all important" and 10 is "very important"?

Not at all important										Very important
0	1	2	3	4	5	6	7	8	9	10

[GROUP 6: GOOD PERFORMANCE - RULE OF LAW EFFECTIVENESS]

Now you will read a news item on some issues written by an expert to help you answer some questions.

Despite its slowness, the Spanish judicial system in 2018 improved its score on the set of indicators on the efficiency, quality and independence of the judicial systems of the European Union, which places Spain in the middle range of Member States. According to public data, Spain stands out for the accessibility of its justice system to all those citizens who are 40% above the poverty line set by Eurostat, a level only reached in the EU by Denmark. With regard to independence, Spain is within European standards and in terms of judicial efficiency, the report is very positive in regard to the rate of resolution of civil matters. In terms of expenditure by public administrations on the whole of the judicial system, Spain appears on par with Denmark and Finland.

[PROGRAMMER: ROTATE THE ORDER OF QUESTIONS AT RANDOM FOR EACH RESPONDENT]

Could you please tell us on a scale of 0 to 10, where 0 is "I don't trust at all" and 10 is "I completely trust", how much you trust each of the following political institutions...

		I don't trust at all										I completely trust
**et6a_1**	the Spanish Parliament	0	1	2	3	4	5	6	7	8	9	10
**et6b_1**	the Spanish government	0	1	2	3	4	5	6	7	8	9	10
**et6c_1**	the Parliament of **[PROGRAMMER: NAME OF AUTONOMOUS COMMUNITY]**	0	1	2	3	4	5	6	7	8	9	10
**et6d_1**	the government of **[PROGRAMMER: NAME OF AUTONOMOUS COMMUNITY]**	0	1	2	3	4	5	6	7	8	9	10
**et6e_1**	politicians in Spain	0	1	2	3	4	5	6	7	8	9	10
**et6f_1**	political parties in Spain	0	1	2	3	4	5	6	7	8	9	10
**et6g_1**	the Spanish police	0	1	2	3	4	5	6	7	8	9	10
**et6h_1**	the Spanish judicial system	0	1	2	3	4	5	6	7	8	9	10
**et6i_1**	the European Parliament	0	1	2	3	4	5	6	7	8	9	10
**et6j_1**	the government of the European Union (The European Commission)	0	1	2	3	4	5	6	7	8	9	10

[PROGRAMMER: ROTATE THE ORDER OF THE NEXT 3 QUESTIONS AT RANDOM]

Going back to the content of the news article you read...

et6P1_1 Can you evaluate the content of the news on a scale of 0 to 10 where 0 is "not at all positive" and 10 is " very positive"?

Not at all positive										Very positive
0	1	2	3	4	5	6	7	8	9	10

et6P2_1 Can you tell me to which of the following state institutions the content of the news refers?

1 the Spanish Parliament
2 the Spanish government
3 the Parliament of [PROGRAMMER: NAME OF AUTONOMOUS COMMUNITY]
4 the government of [PROGRAMMER: NAME OF AUTONOMOUS COMMUNITY]
5 politicians in Spain
6 political parties in Spain
7 the Spanish police
8 the Spanish judicial system
9 the European Parliament
10 the government of the European Union (The European Commission)

et6P3_1 Can you tell me how important the issue is for the functioning of the Spanish democratic system on a scale of 0 to 10 where 0 is "not at all important" and 10 is "very important"?

Not at all important									Very important
0	1	2	3	4	5	6	7	8	9	10

--- -----------------------------------

[PROGRAMMER: END OF EXPERIMENT]

p12a_1 Would you say that, in general, you can trust most people or that you can never be too careful in dealing with others? Please place yourself on the following scale from 0 to 10.

You can never be too careful									Most people can be trusted
0	1	2	3	4	5	6	7	8	9	10

p12b_1 And do you think that most people would try to take advantage of you if they could, or that they would be honest with you?

Most people would try to take advantage of me									Most people would be honest with me
0	1	2	3	4	5	6	7	8	9	10

p12c_1 Would you say that most of the time people try to help others or that they mainly look out for themselves?

Most of the time people look out for themselves									Most of the time people try to help others
0	1	2	3	4	5	6	7	8	9	10

Now we would like to turn to how much you trust various groups of people. For each, indicate the extent to which you trust the people in that group on the following scale from 0 to 10.

[PROGRAMMER: ROTATE THE ORDER OF QUESTIONS AT RANDOM FOR EACH RESPONDENT]

		I don't trust at all										I completely trust
**p13a_1**	Your family	0	1	2	3	4	5	6	7	8	9	10
**p13b_1**	Your neighbours	0	1	2	3	4	5	6	7	8	9	10
**p13c_1**	People you know personally	0	1	2	3	4	5	6	7	8	9	10
**p13d_1**	People you meet for the first time	0	1	2	3	4	5	6	7	8	9	10
**p13e_1**	People of another religion	0	1	2	3	4	5	6	7	8	9	10
**p13f_1**	People of other nationalities	0	1	2	3	4	5	6	7	8	9	10
**p13g_1**	The Catalans	0	1	2	3	4	5	6	7	8	9	10
**p13h_1**	The Basques	0	1	2	3	4	5	6	7	8	9	10
**p13i_1**	People from Madrid	0	1	2	3	4	5	6	7	8	9	10
**p13j_1**	People from Andalusia	0	1	2	3	4	5	6	7	8	9	10

How much do you trust various groups of citizens? For each, indicate how much you trust the people in that group on a scale from 0 to 10.

[PROGRAMMER: ROTATE THE ORDER OF QUESTIONS AT RANDOM FOR EACH RESPONDENT]

		I don't trust at all										I completely trust
**p14a_1**	PP voters	0	1	2	3	4	5	6	7	8	9	10
**p14b_1**	PSOE voters	0	1	2	3	4	5	6	7	8	9	10
**p14c_1**	Ciudadanos voters	0	1	2	3	4	5	6	7	8	9	10
**p14d_1**	Podemos and IU voters	0	1	2	3	4	5	6	7	8	9	10
**p14e_1**	ERC voters	0	1	2	3	4	5	6	7	8	9	10
**p14f_1**	Those who vote for the PDeCAT	0	1	2	3	4	5	6	7	8	9	10
**p14g_1**	Those who vote for the EAJ-PNV	0	1	2	3	4	5	6	7	8	9	10

We all feel more or less connected to the territory or political community (town, city, region, etc.) in which we live, but some of us feel more connected to some places than others. To what extent do you identify with the following localities?

p15a_1 The town or city where I live

Do not identify at all									Identify strongly
0	1	2	3	4	5	6	7	8	9	10

p15b_1 Region or autonomous community where I live

Do not identify at all										Identify strongly
0	1	2	3	4	5	6	7	8	9	10

p15c_1 Spain

Do not identify at all										Identify strongly
0	1	2	3	4	5	6	7	8	9	10

p15d_1 Europe

Do not identify at all										Identify strongly
0	1	2	3	4	5	6	7	8	9	10

p16a_1 To what extent would you say that the political system in Spain allows people like you to have a say in the decisions of political authorities?

It doesn't allow it at all										It allows it completely
0	1	2	3	4	5	6	7	8	9	10

p16b_1 To what extent do you consider yourself capable of taking an active role in a group that deals with political issues?

You don't consider yourself at all capable										You consider yourself fully capable
0	1	2	3	4	5	6	7	8	9	10

p16c_1 And to what extent would you say that the political system in Spain allows people like you to have an influence in politics?

It doesn't allow it at all									It allows it completely
0	1	2	3	4	5	6	7	8	9	10

p16d_1 And to what extent do you trust your own ability to participate in politics?

I don't trust it at all										I have complete confidence
0	1	2	3	4	5	6	7	8	9	10

p16e_1 Finally, to what extent would you say that politicians care what people like you think?

Not at all										Completely
0	1	2	3	4	5	6	7	8	9	10

Now, indicate through what means and how often you are kept informed about current issues. Keep in mind the importance of reading the questions carefully and choosing the answer that best fits your thoughts and opinions. The results and quality of this international research depend on your effort and attention to your responses.

Could you please say how often you keep yourself informed about current political issues, news or opinions through...

[PROGRAMMER: ROTATE THE ORDER OF QUESTIONS RANDOMLY FOR EACH RESPONDENT]

		**Never**	**Less than once a month**	**Once a month**	**Several times a month**	**Once a week**	**Several times a week**	**Every day**	**Several times a day**
**p17a_1**	Newspapers	0	1	2	3	4	5	7	8
**p17b_1**	Radio	0	1	2	3	4	5	7	8
**p17c_1**	Magazines	0	1	2	3	4	5	7	8
**p17d_1**	Television	0	1	2	3	4	5	7	8
**p17e_1**	Social networks	0	1	2	3	4	5	7	8

p18_1 And how often do you keep yourself informed about current political issues, news or opinions through the Internet?

0 Never
1 Less than once a month
2 Once a month
3 Several times a month
4 Once a week
5 Several times a week
6 Every day
7 Several times a day
999 No access at home or at work - Not applicable

Do you have an account on one of the following social networks?

[PROGRAMMER: GO TO p26a_1 IF RESPONDENT SAYS NO TO ALL]

		**Yes**	**No**

**p19a_1**	Twitter	1	0
**p19b_1**	Facebook	1	0
**p19c_1**	Google +	1	0
**p19d_1**	LinkedIn	1	0
**p19e_1**	Instagram	1	0
**p19f_1**	Flickr	1	0
**p19g_1**	YouTube	1	0
**p19h_1**	Others	1	0

p20_1 How often do you usually connect to [PROGRAMMER: SET SOCIAL NETWORK NAME]?

[PROGRAMMER: ASK FOR EACH ONE IN WHICH THE RESPONDENT INDICATED "YES" AND NAME THE VARIABLES FROM p20a_1 TO p20h_1]

0 Never
1 Less than once a month
2 Once a month
3 Several times a month
4 Once a week
5 Several times a week
6 Every day
7 Several times a day

p21_1 When you use the social networks (Facebook, Twitter, Youtube or others) that you mentioned above, how often do you come across current news or political news?

[PROGRAMMER: PUT 999 TO THOSE WHO ANSWERED 0/NO ON ALL THE p19a_1-p19h_1]

0 Never
1 Sometimes
2 Often
3 Very often
4 Always

Could you please say to what extent the Internet and social networks have helped you to carry out the following activities?

[PROGRAMMER: ROTATE THE ORDER OF THE QUESTIONS RANDOMLY FOR EACH RESOPNDENT. PUT 999 FOR THOSE WHO ANSWERED 0/NO ON ALL p19a_1-p19h_1]

		**None**	**Little**	**Something**	**A lot**
**p22a_1**	Participate even more in groups and organizations to which you already belong	0	1	2	3
**p22b_1**	Participate in groups and organizations in which you did not participate before	0	1	2	3
**p22c_1**	Talk to people or groups who share your hobbies or interests	0	1	2	3
**p22d_1**	Talk to people or groups who share your religious beliefs	0	1	2	3
**p22e_1**	Talk to people or groups who share your political views	0	1	2	3
**p22f_1**	Talk to people from cultures other than your own	0	1	2	3
**p22g_1**	Talk to people of different ages or generations	0	1	2	3
**p22h_1**	Talk to people from other countries	0	1	2	3

p23a_1 You said earlier that you have a Twitter account. Could you please say to what extent the people you follow on Twitter have political opinions that are very similar to each other or hold a wide variety of political views...

PROGRAMMER: ASK ONLY THOSE WHO ANSWERED 1 ON p19a_1, IS TO SAY ONLY IF YOU HAVE DECLARED THAT YOU HAVE A TWITTER ACCOUNT. PUT 999 TO THOSE WHO ANSWERED 0/NO ON ALL THE p19a_1-p19h_1]

Very similar political views										Hold a wide variety of political views
0	1	2	3	4	5	6	7	8	9	10

p23b_1 And where would you place the views of the tweets you usually read?

PROGRAMMER: ASK ONLY THOSE WHO ANSWERED 1 ON p19a_1, (THOSE WHO HAVE DECLARED THAT THEY HAVE A TWITTER ACCOUNT). PUT 999 TO THOSE WHO ANSWERED 0/NO ON ALL THE p19a_1-p19h_1]

Almost all represent left-wing views										Almost all represent right-wing views
0	1	2	3	4	5	6	7	8	9	10

p24a_1 You said earlier that you have a Facebook account. Could you please say to what extent the people you follow on Facebook have political opinions that are very similar to each other or hold a wide variety of political views...

PROGRAMMER: ASK ONLY THOSE WHO ANSWERED 1 ON p19b_1 (THOSE WHO HAVE DECLARED THAT THEY HAVE A FACEBOOK ACCOUNT). PUT 999 TO THOSE WHO ANSWERED 0/NO ON ALL p19a_1-p19h_1]

Very similar political views										Hold a wide variety of political views
0	1	2	3	4	5	6	7	8	9	10

p24b_1 And where would you place the views of the posts you usually read on Facebook?

PROGRAMMER: ASK ONLY THOSE WHO ANSWERED 1 ON p19b_1 (THOSE WHO HAVE DECLARED THAT THEY HAVE A FACEBOOK ACCOUNT). PUT 999 TO THOSE WHO ANSWERED 0/NO ON ALL p19a_1-p19h_1]

Almost all represent left-wing views										Almost all represent right-wing views
0	1	2	3	4	5	6	7	8	9	10

p25_1 Do you follow any political parties or candidates on your social network accounts?

0 No, I don't follow any party or candidate on my social networks 1 Yes, I follow one party
2 Yes, I follow only one candidate
3 Yes, I follow only one party and more than one candidate from that same party
4 Yes, I follow more than one party and more than one candidate from different parties

p26a_1 How often do you discuss politics or political issues with family, friends, colleagues or acquaintances?

0 Never [GO **TO p26b_1]**
1 Less than once a month
2 Once a month
3 Several times a month
4 Once a week
5 Several times a week
6 Every day

p27a_1 How often do you agree with the views of the people with whom you talk about politics?

PROGRAMMER: ASK ONLY THOSE WHO ANSWERED 1-6 ON p26a_1

3 Always
2 Many times
1 Sometimes
0 Never

p28a_1 Also, how often do you disagree with the views of the people with whom you talk about politics?

[PROGRAMMER: ASK ONLY THOSE WHO ANSWERED 1-6 ON p26a_1]

3 Always
2 Many times
1 Sometimes
0 Never

p29a_1 Do you think the people you talk to about politics...?

[PROGRAMMER: ASK ONLY THOSE WHO ANSWERED 1-6 ON p26a_1]

3 They support the same party as you
2 They divide their support among different parties
1 They support a different party than yours
0 They do not support any party

p26b_1 How often do you discuss politics or current political issues on social networks, Facebook, Twitter or any other blog?

PROGRAMMER: ASK ONLY THOSE WHO ANSWERED 1/YES ON ONE OF THE p19a_1-p19h_1; PUT 999 FOR ALL OTHERS]

0 Never **[GO TO p30_1]**
1 Less than once a month
2 Once a month
3 Several times a month
4 Once a week
5 Several times a week
6 Every day

p27b_1 How often do you agree with the views of the people with whom you talk about politics in these forums?

PROGRAMMER: ASK ONLY THOSE WHO ANSWERED 1/YES ON ONE OF THE p19a_1-p19h_1 AND 1-6 ON P26b_1; PUT 999 FOR ALL THE OTHERS AND TO THOSE WHO ANSWERED 0 ON p26b_1

3 Always
2 Many times
1 Sometimes
0 Never

p28b_1 Also, how often do you disagree with the views of the people with whom you talk about politics in these forums?

PROGRAMMER: ASK ONLY THOSE WHO ANSWERED 1/YES ON ONE OF THE p19a_1-p19h_1 AND 1-6 ON P26b_1; PUT 999 FOR ALL THE OTHERS AND FOR THOSE WHO ANSWERED 0 ON p26b_1

3 Always
2 Many times
1 Sometimes
0 Never

p29b_1 Do you think that the people you talk to about politics in these forums...

PROGRAMMER: ASK ONLY THOSE WHO ANSWERED 1/YES ON ONE OF THE p19a_1-p19h_1 AND 1-6 ON P26b_1; PUT 999 FOR ALL THE OTHERS AND FOR THOSE WHO ANSWERED 0 ON p26b_1

3 They support the same party as you
2 They divide their support among different parties
1 They support a different party than yours
0 They do not support any party

Now we'll talk about aspects of your political preferences. Again, remember the importance of reading the questions carefully and choosing the answer that best fits your thoughts and opinions. The results and quality of this international research depend on your efforts and attention to detail. We remind you that your answers will remain anonymous and will only be treated, along with those of other respondents, in a statistical manner.

p30_1 It often happens that for different reasons, many people do not vote in elections. Did you vote in the last general election of June 2016?

1 Yes [GO **TO p30a_1]**
0 No [GO **TO p31_1]**
999 Not eligible to vote [GO TO **p31_1]**

p30a_1 For which political party or coalition did you vote?

[PROGRAMMER: ASK IF 1 ON p30_1]

1 PP (Popular Party)
2 PSOE (Spanish Socialist Workers' Party)
3 Unidas Podemos (We Can and local and municipal lists-IU Equo)
4 Ciudadanos - Citizenship Party (C's - Ciutadans)
5 ERC (Esquerra Republicana de Catalunya)
6 CDC (Democratic Convergence of Catalonia))
7 EAJ - PNV (Euzko Alderdi Jeltzalea - Basque Nationalist Party)
8 EH - Bildu (Euskal Herria - Bildu)
9 BNG (Bloque Nacionalista Galego)
10 CC (Canary Islands Coalition)
11 Other

p31_1 If you had voted in the last general election, for which party would you have voted?

PROGRAMMER: ASK IF 2 OR 999 ON p31_1

1 PP (Popular Party)
2 PSOE (Spanish Socialist Workers' Party)
3 Unidas Podemos (We Can and local and municipal lists-IU-Equo)
4 Ciudadanos - Citizenship Party (C's - Ciutadans)
5 ERC (Esquerra Republicana de Catalunya)
6 CDC (Democratic Convergence of Catalonia))
7 EAJ - PNV (Euzko Alderdi Jeltzalea - Basque Nationalist Party)
8 EH - Bildu (Euskal Herria - Bildu)
9 BNG (Bloque Nacionalista Galego)
10 CC (Canary Islands Coalition)
11 Other___________

There are many ways to try to make things better in Spain, or at least prevent them from getting worse. In the last 12 months have you done any of the following?

[PROGRAMMER: RANDOMLY ROTATE THE ORDER OF QUESTIONS FOR EACH RESPONDENT]

		**Yes**	**No**
**p32a_1**	You signed a petition in a signature collection campaign	1	0
**p32b_1**	You boycotted or stopped buying certain products	1	0
**p32c_1**	You have worn or shown any campaign badges or stickers	1	0
**p32d_1**	You have participated in authorized demonstrations	1	0
**p32e_1**	You've participated in political rallies	1	0
**P32f_1**	You have contacted a politician or a state, regional or local authority or official	1	0
**p32g_1**	You have contacted or appeared in the media to express your opinions	1	0

And, in the last 12 months, have you performed any of the following activities on the Internet?

[PROGRAMMER: RANDOMLY ROTATE THE ORDER OF QUESTIONS FOR EACH RESPONDENT]

		**Yes**	**No**
**p33a_1**	You visited the website of a party/candidate/political group	1	0
**p33b_1**	You followed a party/candidate/political group on Facebook	1	0
**p33c_1**	You sent an email to a party/candidate/political group	1	0
**p33d_1**	You sent a tweet to a party/candidate/political group on Twitter	1	0
**p33e_1**	You published political news/messages on social networks such as Facebook or Twitter	1	0
**p33f_1**	You have debated politics in a political forum or blogged	1	0
**p33g_1**	You signed a petition in a web-based signature campaign	1	0
**p33h_1**	You used the Internet to encourage people to vote in elections	1	0
**p33i_1**	You have tried to convince people to vote for a candidate/party by using social media	1	0
**p33j_1**	You have tried to convince people to vote for a candidate/party using emails	1	0
**p33k_1**	You participated in a political event to which you were invited via the Internet	1	0
**p33l_1**	You have participated in viral campaigns based on ironic or political memes on Twitter or Facebook	1	0

And, again in the last 12 months, have you done any of the following activities on social networks?

[PROGRAMMER: RANDOMLY ROTATE THE ORDER OF THE QUESTIONS FOR EACH RESPONDENT. ASK ONLY THOSE WHO ANSWERED 1/YES ON ONE OF THE p19a_1-p19h_1; PUT 999 FOR ALL OTHERS]

		Yes	No
**p34a_1**	You've followed someone or added friends with different political views	1	0
**p34b_1**	You've marked "like" for a political commentary or a tweet posted by others	1	0
**p34c_1**	You've shared a political message or tweet posted by others	1	0
**p34d_1**	You have responded with a positive comment to a political tweet or Facebook status posted by others	1	0
**p34e_1**	You have responded with a negative comment to a political tweet or Facebook status posted by others	1	0
**p34f_1**	You've stopped following, blocked or banned someone from your own contacts for political reasons	1	0
**p34g_1**	You decided not to publish political content because you were afraid of offending other people	1	0
**p34h_1**	You decided not to publish political content because you feared public exposure	1	0
**p34i_1**	You've changed your mind after participating in political discussions on Twitter or Facebook	1	0
**p34j_1**	You have increased your participation in a political cause after participating in or reading a debate on social networks	1	0
**p34k_1**	You have decreased your participation in a political cause after participating in or reading a debate on social networks	1	0

p35_1 Do you consider yourself close to any political party?

1 Yes **[GO TO p35a_1]**
0 No **[GO TO p36a_1]**

p35a_1 Which one?

[PROGRAMMER: ASK IF 1 ON p35_1. PUT 999 FOR THE OTHERS]

1 PP (Popular Party)
2 PSOE (Spanish Socialist Workers' Party)
3 Podemos and other affiliated municipal lists (En Comù Podem, En Marea, Ahora Madrid)
4 IU (United Left)
5 Ciudadanos (C's - Ciutadans)
6 ERC (Esquerra Republicana de Catalunya)
7 PDeCAT (Partit Demòcrata Europeu Català)
8 EAJ - PNV (Euzko Alderdi Jeltzalea - Basque Nationalist Party)
9 EH - Bildu (Euskal Herria - Bildu)
10 BNG (Bloque Nacionalista Galego) 11
CC (Canary Islands Coalition)
12 Others

p35b_1 And how close do you feel to this party?

[PROGRAMER: ASK IF 1 ON p35_1. PUT 999 FOR OTHERS]

3 Very close
2 Somewhat close
1 Not very close
0 Not at all close

p35c_1 Is it important for you to be from the [PUT PARTY NAME CHOSEN ON p35a_1]?

[PROGRAMER: ASK IF 1 ON p35_1. PUT 999 FOR OTHERS]

3 Extremely important
2 Very important
1 Not very important 0
Not at all important

p35d_1 How does the word supporters of [PUT A NAME ON p35a_1] describe you?

[PROGRAMER: ASK IF 1 ON p35_1. PUT 999 FOR OTHERS]

3 Extremely well
2 Fairly well
1 Not very well
0 Not at all

p35e_1 When you talk about [INSERT PARTY NAME CHOSEN IN p35a_1], how often do you use the word "we"?

3 Always
2 Most of the time 1
Sometimes
0 Never

[PROGRAMER: ASK IF 1 ON p35_1. PUT 999 FOR OTHERS]

p35f_1 To what extent do you consider yourself "one" of the [PUT PARTY NAME FROM p35a_1]?

[PROGRAMER: ASK IF 1 ON p35_1. PUT 999 FOR OTHERS]

3 A lot
2 Some
1 Little
0 Not at all

Now you will read some statements about the European Union and Spain. These questions are not a personal "test", it's just a matter of finding out how much knowledge people have about certain topics that are considered somewhat complicated. For each one, could you please indicate if you think it is true or false? If you don't know, just select "I don't know" and move on to the next one.

PROGRAMMER: LIMIT THE WAIT TIME TO ANSWER THIS QUESTION TO 30 SECONDS. CREATE A VARIABLE TO INDICATE IF THE RESPONDENT TOOK MORE THAN 30 SECONDS TO ANSWER EACH OF THESE QUESTIONS.

SHOW EACH STATEMENT ON A DIFFERENT SCREEN AND ROTATE RANDOMLY FOR EACH RESPONDENT

IF IT TAKES LONGER, IT SHOULD AUTOMATICALLY JUMP TO THE NEXT SCREEN AND CODE AS “DON'T KNOW” OR DID NOT RESPOND”

		**True**	**Fake**	**NS**
**p36a_1**	The Minister of Defence in Spain is Margarita Robles	1	2	888

**p36b_1**	The Spanish Congress has 525 deputies	1	2	888
**p36c_1**	A person must be 25 years of age or older to stand as a candidate in the Spanish general election	1	2	888
**p36d_1**	Each country in the EU elects the same number of representatives for the European Parliament	1	2	888
**p36e_1**	Norway is a member of the EU	1	2	888
**p36f_1**	The European Union is currently made up of 28 Member States, which still includes Great Britain	1	2	888

s1_1 Gender [PROGRAMMER: LOADED FROM PANEL]

s2_1 Age [PROGRAMMER: NUMBER, LOADED FROM PANEL]

s3b_1 Would you say you live in...

1 A big city
2 Suburb of a large town or city
3 A medium sized town
4 A small town
5 Rural area or village

s4b_1 What is the highest level of education you have completed?

0 Never been to school (no studies)
1 Less than 5 years of school (primary school not completed)
2 Former Primary Education (Certificate of Primary Studies) 3
Up to 5º of GBS
4 Primary Education (LOGSE)
5 Elementary Grade in Music and Dance
6 Elementary School
7 GBS
8 ESO
9 Upper Secondary School, BUP
10PREU, COU Former High School)
11 High School (LOGSE) 12
F.P. of Initiation
13 Social Guarantee Programs, Initial Professional Qualification Programs (PCPI)
14 F.P. Official
15 F.P. of 1st Grade (FPI)
16 C.F. de Grado Medio (Medium Technical)
17 C.F. of Medium Degree in Plastic Arts and Design
18 Medium Grade in Music and Dance
19 F.P. Mastery
20 2nd Grade F.P. (FPII)
21 C.F. de Grado Superior (Superior Technical)
22 C.F. of Superior Grade in Art Schools
23 Expertise, former schools of Nursing, Teaching or Social Work
24 Diploma, Degree (Bologna), Engineering or Technical Architecture, 3-year degree, Higher Diploma in Design
25 Degree, Master (Bologna), Higher Engineering, Architecture, Higher Degree in Music, Dance or Dramatic Art
26 PhD
27 Other (specify) ____

s5_1 You are...

1 Married
2 In a partnered relationship
3 Legally separated
4 Divorced

5 Widowed
6 None of the above (I have never been married)

s6_1 How many children do you have?

[NUMBER: ALLOW MINIMUM 0 AND MAXIMUM 6]

s7_1 Including yourself, how many people, adults and children, usually live in your house?

[NUMBER: ALLOW MINIMUM 1 AND MAXIMUM 10]

s8_1 And which best describes your situation in the last seven days? Please choose only one of the following options.

1 Employed, but on temporary leave (includes temporary maternity/paternity leave, accident, illness or holidays).
2. Employed, self-employed, or in a family business
2 Studying, even if you have been on holiday (includes company paid training)
3 Unemployed and actively seeking work
4 Unemployed, wanting to find a job but not actively looking for one
5 With chronic illness or permanent disability
6 Retired
7 Homemaker, stay-at-home parent, or caregiver

s9_1 Which of the statements below best describes how you feel about your current household income?

1 With our current income we live comfortably
2 With our current income we get by
3 With our current income we have difficulties
4 With our current income we have many difficulties

s10_1 Have you been fired or let go from your primary employment at any time in the past year?

1 Yes
2 No

Currently, to what extent do you feel concerned about...

[PROGRAMMER: ROTATE ORDER RANDOMLY FOR EACH RESPONDENT]

		Not at all concerned	Not too concerned	Quite concerned	Very concerned
**s11a_1**	Paying your household bills	0	1	2	3
**s11b_1**	Having to reduce your standard of living	0	1	2	3
**s11c_1**	Having a job	0	1	2	3
**s11d_1**	Paying off loans from the bank or paying mortgage bills	0	1	2	3

s12a_1 If you add up income from all sources, which letter below best describes your total household income after taxes and other required deductions? As you can see from the options below, it's not necessary to know or say an exact amount because you can select broad categories that have varying amounts. Use the information you know best: weekly, monthly or annual income.

	Approximately weekly	Approximately monthly	Approximately annual
A	195 less	780 or less	9350 or less
B	Over 195 euros up to 250 euros	More than 780 euros up to 1000 euros	More than 9350 euros up to 12000 euros
C	Over 251 euros up to 310 euros	Over 1001 euros up to 1250 euros	More than 12001 euros up to 15000 euros
D	More than 311 euros up to 375 euros	Over 1251 euros up to 1500 euros	More than 15001 euros up to 18000 euros
E	Over 376 euros up to 450 euros	More than 1501 euros up to 1800 euros	Over 18001 euros up to 21600 euros
F	More than 451 euros up to 550 euros	Over 1801 euros up to 2200 euros	More than 21601 euros up to 26400 euros
G	Over 551 euros up to 625 euros	Over 2201 euros up to 2500 euros	More than 26401 euros up to 30000 euros
H	More than 626 euros up to 710 euros	More than 2501 euros up to 2850 euros	More than 30001 up to 34200
I	Over 711 euros up to 925 euros	More than 2851 euros up to 3700 euros	More than 34201 euros up to 44400 euros
J	More than 926 euros	More than 3701 euros	More than 44401 euros

PROGRAMMER: EVERYONE CORRESPONDS TO THE FOLLOWING CATEGORIES: A=1, B=2, C=3, D=4, E=5, F=6, G=7, H=8, I=9, and J=10.

s13_1 And to what extent are you satisfied with your household's financial situation?

Completely dissatisfied										Completely satisfied
0	1	2	3	4	5	6	7	8	9	10

s14_1 Do you consider yourself to be of any religion?

1 Yes
0 No

s14a_1 Which one?

[PROGRAMMER: ASK ONLY IF 1 IN s14_1]

1 Catholic
2 Protestant
3 Orthodox
4 Other Christian denominations
5 Jewish
6 Muslim
7 Eastern religions (Buddhist, Hindu, Sikh, Shinto, Taoist)
8 Other non-Christian religions

s14b_1 Apart from special occasions such as weddings, baptisms and funerals, how often do you usually attend religious services?

6 Every day
5 More than once a week

4 Once a week
3 At least once a month
2 Only on special religious holidays
1 Very infrequently
0 Never
