# Supplementary material for: Political and affective polarisation in a democracy in crisis: The E-Dem panel survey dataset (Spain, 2018–2019)
Source: Data Brief. 2020 Jul 23;32:106059. doi: 10.1016/j.dib.2020.106059 (PMC7451797; doi:10.1016/j.dib.2020.106059)
Supplement: Supplementary file 3 [file mmc3.pdf]

Online political participation and deliberation in a democracy in crisis

Wave II Questionnaire

December 2018

Project: *Online Political Participation and Deliberation in a Democracy in Crisis: A New Methodological Approach (E-Dem) (2017-2020)*

PI: Mariano Torcal. Ministry of Economy and Competitiveness, State Programme for the Promotion of Scientific and Technical Research of Excellence, 2017. Ref: CSO2016-79772-P.

upf. Universitat Pompeu Fabra Barcelona

RECSM Research and Expertise Centre for Survey Methodology

[General instructions]

1. Do not force the panellist to answer all the questions.
2. Allow them not to answer the questions by moving on to the next one, although a warning message has to be issued where the respondent must confirm their choice every 4 questions.
3. In some important questions the message applies.
4. Some knowledge questions include the category "don't know" or "don't answer".
5. In the data file, the name of variables must appear exactly as in the questionnaire.
6. It is also important to start the questionnaire with this short introduction:

This survey aims to provide the data necessary to analyse aggregate opinions on current issues such as immigration or the pension system, as well as on political trends. It is a study led by researchers from the Pompeu Fabra University, within a European research project on online political participation and deliberation. All the information you and other respondents provide will be shared with that University anonymously and used only for the research purposes mentioned above. If you wish to exercise your right to data protection, you can write to the Department of Political Science at Pompeu Fabra University, located at c/ Ramon Trias Fargas, 25-27, 08005 Barcelona (Edificio Jaume I - Campus Ciutadella) or to the following e-mail address: departament.cpis@upf.edu

Below, we ask you to confirm if you would like to participate in this interesting survey:

- **Yes, I want to participate**
- **No, I'd rather not participate**

Second screen:

As you may remember, a few weeks ago you participated in a survey of a study designed by a group of national and international researchers led by a professor at Pompeu Fabra University in Barcelona who is interested in studying the opinion of people like you on issues related to our political system and our society. To this end, they would like to count on your participation in another survey that will last approximately 20-25 minutes. In this way you will be part of the same 2500 people from all over Spain who also participated in the previous one.

Your answers are very important and will help to know the opinions of the Spanish people about the political current situation and the functioning of the democracy in the country. The problems and challenges of our political and social systems require a good study of citizens' opinions and therefore your attentive and dedicated participation is essential. In any case, as with the previous survey, your answers will remain in total anonymity and will only be subject to statistical analysis together with the other hundreds of participants.

7. Remember from time to time the importance of your honest and timely answers.

8. Remember, throughout the online questionnaire, the respondent should be referred to in the informal "tú" (in Spanish) and not in formal "usted".

---

p1_2 To begin with, how much are you interested in politics? A lot, a fair amount, a little or not at all?

1 A lot
2 A fair amount
3 A little
4 Not at all

p2_2 To what extent are you satisfied with the general economic situation in Spain? Please indicate your answer on a scale from 0 to 10 where 0 is "Completely dissatisfied" and 10 is "Completely satisfied".

[PROGRAMMER: VERTICAL ORIENTATION ON MOBILE DEVICES]

Completely dissatisfied										Completely satisfied
0	1	2	3	4	5	6	7	8	9	10

p3_2 What, in your opinion, is the main problem that currently exists in Spain? Please choose one of the following options

[PROGRAMMER: ROTATE THE ORDER OF THE TOPICS AT RANDOM FOR EACH RESPONDENT]

1 Unemployment
2 Drugs
3 The healthcare system
4 Housing
5 Education
6 Domestic ETA terrorism
7 International terrorism (Islamic State/ISIS)
8 Corruption
9 Immigration
10 The Euro
11 Violence against women
12 Political instability
13 The refugee crisis
14 Environmental problems
15 Pensions
16 Citizen insecurity
17 Taxes
18 Parties and politicians in general
19 Other________
888 I don't know

In your opinion, how would you rate the situation in Spain with respect to the following issues? Please indicate your answer on a scale from 0 to 10 where 0 is "Very bad" and 10 is "Very good

[PROGRAMMER: ROTATE THE ORDER OF THE TOPICS AT RANDOM FOR EACH RESPONDENT, SEPARATED INTO SCREENS WITH TWO ITEMS ON EACH FOR A TOTAL OF 3 SCREENS]

[PROGRAMMER: VERTICAL ORIENTATION ON MOBILE DEVICES]

p4a_2 Unemployment

Very bad										Very good
0	1	2	3	4	5	6	7	8	9	10

PROGRAMMER: DON'T KNOW...888 (GENERATED AUTOMATICALLY IF RESPONDENT SKIPS WITHOUT ANSWERING AND AFTER INSISTING)

p4b_2 Education

Very bad										Very good
0	1	2	3	4	5	6	7	8	9	10

PROGRAMMER: DON'T KNOW...888
(GENERATED AUTOMATICALLY IF RESPONDENT SKIPS WITHOUT ANSWERING AND AFTER INSISTING)

p4c_2 Health

Very bad										Very good
0	1	2	3	4	5	6	7	8	9	10

PROGRAMMER: DON'T KNOW...888
(GENERATED AUTOMATICALLY IF RESPONDENT SKIPS WITHOUT ANSWERING AND AFTER INSISTING)

p4d_2 Immigration

Very bad										Very good
0	1	2	3	4	5	6	7	8	9	10

PROGRAMMER: DON'T KNOW...888
(GENERATED AUTOMATICALLY IF RESPONDENT SKIPS WITHOUT ANSWERING AND AFTER INSISTING)

p4e_2 The pension system

Very bad										Very good
0	1	2	3	4	5	6	7	8	9	10

PROGRAMMER: DON'T KNOW...888
(GENERATED AUTOMATICALLY IF RESPONDENT SKIPS WITHOUT ANSWERING AND AFTER INSISTING)

p4f_2 Corruption

Very bad										Very good
0	1	2	3	4	5	6	7	8	9	10

PROGRAMMER: DON'T KNOW...888
(GENERATED AUTOMATICALLY IF RESPONDENT SKIPS WITHOUT ANSWERING AND AFTER INSISTING)

p4g_2 Violence against women

Very bad										Very good
0	1	2	3	4	5	6	7	8	9	10

PROGRAMMER: DON'T KNOW...888
(GENERATED AUTOMATICALLY IF RESPONDENT SKIPS WITHOUT ANSWERING AND AFTER INSISTING)

p37a_2 And, how has the economic situation in Spain changed in the last 12 months?

1 It's gotten much worse
2 It's gotten a little worse
3 It's the same
4 It’s gotten a little better
5 It's gotten much better

p37b_2 And, how has the economic situation of your household changed in the last 12 months?

1 It's gotten much worse
2 It's gotten a little worse

3 It's the same
4 It’s gotten a little better
5 It's gotten much better

p38a_2 Now, to what extent are you satisfied with the work of the current Spanish government so far? Please indicate your answer on a scale from 0 to 10 where 0 is "Completely dissatisfied" and 10 is "Completely satisfied".

[PROGRAMMER: VERTICAL ORIENTATION ON MOBILE DEVICES]

Completely dissatisfied										Completely satisfied
0	1	2	3	4	5	6	7	8	9	10

p38b_2 Now, to what extent are you satisfied with the work of the PP as the main opposition party so far? Indicate your answer on a scale from 0 to 10 where 0 is "Completely dissatisfied" and 10 is "Completely satisfied".

[PROGRAMMER: VERTICAL ORIENTATION ON MOBILE DEVICES]

Completely dissatisfied										Completely satisfied
0	1	2	3	4	5	6	7	8	9	10

p5_2 To what extent are you satisfied with the functioning of democracy in Spain? Please indicate your answer on a scale from 0 to 10 where 0 is "Completely dissatisfied" and 10 is "Completely satisfied”.

[PROGRAMMER: VERTICAL ORIENTATION ON MOBILE DEVICES]

Completely dissatisfied										Completely satisfied
0	1	2	3	4	5	6	7	8	9	10

For the following list of voluntary organizations, could you tell me which of the following applies to you for the last 12 months? (PROGRAMMER: MORE THAN ONE MAY BE NOTED IF APPLICABLE).

[PROGRAMMER: ROTATE THE ORDER OF THE ORGANIZATIONS RANDOMLY FOR EACH RESPONDENT, PORTRAIT ORIENTATION, AND SEPARATE INTO SCREENS WITH TWO OR THREE ITEMS PER EACH SCREEN]

		**I do not belong and have not begun any activity**	**I am a member**	**I have participated in this type of activity some**	**I've donated money**	**I've done volunteer work**
**p39a_2**	Church or religious group	0	1	2	3	4
**p39b_2**	Sports organization	0	1	2	3	4
**p39c_2**	Educational, music or art organization	0	1	2	3	4
**p39d_2**	Labour or workers’ union	0	1	2	3	4
**p39e_2**	Political party	0	1	2	3	4
**p39f_2**	Environmental organization	0	1	2	3	4

**p39g_2**	Professional or business association	0	1	2	3	4
**p39h_2**	Humanitarian or charitable organization	0	1	2	3	4
**p39i_2**	Consumer organization	0	1	2	3	4
**p39l_2**	Organizations that offer social assistance and services or those in need	0	1	2	3	4
**p39m_2**	Other organizations: which ones?___________	0	1	2	3	4

p6_2 When talking about politics, people talk about "left" and "right". Could you please tell us where you would position yourself on a scale of 0 to 10 where 0 means "left" and 10 means "right"?

[PROGRAMMER: VERTICAL ORIENTATION ON MOBILE DEVICES]

**Left**										**Right**
0	1	2	3	4	5	6	7	8	9	10

PROGRAMMER: IF RESPONDENT ADVANCES WITHOUT ANSWERING, PRESENT A MESSAGE THAT SAYS "YOU WILL ADVANCE WITHOUT ANSWERING THIS QUESTION AND YOUR ANSWER WILL BE RECORDED AS "DON'T KNOW / DON'T ANSWER", DO YOU AGREE? WITH RESPONSE OPTIONS "YES" AND "NO".

PROGRAMMER: DON'T KNOW...888 (GENERATED AUTOMATICALLY IF RESPONDENT SKIPS WITHOUT ANSWERING AND AFTER INSISTING)

And where would you place each of the following political parties on this same scale?

[PROGRAMMER: ROTATE THE ORDER OF THE ITEMS AT RANDOM FOR EACH RESPONDENT]

[PROGRAMMER: ON MOBILE DEVICES, ROTATE RANDOMLY FOR EACH RESPONDENT, VERTICAL ORIENTATION AND SEPARATE INTO SCREENS WITH TWO OR THREE ITEMS PER SCREEN].

		**Left**										**Right**	**I don't know**
**p7a_2**	PP (People's Party)	0	1	2	3	4	5	6	7	8	9	10	888
**p7b_2**	PSOE (Spanish Socialist Workers' Party)	0	1	2	3	4	5	6	7	8	9	10	888
**p7c_2**	Podemos (En comú podem, Compromís, Equo, Iniciativa Catalunya Verds, En marea/ Anova)	0	1	2	3	4	5	6	7	8	9	10	888
**p7d_2**	IU (United Left)	0	1	2	3	4	5	6	7	8	9	10	888
**p7e_2**	Ciudadanos (C's - Ciutadans)	0	1	2	3	4	5	6	7	8	9	10	888
**p7f_2**	ERC (Esquerra Republicana de Catalunya)	0	1	2	3	4	5	6	7	8	9	10	888
**p7g_2**	PDeCAT (Partit Demòcrata Europeu Català)	0	1	2	3	4	5	6	7	8	9	10	888
**p7h_2**	EAJ-PNV (Euzko Alderdi Jeltzalea, Basque Nationalist Party)	0	1	2	3	4	5	6	7	8	9	10	888
**p7i_2**	EH-Bildu (Euskal Herria- Bildu)	0	1	2	3	4	5	6	7	8	9	10	888
**p7j_2**	GBAI (Geroa Bai)	0	1	2	3	4	5	6	7	8	9	10	888

**p7k_2**	BNG (Galician Nationalist Block)	0	1	2	3	4	5	6	7	8	9	10	888
**p7l_2**	Vox	0	1	2	3	4	5	6	7	8	9	10	888

p8_2 Nowadays, the Autonomous Communities can legislate, together with the Government and the National Legislature, on some aspects of the citizens' daily life, such as health and education.

On this subject, could you tell me where you would position yourself on the next scale from 0 to 10?

[PROGRAMMER: VERTICAL ORIENTATION ON MOBILE DEVICES]

The Spanish Government should regain its powers									The Autonomous Communities should be able to legislate on major issues in citizens' daily lives
0	1	2	3	4	5	6	7	8	9	10

And on this same issue, where would you place each of the following political parties on this same scale?

[PROGRAMMER: ROTATE THE ORDER OF THE ITEMS AT RANDOM FOR EACH INTERVIEWEE]

[PROGRAMMER: ON MOBILE DEVICES, RANDOMLY ROTATE THE ORDER FOR EACH RESPONDENT, VERTICAL ORIENTATION AND SEPARATE INTO SCREENS WITH TWO OR THREE ITEMS PER SCREEN].

		The Spanish Government should regain its powers										The Autonomous Communities should be able to legislate on major issues in citizens' daily lives	I don't know
**p9a_2**	PP (People's Party)	0	1	2	3	4	5	6	7	8	9	10	888
**p9b_2**	PSOE (Spanish Socialist Workers' Party)	0	1	2	3	4	5	6	7	8	9	10	888
**p9c_2**	Unidas POdemos (In common we can Compromís Equo; Iniciativa Catalunya Verds, En marea/	0	1	2	3	4	5	6	7	8	9	10	888
**p9d_2**	IU (United Left)	0	1	2	3	4	5	6	7	8	9	10	888
**p9e_2**	Ciudadanos (C's	0	1	2	3	4	5	6	7	8	9	10	888
**p9f_2**	ERC (Esquerra Republicana	0	1	2	3	4	5	6	7	8	9	10	888

	de Catalunya)												
**p9g_2**	PDeCAT (Partit Demòcrata Europeu Català)	0	1	2	3	4	5	6	7	8	9	10	888
**p9h_2**	EAJ-PNV (Euzko Alderdi Jeltzalea - Basque Nationalist Party)	0	1	2	3	4	5	6	7	8	9	10	888
**p9i_2**	EH-Bildu (Euskal Herria-Bildu)	0	1	2	3	4	5	6	7	8	9	10	888
**p9j_2**	GBAI (Geroa Bai)	0	1	2	3	4	5	6	7	8	9	10	888
**p9k_2**	BNG (Galician Nationalist Block)	0	1	2	3	4	5	6	7	8	9	10	888
**p9l_2**	Vox	0	1	2	3	4	5	6	7	8	9	10	888

--- ----------------------

PROGRAMMER: IN THIS SECTION WE HAVE AN EXPERIMENT ON SELECTIVE EXPOSURE AND POLITICAL POLARIZATION

FOUR ARTICLES ABOUT IMMIGRATION WILL BE SELECTED FROM 4 NATIONAL NEWSPAPERS: ABC, EL MUNDO, EL PAIS, DIARIO.ES; AND A SPORTS JOURNAL (MARCA) (IN THIS LAST ONE WE WILL SELECT SOMETHING ABOUT FOREIGNERS PLAYING SOCCER IN SPAIN).

THERE MUST BE A CONTROL GROUP OF 20% OF THE SAMPLE.

THE EXPERIMENT WILL BE CONDUCTED IN TWO STEPS.

IN THE FIRST ONE, PANELLISTS WILL BE RANDOMLY ASSIGNED TO THREE GROUPS:

- **GROUP 1: (40% OF THE SAMPLE): "CHOICE CONDITION"**
- **GROUP 2: (40% OF THE SAMPLE): "FORCED CONDITION"**
- **GROUP 0: (20% OF THE SAMPLE): NO TREATMENT, "CONTROL"**

PROGRAMMER: THERE SHOULD BE A VARIABLE CALLED "CHOICE" THAT RECORDS WHETHER THE RESPONDENT HAS BEEN ASSIGNED TO CONTROL (0), A CHOICE (1), FORCED (2).

IN "CHOICE CONDITION" RESPONDENTS ARE GIVEN THE POSSIBILITY TO CHOOSE BETWEEN 5 NEWSPAPERS: ABC, "EL MUNDO", "EL PAIS", "INFOLIBRE" AND THE SPORTS JOURNAL BRAND "MARCA" (IN THIS LAST ONE WE WILL SELECT SOMETHING ABOUT FOREIGNERS PLAYING SOCCER IN SPAIN).

IN "FORCED CONDITION" RESPONDENTS ARE RANDOMLY ASSIGNED TO ONE OF THESE 5 NEWSPAPERS AND THEIR NEWS ABOUT IMMIGRATION. THE NEWSPAPER SOURCE IS PROVIDED TO THE RESPONDENT.

THE TREATMENT IS TO ALLOW THE RESPONDENT TO CHOOSE OR NOT AND THEN SEE WHETHER OR NOT THE NEWS THEY VIEW CORRESPONDS TO THEIR IDEOLOGICAL AND PARTISAN PREFERENCES.

IN THIS EXPERIMENT WE NEED THE RESPONDENTS IN THE "FORCED" TREATMENT TO KNOW THAT THEY ARE IN THE SELECTED NEWSPAPER ONCE THEY HAVE CLICKED ON THE LINK TO SEE THE NEWS (NOT BEFORE).

PARTISAN PREFERENCES ARE OBTAINED FROM A QUESTION IN WAVE 1, BUT WE BRING THESE TOGETHER WITH THE DATA LATER ON.

WE NEED A VARIABLE THE TIME SPENT READING THE NEWS.

WITH THE PASSIVE METER WE NEED TO KNOW IF THE RESPONDENT IS A REGULAR READER OF THAT PAPER. THIS INFORMATION IS ALREADY COLLECTED.

---

emP1_2 We are interested in hearing your opinion on some current issues. Since we want to know your opinion, and not so much what you know or don't know about this topic, we are going to ask you to read a press article about one of the topics we are interested in knowing your opinion about. To do this, we give you four options and you choose the newspaper where you prefer to read this information:

1. El País
2. ABC
3. Infolibre
4. El Mundo
5. Marca

PROGRAMMER: ROTATE THE ORDER AT RANDOM.

PROGRAMMER: FOR THE "NO CHOICE" CONDITION, AUTOMATICALLY RECORD THE ASSIGNED JOURNAL.

emP2_2 Could you tell me what the story you just read was mainly about?

PROGRAMMER: RANDOMLY ROTATE THE ORDER OF THE FOLLOWING BY GROUPS OF QUESTIONS

1. Immigration
2. The economic crisis
3. Unemployment
4. The precariousness of work
5. Violence against women
6. European integration
7. None of the above

emP3_2 Could you tell me if the position of this article is positive or negative on this issue?

1. Very positive
2. Somewhat positive
3. Neither positive nor negative
4. Somewhat negative
5. Very negative

emP4_2 Could you tell me if reading this article has helped you to take a position on this issue?

1. A lot
2. Quite a lot
3. Little
4. Nothing

PROGRAMMER: RANDOMLY ROTATE THE ORDER OF THE FOLLOWING BY GROUPS OF QUESTIONS

Now we would like to know your opinion on some national issues that are the subject of public debate. Please indicate your response on a scale from 0 to 10

[PROGRAMMER: RANDOMLY ROTATE ITEMS p10a_2 to p10h_2 FOR EACH RESPONDENT, VERTICAL ORIENTATION ON MOBILE DEVICES]

p10a_2 In general, would you say that immigrants have to adapt to the customs of Spain and the region in which they live or that they can maintain their customs in spite of living in another country?

They have to adapt to the customs of Spain										They can keep their customs
0	1	2	3	4	5	6	7	8	9	10

p10b_2 And, do you think that private initiative (private companies) or, on the other hand, state intervention is the best way to solve the problems of the Spanish economy?

Private initiative is the best way										State intervention is the best way
0	1	2	3	4	5	6	7	8	9	10

p10c_2 Would you say that same-sex marriages should be prohibited or allowed by law?

They should be forbidden by law										They should be allowed by law
0	1	2	3	4	5	6	7	8	9	10

p10d_2 And, do you think that the main public services should be carried out by private companies or by public institutions of the State?

They should be carried out by private companies										They should be carried out by public institutions
0	1	2	3	4	5	6	7	8	9	10

p10e_2 Would you say that women should have the right to abortion?

Women should not have the right to abortion										Women should have the right to abortion
0	1	2	3	4	5	6	7	8	9	10

p10f_2 Would you say that income and wealth are distributed fairly among the regular people in Spain or that wealth should be redistributed more fairly?

Wealth is fairly distributed										Wealth should be redistributed more fairly
0	1	2	3	4	5	6	7	8	9	10

p10g_2 And, do you think a woman should be prepared to give up her job for the sake of her family or should she be able to work?

She must be prepared to quit her job for the sake of her family										She must be able to work
0	1	2	3	4	5	6	7	8	9	10

p10h_2 Would you say that immigration to Spain should be reduced or increased?

Immigration to Spain should be										Immigration to Spain should be

reduced									increased
0	1	2	3	4	5	6	7	8	9	10

FOR QUESTIONS p10A_2 TO p10H_2 PROGRAMMER: DON'T KNOW...888 (GENERATED AUTOMATICALLY IF RESPONDENT SKIPS WITHOUT ANSWERING AND AFTER INSISTING)

FOR QUESTIONS p10A_2 TO p10H_2 PROGRAMMER: IF RESPONDENT ADVANCES WITHOUT ANSWERING, PLEASE PRESENT A MESSAGE THAT SAYS "IF YOU ADVANCE WITHOUT ANSWERING THIS QUESTION, YOUR ANSWER WILL BE RECORDED AS "DON'T KNOW / DON'T ANSWER", DO YOU AGREE? AND PRESENT

We would also like to know your feelings about some groups of people in Spanish society, using this thermometer.

Ratings between 60 and 100 mean that you have rather favourable feelings toward that group of people, with 100 being very favourable; while ratings between 0 and 40 mean instead that you have no favourable feelings toward the group, with 0 being very unfavourable. If you do not have particularly favourable or unfavourable feelings toward a group you should choose a 50 grade rating.

[PROGRAMMER: ROTATE THE ORDER OF QUESTIONS RANDOMLY WITHIN EACH GROUP]

[SOCIAL GROUPS]

[PROGRAMMER: ON MOBILE DEVICES, RANDOMLY ROTATE ORDER OF ITEMS FOR EACH RESPONDENT, VERTICAL ORIENTATION AND SEPARATED INTO SCREENS WITH TWO OR THREE ITEMS PER SCREEN].

		Unfavourable feelings				No feelings				Favourable feelings
**p11a_2**	The Basques	0	15	30	40	50	60	70	85	100
**p11b_2**	The Catalans	0	15	30	40	50	60	70	85	100
**p11c_2**	The people of Madrid	0	15	30	40	50	60	70	85	100
**p11d_2**	The Andalusians	0	15	30	40	50	60	70	85	100
**p11e_2**	Refugees	0	15	30	40	50	60	70	85	100
**p11s_2**	Immigrants	0	15	30	40	50	60	70	85	100

[VOTING GROUPS]

And what about these groups of people?

[PROGRAMMER: ON MOBILE DEVICES, ROTATE RANDOMLY FOR EACH RESPONDENT, VERTICAL ORIENTATION AND SEPARATE INTO SCREENS WITH TWO OR THREE ITEMS PER SCREEN].

		Unfavourable feelings				No feelings				Favourable feelings
**p11f_2**	PP voters	0	15	30	40	50	60	70	85	100
**p11g_2**	PSOE voters	0	15	30	40	50	60	70	85	100
**p11h_2**	Ciudadanos voters	0	15	30	40	50	60	70	85	100
**p11i_2**	Unidas Podemos voters	0	15	30	40	50	60	70	85	100
**p11r_2**	Vox voters	0	15	30	40	50	60	70	85	100

[LEADERSHIP GROUP]

And what are your feelings about these leaders?

[PROGRAMMER: ON MOBILE DEVICES, RANDOMLY ROTATE ORDER FOR EACH RESPONDENT, VERTICAL ORIENTATION AND SEPARATE INTO SCREENS WITH TWO OR THREE ITEMS PER SCREEN].

		Unfavourable feelings				No feelings				Favourable feelings
**p11j_2**	Pablo Casado	0	15	30	40	50	60	70	85	100
**p11k_2**	Pedro Sanchez	0	15	30	40	50	60	70	85	100
**p11l_2**	Albert Rivera	0	15	30	40	50	60	70	85	100
**p11m_2**	Pablo Iglesias	0	15	30	40	50	60	70	85	100
**p11n_2**	Iñigo Urkullu	0	15	30	40	50	60	70	85	100
**p11o_2**	Carles Puigdemont	0	15	30	40	50	60	70	85	100
**p11p_2**	Oriol Junqueras	0	15	30	40	50	60	70	85	100
**P11q_2**	Santiago abascal	0	15	30	40	50	60	70	85	100

Now indicate how much you trust various groups of people. For each, indicate the extent to which you trust the people in that group on a scale of 0 to 10.

[PROGRAMMER: ROTATE THE ORDER OF QUESTIONS AT RANDOM FOR EACH RESPONDENT]

[PROGRAMMER: ON MOBILE DEVICES, ROTATE RANDOMLY FOR EACH RESPONDENT, VERTICAL ORIENTATION AND SEPARATE INTO SCREENS WITH TWO OR THREE ITEMS PER SCREEN].

		Don't trust at all										I completely trust
**p13a_2**	Your family	0	1	2	3	4	5	6	7	8	9	10
**p13b_2**	Your neighbours	0	1	2	3	4	5	6	7	8	9	10
**p13c_2**	People you know personally	0	1	2	3	4	5	6	7	8	9	10
**p13d_2**	People you meet for the first time	0	1	2	3	4	5	6	7	8	9	10
**p13e_2**	People of another religion	0	1	2	3	4	5	6	7	8	9	10
**p13f_2**	People of other nationalities	0	1	2	3	4	5	6	7	8	9	10
**p13g_2**	The Catalans	0	1	2	3	4	5	6	7	8	9	10
**p13h_2**	The Basques	0	1	2	3	4	5	6	7	8	9	10
**p13i_2**	The people of Madrid	0	1	2	3	4	5	6	7	8	9	10
**p13j_2**	The Andalusians	0	1	2	3	4	5	6	7	8	9	10
**p13k_2**	Immigrants residing in our country	0	1	2	3	4	5	6	7	8	9	10

FOR QUESTIONS p13A_2 TO p13K_2 PROGRAMMER: DON'T KNOW...888 (AUTOMATICALLY GENERATED IF RESPONDENT SKIPS WITHOUT ANSWERING AND AFTER INSISTING)

PROGRAMMER: DON'T KNOW...888 (GENERATED AUTOMATICALLY IF RESPONDENT SKIPS WITHOUT ANSWERING AND AFTER INSISTING)

FOR QUESTIONS p13A_2 TO p13J_2 PROGRAMMER: IF RESPODENT MOVES FORWARD WITHOUT ANSWERING, PLEASE PRESENT A MESSAGE THAT SAYS "IF YOU GO FORWARD WITHOUT ANSWERING ONE OF THE QUESTIONS ON THIS SCREEN, YOUR ANSWER FOR THAT QUESTION WILL BE RECORDED AS "DON'T KNOW / DON'T ANSWER" FOR THAT QUESTION, DO YOU AGREE? AND PRESENT RESPONSE OPTIONS "YES" AND "NO".

How much do you trust various political groups? For each, indicate the extent to which you trust the people in that group on a scale of 0 to 10.

[PROGRAMMER: RANDOMLY ROTATE THE ORDER OF QUESTIONS FOR EACH RESPONDENT]

[PROGRAMMER: ON MOBILE DEVICES, RANDOMLY ROTATE ORDER FOR EACH RESPONDENT, VERTICAL ORIENTATION AND SEPARATE INTO SCREENS WITH TWO OR THREE ITEMS PER SCREEN].

		I don't trust at all.										I completely trust
**p14a_2**	PP voters	0	1	2	3	4	5	6	7	8	9	10
**p14b_2**	PSOE voters	0	1	2	3	4	5	6	7	8	9	10
**p14c_2**	Ciudadanos voters	0	1	2	3	4	5	6	7	8	9	10
**p14d_2**	Podemos and IU voters	0	1	2	3	4	5	6	7	8	9	10
**p14e1**	ERC voters	0	1	2	3	4	5	6	7	8	9	10
**p14f_2**	PDeCAT voters	0	1	2	3	4	5	6	7	8	9	10
**p14g_2**	EAJ-PNV voters	0	1	2	3	4	5	6	7	8	9	10
**p14h_2**	Vox voters	0	1	2	3	4	5	6	7	8	9	10

FOR QUESTIONS p14A_2 TO p14H_2 PROGRAMMER: DON'T KNOW...888 (GENERATED AUTOMATICALLY IF RESPONDENT SKIPS WITHOUT ANSWERING AND AFTER INSISTING)

FOR QUESTIONS p14A_2 TO p14H_2 PROGRAMMER: IF RESPONDENT MOVES FORWARD WITHOUT ANSWERING, PLEASE PRESENT A MESSAGE THAT SAYS "IF YOU MOVE FORWARD WITHOUT ANSWERING ONE OF THE QUESTIONS ON THIS SCREEN, YOUR ANSWER FOR THAT QUESTION WILL BE RECORDED AS "DON'T KNOW / NO RESPONSE", DO YOU AGREE? WITH RESPONSE OPTIONS "YES"

IMPORTANT: ON MOBILE DEVICES ORIENTATION MUST BE HORIZONTAL

[PROGRAMMER: ROTATE QUESTIONS RANDOMLY AND PRESENT THEM ON SEPARATE SCREENS]

[IMPORTANT: ON MOBILE DEVICES ORIENTATION MUST BE HORIZONTAL]

Could you please tell us on a scale of 1 to 7, where 1 is "I don't trust at all" and 7 is "I trust completely", how much you trust each of the following political institutions...

[PROGRAMMER: PUT ON NEW SCREEN]

trust1a_2 ...the Spanish Parliament

I don't trust at all						I trust completely
1	2	3	4	5	6	7

trust1b_2 ...the Spanish government

[PROGRAMMER: PUT ON NEW SCREEN]

I don't trust at all						I trust completely
1	2	3	4	5	6	7

trust1c_2 ...the Parliament of **[PROGRAMMER: NAME OF AUTONOMOUS COMMUNITY]**

[PROGRAMMER: PUT ON NEW SCREEN]

I don't trust at all						I trust completely
1	2	3	4	5	6	7

trust1d_2 ...the government of **[PROGRAMMER: NAME OF AUTONOMOUS COMMUNITY]**

[PROGRAMMER: PUT ON NEW SCREEN]

I don't trust at all						I trust completely
1	2	3	4	5	6	7

trust1e_2 ...politicians in Spain

[PROGRAMMER: PUT ON NEW SCREEN]

I don't trust at all						I trust completely
1	2	3	4	5	6	7

trust1f_2 ...political parties in Spain

[PROGRAMMER: PUT ON NEW SCREEN]

I don't trust at all						I trust completely
1	2	3	4	5	6	7

trust1g_2 ...the Spanish police

[PROGRAMMER: PUT ON NEW SCREEN]

I don't trust at all						I trust completely
1	2	3	4	5	6	7

trust1h_2 ...the Spanish judicial system

[PROGRAMMER: PUT ON NEW SCREEN]

I don't trust at all						I trust completely
1	2	3	4	5	6	7

trust1i_2 ...the European Parliament

[PROGRAMMER: PUT ON NEW SCREEN]

I don't trust at all						I trust completely
1	2	3	4	5	6	7

trust1j_2 ...the government of the European Union (The European Commission)

[PROGRAMMER: PUT ON NEW SCREEN]

I don't trust at all						I trust completely
1	2	3	4	5	6	7

END OF EXPERIMENT

---

p12a_2 Would you say that, in general, you can trust most people or that you can never be too careful in dealing with others? Please place yourself on the following scale of 0 to 10.

[PROGRAMMER: ON MOBILE DEVICES, VERTICAL ORIENTATION]

You can never be too careful										Most people can be trusted
0	1	2	3	4	5	6	7	8	9	10

p12b_2 And do you think that most people would try to take advantage of you if they could, or that they would be honest with you?

[PROGRAMMER: ON MOBILE DEVICES, VERTICAL ORIENTATION]

Most people would try take advantage of me										Most of the people would be honest with me
0	1	2	3	4	5	6	7	8	9	10

p12c_2 Would you say that most of the time people try to help others or that they mainly look out for themselves?

[PROGRAMMER: ON MOBILE DEVICES, VERTICAL ORIENTATION]

Most of the time people look out for themselves										Most of the time people try to help others
0	1	2	3	4	5	6	7	8	9	10

Now, pleasure turn your attention to this list of political leaders and for each one, please indicate whether you know of them or not and then how you would assess their political activity on the following scale:

[PROGRAMMER: RANDOMLY ROTATE ORDER OF QUESTIONS]

p40a_2 Pablo Casado

1 I know of him
2 I don't know of him **[PROGRAMMER: GO TO NEXT CANDIDATE AND THE NEXT QUESTION IS 999 MISSING]**

p41a_2

[PROGRAMMER: VERTICAL ORIENTATION ON MOBILE DEVICES]

Very badly										Very good
0	1	2	3	4	5	6	7	8	9	10

p40b_2 Pedro Sánchez

1 I know of him
2 I don't know of him **[PROGRAMMER: GO TO NEXT CANDIDATE AND THE NEXT QUESTION IS 999 MISSING]**

p41b_2

[PROGRAMMER: VERTICAL ORIENTATION ON MOBILE DEVICES]

Very badly										Very good
0	1	2	3	4	5	6	7	8	9	10

p40c_2 Albert Rivera

1 I know of him
2 I don't know of him **[PROGRAMMER: GO TO NEXT CANDIDATE AND THE NEXT QUESTION IS 999 MISSING]**

p41c_2

[PROGRAMMER: VERTICAL ORIENTATION ON MOBILE DEVICES]

Very badly										Very good
0	1	2	3	4	5	6	7	8	9	10

p40d_2 Pablo Iglesias

1 I know of him
2 I don't know of him **[PROGRAMMER: GO TO NEXT CANDIDATE AND THE NEXT QUESTION IS 999 MISSING]**

p41d_2

[PROGRAMMER: VERTICAL ORIENTATION ON MOBILE DEVICES]

Very badly										Very good
0	1	2	3	4	5	6	7	8	9	10

p40e_2 Alberto Garzón

1 I know of him
2 I don't know of him **[PROGRAMMER: GO TO NEXT CANDIDATE AND THE NEXT QUESTION IS 999 MISSING]**

p41e_2

[PROGRAMMER: VERTICAL ORIENTATION ON MOBILE DEVICES]

Very badly										Very good
0	1	2	3	4	5	6	7	8	9	10

p40f_2 Iñigo Urkullu [PROGRAMMER: ASK ONLY IN THE BASQUE COUNTRY]

1 I know of him
2 I don't know of him **[PROGRAMMER: GO TO NEXT CANDIDATE AND THE NEXT QUESTION IS 999 MISSING]**

p41f_2

[PROGRAMMER: VERTICAL ORIENTATION ON MOBILE DEVICES]

Very badly										Very good
0	1	2	3	4	5	6	7	8	9	10

p40g_2 Carles Puigdemont [PROGRAMMER: ASK ONLY IN CATALONIA]

1 I know of him
2 I don't know of him **[PROGRAMMER: GO TO NEXT CANDIDATE AND THE NEXT QUESTION IS 999 MISSING]**

p41g_2

[PROGRAMMER: VERTICAL ORIENTATION ON MOBILE DEVICES]

Very badly										Very good
0	1	2	3	4	5	6	7	8	9	10

p40h_2 Oriol Junqueras [PROGRAMMER: ASK ONLY IN CATALONIA]

1 I know of him
2 I don't know of him **[PROGRAMMER: GO TO NEXT CANDIDATE AND THE NEXT QUESTION IS 999 MISSING]**

p41h_2

[PROGRAMMER: VERTICAL ORIENTATION ON MOBILE DEVICES]

Very badly										Very good
0	1	2	3	4	5	6	7	8	9	10

We all feel more or less connected to the territory or political community (town, city, region, etc.) in which we live, but some of us feel more connected to some places than others. To what extent do you identify with the following localities?

p15a_2 The town or city where I live

[PROGRAMMER: ON MOBILE DEVICES, VERTICAL ORIENTATION]

Do not identify at all										Identify strongly
0	1	2	3	4	5	6	7	8	9	10

p15b_2 Region or autonomous community where I live

[PROGRAMMER: ON MOBILE DEVICES, VERTICAL ORIENTATION]

Do not identify at all										Identify strongly
0	1	2	3	4	5	6	7	8	9	10

p15c_2 Spain

[PROGRAMMER: ON MOBILE DEVICES, VERTICAL ORIENTATION]

Do not identify at all										Identify strongly
0	1	2	3	4	5	6	7	8	9	10

p15d_2 Europe

[PROGRAMMER: ON MOBILE DEVICES, VERTICAL ORIENTATION]

Do not identify at all										Identify strongly
0	1	2	3	4	5	6	7	8	9	10

[PROGRAMMER: GO TO NEXT CANDIDATE and the next question is 999 missing]

PROGRAMMER: IF RESPONDENT ADVANCEA WITHOUT ANSWERING, PRESENT A MESSAGE THAT SAYS "If you advance without answering this question, your answer will be recorded as "Don't know / Don't answer", do you agree? With response options "Yes" and "No".

Now, indicate through what means and how often you are keep informed about current issues. Keep in mind the importance of reading the questions carefully and choosing the answer that best fits your thoughts and opinions. The results and quality of this international research depend on your effort and attention to your responses.

Could you please say how often you keep yourself informed about current political issues, news or opinions through…

[PROGRAMMER: RANDOMLY ROTATE THE ORDER OF ITEMS FOR EACH RESPONDENT]

[PROGRAMMER: ON MOBILE DEVICES, VERTICAL ORIENTATION]

		Never	Less than once a month	Once a month	Several times a month	Once a week	Several times a week	Every day	Several times a day
**p17a_2**	Newspapers	0	1	2	3	4	5	7	8
**p17b_2**	Radio	0	1	2	3	4	5	7	8
**p17c_2**	Magazines	0	1	2	3	4	5	7	8
**p17d_2**	Television	0	1	2	3	4	5	7	8
**p17e_2**	Social networkS	0	1	2	3	4	5	7	8

p26a_2 How often do you discuss politics or political issues with family, friends, colleagues or acquaintances?

1 Less than once a month
2 Once a month
3 Several times a month
4 Once a week
5 Several times a week
6 Every day
0 Never [**GO to p19a_2]**
[PROGRAMMER: IN THIS CASE AUTOMATICALLY CODE 999 FOR QUESTIONS p27a_2, p28a_2 and p29a_2]

p27a_2 How often do you agree with the views of the people with whom you talk about politics?

3 Always
2 Many times
1 Sometimes
0 Never
888 Doesn't Know/No Answer

p28a_2 And how often do you disagree with the views of the people with whom you talk about politics?

3 Always
2 Many times
1 Sometimes
0 Never
888 Doesn't Know/No Answer

p29a_2 Do you think the people you talk to about politics...?

3 They support the same party
2 They divide their support among different parties
1 Support a different party
0 They do not support any party
888 Doesn't Know/No Answer

Do you have an account on one of the following social networks?

		Yes	No
**p19a_2**	Twitter	1	0
**p19b_2**	Facebook	1	0
**p19c_2**	Google +	1	0
**p19d_2**	Linkedin	1	0
**p19e_2**	Instagram	1	0
**p19f_2**	Flickr	1	0
**p19g_2**	Youtube	1	0
**p19h_2**	Other _______	1	0

[PROGRAMMER: IF INTERVIEWED SAYS "NO" ON p19a_2 GO TO p26b_2]

p42_2 We would like to ask you if it is possible to contact you through your Twitter account. All information from such interaction will be treated anonymously and always in conjunction with hundreds of other interviewees for research purposes.

Would you be willing to give us the account contact information:

1. No
2. Yes. Account:

PROGRAMMER: CREATE A VARIABLE CALLED "TWITTER" THAT CONTAINS THE ACCOUNT HANDLE FOR THOSE WHO SAY YES. THIS VARIABLE IS VALUED 999 FOR THOSE WHO SAY NO ON p19a_2 or p42_2

p26b_2 How often do you discuss politics or current political issues on social networks, Facebook, Twitter or other networks?

[PROGRAMMER: ASK ONLY THOSE WHO ANSWERED 1/YES ON ONE OF THE p19a_2-p19h_2; PUT 999 IF THEY SAY NO TO ALL OF THEM]

1 Less than once a month
2 Once a month
3 Several times a month
4 Once a week
5 Several times a week
6 Every day
0 Never [**GO to p30_2**]

[PROGRAMMER: IN THIS CASE GENERATE AUTOMATICALLY 999 FOR QUESTIONS p27b_2 TO p29b_2]

p27b_2 How often do you agree with the views of the people with whom you talk to about politics in these forums?

3 Always
2 Many times
1 Sometimes
0 Never
888 don't know don't answer

p28b_2 Also, how often do you disagree with the views of the people with whom you talk about politics in these forums?

3 Always
2 Many times
1 Sometimes
0 Never
888 don't know don't answer

p29b_2 Do you think that the people you talk to about politics in these forums...

3 They support the same party
2 They divide their support among different parties
1 Support a different party
0 They do not support any party
888 don't know/don't answer

Now you will talk about some aspects of your political preferences. Again, remember the importance of reading the questions carefully and choosing the answer that best fits your thoughts and opinions. The results and quality of this international research depend on your efforts and attention to detail.

There are many ways to try to make things better in Spain, or at least prevent them from getting worse. In the last 12 months have you done any of the following?

[PROGRAMMER: ROTATE THE ORDER OF QUESTIONS AT RANDOM FOR EACH RESPONDENT]

		Yes	No	I don't remember
**p32a_2**	You signed a petition in a signature collection campaign	1	0	2
**p32b_2**	You have boycotted or stopped buying certain products	1	0	2
**p32c_2**	You have worn or shown any campaign badges or stickers	1	0	2
**p32d_2**	You have participated in authorized demonstrations	1	0	2
**p32e_2**	You've participated in political rallies	1	0	2
**P32f_2**	You have contacted a politician or a state, regional or local authority or official	1	0	2
**p32g_2**	You have contacted or appeared in the media to express your opinions	1	0	2

FOR QUESTIONS p32a_2 to p32g_2 PROGRAMMER: RESPONDENTS MUST ANSWER THIS QUESTION

And, in the last 12 months, have you performed any of the following activities on the Internet?

[PROGRAMMER: ROTATE THE ORDER OF QUESTIONS AT RANDOM FOR EACH RESPONDENT]

		Yes	No	I don't remember
**p33a_2**	You have visited the website of a party/candidate/political group	1	0	2

**p33b_2**	You followed a party/candidate/political group on Facebook	1	0	2
**p33c_2**	You have sent an email to a party/candidate/political group	1	0	2
**p33d_2**	You have sent a tweet to a party/candidate/political group on Twitter	1	0	2
**p33e_2**	You have published political news/messages on social networks such as Facebook or Twitter	1	0	2
**p33f_2**	You have debated politics in a political forum or blogged	1	0	2
**p33g_2**	You signed a petition in a web-based signature campaign	1	0	2
**p33h_2**	You have used the Internet to encourage people to vote in elections	1	0	2
**p33i_2**	You have tried to convince people to vote for a candidate/party by using social media	1	0	2
**p33j_2**	You've tried to convince people to vote for a candidate/party using emails	1	0	2
**p33k_2**	You participated in a political event to which you were invited via the Internet	1	0	2
**p33l_2**	You have participated in viral campaigns based on irony or political memes on Twitter or Facebook	1	0	2

For questions p33a_2 to p33l_2 PROGRAMMER: IN THIS QUESTION RESPONDENT MUST REPLY AS THEY HAVE "I DON'T REMEMBER" AS AN OPTION

And, again in the last 12 months, have you done any of the following activities on social networks?

[PROGRAMMER: RANDOMLY ROTATE THE ORDER OF THE ITEMS FOR EACHRESPONDENT. ASK ONLY THOSE WHO ANSWERED 1/YES ON ONE OF THE p19a_2-p19h_2; RECORD 999 FOR ALL OTHERS]

		Yes	No	I don't remember
**p34a_2**	You've followed someone or added friends with different political views	1	0	2
**p34b_2**	You've marked "like" for a political commentary or a tweet posted by others	1	0	2
**p34c_2**	You've shared a political message or tweet posted by others	1	0	2
**p34d_2**	You have responded with a positive comment to a political tweet or Facebook status posted by others	1	0	2
**p34e_2**	You have responded with a negative comment to a political tweet or Facebook status posted by others	1	0	2
**p34f_2**	You've stopped following, blocked or banned someone from your own contacts for political reasons	1	0	2
**p34g_2**	You decided not to publish political content because you were afraid of offending other people	1	0	2
**p34h_2**	You decided not to publish political content because you feared public exposure	1	0	2
**p34i_2**	You've changed your mind after participating in political discussions on Twitter or Facebook	1	0	2
**p34j_2**	You have increased your participation in a political cause after participating in or reading a debate on social networks	1	0	2
**p34k_2**	You have decreased your participation in a political cause after participating in or reading a debate on social networks	1	0	

For questions p34a_2 to p34k_2 PROGRAMMER: PROGRAMMER: FOR THIS QUESTION RESPONDENT MUST REPLY AS THEY HAVE "I DON'T REMEMBER" AS AN OPTION

p35_2 Do you consider yourself close to any political party?

1 Yes **[GO TO p35a_2]**
0 No [**GO TO s8_2]**

p35a_2 Which one?

[PROGRAMMER: ASK IF 1 ON p35_2. RECORD 999 FOR OTHERS]

1 PP (Popular Party)
2 PSOE (Spanish Socialist Workers' Party)
3 Podemos and other affiliated municipal lists (En Comù Podem, En Marea, Ahora Madrid)
4 4 IU (United Left)
5 Ciudadanos (C's - Ciutadans)
6 ERC (Esquerra Republicana de Catalunya)
7 PDeCAT (Partit Demòcrata Europeu Català)
8 EAJ - PNV (Euzko Alderdi Jeltzalea - Basque Nationalist Party)
9 EH - Bildu (Euskal Herria - Bildu)
10 BNG (Bloque Nacionalista Galego)
11 CC (Canary Islands Coalition)
13 VOX
12 Others

p35b_2 And how close do you feel to this party?

[PROGRAMMER: ASK IF 1 ON p35_2. RECORD 999 FOR OTHERS]

3 Very close
2 Somewhat close
1 Not very close
0 Not at all close

p35c_2 Is it important for you to be a [PROGRAMMER: PLACE CHOSEN PARTY NAME FROM p35a_2]? [PROGRAMMER: ASK IF 1 ON p35_2. PUT 999 FOR OTHERS]

3 Extremely important
2 Very important
1 Not very important
0 Nothing important

p35d_2 How well does the word supporters [PROGRAMMER: PLACE CHOSEN PARTY NAME FROM p35a_2] describe you?

[PROGRAMMER: ASK IF 1 ON p35_2. PUT 999 FOR OTHERS]

3 Extremely well
2 Fairly well
1 Not very well
0 Nothing

p35e_2 When you talk about [PROGRAMMER: PLACE CHOSEN PARTY NAME FROM p35a_2], how often do you use the word "we"?

3 Always
2 Most of the time
1 Sometimes
0 Never

[PROGRAMER: ASK IF 1 ON p35_2. RECORD 999 FOR OTHERS]

p35f_2 To what extent do you consider yourself "one" of the [PUT PARTY NAME FROM p35a_2]?

[PROGRAMER: ASK IF 1 ON p35_2. RECORD 999 FOR OTHERS]

3 A lot
2 Some
1 Little
0 Not at all

s8_2 And which best describes your situation in the last seven days? Please choose only one of the following options.

1 Employed, but on temporary leave (includes temporary maternity/paternity leave, accident, illness or holidays).
2. Employed, self-employed, or in a family business
2 Studying, even if you have been on holiday (includes company paid training)
3 Unemployed and actively seeking work
4 Unemployed, wanting to find a job but not actively looking for one
5 With chronic illness or permanent disability
6 Retired
7 Homemaker, stay-at-home parent, or caregiver

s9_2 Which of the statements below best describes how you feel about your current household income?

1 With our current income we live comfortably
2 With our current income we get by
3 With our current income we have difficulties
4 With our current income we have many difficulties

s10_2 Have you been fired from your primary employment at any time in the past year?

1 Yes
2 No

Currently, to what extent do you feel concerned about...

[PROGRAMMER: ROTATE ORDER RANDOMLY FOR EACH RESPONDENT]

		Not at all concerned	Not too concerned	Quite concerned	Very concerned
**s11a_2**	Paying your household bills	0	1	2	3
**s11b_2**	Having to reduce your standard of living	0	1	2	3
**s11c_2**	Having a job	0	1	2	3
**s11d_2**	Paying off loans from the bank or paying mortgage bills	0	1	2	3
