# Supplementary material for: Political and affective polarisation in a democracy in crisis: The E-Dem panel survey dataset (Spain, 2018–2019)
Source: Data Brief. 2020 Jul 23;32:106059. doi: 10.1016/j.dib.2020.106059 (PMC7451797; doi:10.1016/j.dib.2020.106059)
Supplement: Supplementary file 4 [file mmc4.pdf]

Online political participation and deliberation in a democracy in crisis

Wave III Questionnaire

March 2019

Project: *Online Political Participation and Deliberation in a Democracy in Crisis: A New Methodological Approach (E-Dem) (2017-2020)*

PI: Mariano Torcal. Ministry of Economy and Competitiveness, State Programme for the Promotion of Scientific and Technical Research of Excellence, 2017. Ref: CSO2016-79772-P.

Universitat Pompeu Fabra Barcelona

RECSM Research and Expertise Centre for Survey Methodology

[General instructions]

1. Do not force the panellist to answer all the questions

2. Allow them not to answer the questions by moving on to the next one, although a warning message must be issued where they have to confirm their choice every 4 questions

3. In some important questions the message applies

4. Some knowledge questions include the category don't know or prefer not to answer

5. In the data file, the name of variables must appear exactly as in the questionnaire.

6. It is also important to start the questionnaire with a short introduction:

This survey aims to provide the data necessary to analyse aggregate opinions on current issues such as immigration or the pension system, as well as on political trends. It is a study led by researchers from the Pompeu Fabra University, within a European research project on online political participation and deliberation. All the information you and other respondents provide will be shared with that University anonymously and used only for the research purposes mentioned above. If you wish to exercise your right to data protection, you may write to the Department of Political Science at Pompeu Fabra University, located at c/ Ramon Trias Fargas, 25 -27, 08005 Barcelona (Edificio Jaume I - Campus Ciutadella) or to the following e-mail address: departament.cpis@upf.edu.

Below we ask you to confirm if you would like to participate in this interesting survey:

- ○ Yes, I want to participate
- ○ No, I'd rather not participate

Second screen:

You may not remember, a few weeks ago you participated in a survey of a study designed by a group of national and international researchers led by a professor at the Pompeu Fabra University in Barcelona who is interested in studying the opinion of people like you on issues related to our political system and our society. To this end, they would like to count on your participation in another survey that will last approximately 20-25 minutes. In this way you will be part of the same 2,500 people from all over Spain who also participated in the previous one.
Your answers are very important and will help to know the opinions of the Spanish people about the political current situation and the functioning of the democracy in the country. The problems and challenges of our political and social systems require a good study of citizens' opinions and therefore your attentive and dedicated participation is essential. In any case, as with the previous survey, your answers will remain in total anonymity and will only be subject to statistical analysis together with the other hundreds of participants.

We'd like to start by asking you this:

p19a_3 Do you have a twitter account?

1. Yes. **[PROGRAMMER: GO TO QUESTION pi17e_3]**
2. No. **[PROGRAMMER: SKIP TO QUESTION 1 OF THE GENERAL QUESTIONNAIRE p1_3]**

p19a1_3 Are you following the election campaign at all on Twitter?

---

1. Yes. [PROGRAMMER: GO TO THE EXPERIMENT]
2. No. [PROGRAMMER: SKIP TO QUESTION 1 OF THE GENERAL QUESTIONNAIRE p1_3]

EXPERIMENT:

PROGRAMMER: ONLY FOR THOSE WHO CLAIM TO HAVE A TWITTER ACCOUNT AND FOLLOW THE INFORMATION ABOUT THE CAMPAIGN ON THIS CHANNEL. FOR THE REST, ALL THESE QUESTIONS UNTIL p1_3 SHOULD BE CODED AS 999. THOSE NOT SELECTED FOR THE EXPERIMENT HAVE FOR ALL QUESTIONS IN THIS BLOCK A RESPONSE CODE OF 999

We would like to be able to provide you with information about some of the discussions that are taking place among voters regarding these elections. To do this, we will invite you to explore one or more of the following accounts.

With the link below you can enter this or any other Twitter account you want, read what is being discussed and even participate in it if you want to. We leave that to your choice. We will give you approximately three days to do so. After this period of time, you will receive another invitation to return to the survey.

PABLO CASADO: https://twitter.com/pablocasado

PEDRO SANCHEZ: https://twitter.com/sanchezcastejon

PABLO IGLESIAS: https://twitter.com/Pablo_Iglesias_

ALBERT RIVERA: https://twitter.com/Albert_Rivera

CARLES PUIGDEMONT: https://twitter.com/krls

SANTIAGO ABASCAL: https://twitter.com/Santi_ABASCAL

esm0a_3. Would you like to participate in this experiment?

We remind you that your answers are very important and will help to know the opinions of the Spanish people about the current political situation and the functioning of the democracy in the country. The problems and challenges of our political and social systems require a high-quality study of citizens' opinion and, therefore, your attentive and dedicated participation is essential. In any case, as with the previous survey, your answers will remain in total anonymity and they will only be subject to statistical analysis together with hundreds of other participants.

1. **Yes I want to participate → [PROGRAMMER: PROVIDE MESSAGE: "YOU WILL BE CONTACTED AFTER 3 DAYS)]**
2. **I do not want to participate → [PROGRAMMER: SKIP TO p1_3. QUESTIONS PRECEDING IT (THOSE WITH esm_ PREFIX SHOULD BE CODED AS 999]**

PROGRAMMER: AFTER THREE DAYS, ONLY THOSE SELECTED FOR THE EXPERIMENT; THAT IS, THOSE WHO ANSWERED "YES" TO esm0a_3, p19a_3 and p19a1_3 ARE RECONTACTED AFTER THE THREE DAYS AND THE QUESTIONNAIRE STARTS HERE

[EXPERIMENT FILTER QUESTIONS TO BE CARRIED OUT AFTER THREE DAYS]

A few days ago we asked you to follow some of the politicians' accounts on the social network Twitter.

esmP1_3. To begin with, could you tell me if you finally did that?

1. Yes [**PROGRAMMER: CONTINUE WITH esmP2_3]**

2. No **PROGRAMMER:** SKIP TO p1_3. QUESTIONS PRECEDING IT (THOSE WITH esm_ PREFIX SHOULD BE CODED AS 999]

esmP2_3 Would you mind telling me which account or accounts you have followed in these three days? Please check all those you have followed.

Pablo Casado (1)	Pedro Sanchez (2)	Pablo Iglesias (3)	Albert Rivera (4)	Santiago Abascal (5)	Carles Puigdemont (6)

esmP3_3 Did you follow them before we asked you to do so or did you only begin to follow them for the survey?

1. I followed them before [**GO TO esmP4a_3**]
2. I have followed them only now
3. I was following some and I've followed some new ones as a result of this survey

PROGRAMMER: ASK ONLY THOSE WHO HAVE RESPONDED 2 AND 3. THE REST ARE CODED AS 999

esmP3_3 Could you tell me which new account/s you have just begun to follow these last days?

Pablo Casado (1)	Pedro Sanchez (2)	Pablo Iglesias (3)	Albert Rivera (4)	Santiago Abascal (5)	Carles Puigdemont (6)

As you may have noticed, the politicians are very active in this social network. Could you tell me, for the politicians you have followed, how many tweets or messages they have launched during these days that you have followed them?

[PROGRAMMER: ROTATE THE LIST OF CANDIDATES.
SHOW ONLY THE LIST OF CANDIDATES SIGNALLED IN esmP2_3]

	1 per day	Between 1 and 4 times a day	Between 5 and 10 times a day	More than 10 times	I don't know
**esmP4a_3** Pablo Casado	1	2	3	4	8
**esmP4b_3** Pedro Sanchez	1	2	3	4	8
**esmP4c_3** Pablo Iglesias	1	2	3	4	8
**esmP4d_3** Albert Rivera	1	2	3	4	8
**esmP4e _3**Santia go	1	2	3	4	8
**esmP4f_3** Carles Puigdemont	1	2	3	4	8

The messages posted on this social network can have different content. In general, what types of tweets do you think these candidates tend to publish?

[PROGRAMMER: MULTIPLE ANSWER]

	Text only	Images	Videos	Links to press articles	I don't know
**esmP5a_3** Pablo Casado	1	2	3	4	8
**esmP5b_3** Pedro Sanchez	1	2	3	4	8
**esmP5c_3** Pablo Iglesias	1	2	3	4	8
**esmP5d_3** Albert Rivera	1	2	3	4	8
**esmP5e_3** Santiago Abascal	1	2	3	4	8
**esmP5f_3** Carles Puigdemont	1	2	3	4	8

Finally, could you tell me with which politicians you would associate the following hashtags? (Slogans or phrases that begin with the character # on Twitter) (You can only associate one with each candidate)

	**#FreeTothom**	**#EspañaLoPrimero**	**#SoyLiberal**	**#SíSePuede**	**#LaEspañaQueQuieres**	**#ValorSeguro**	I don't know
**esmP6a_3** Pablo Casado	1	2	3	4	5	6	8
**esmP6b_3** Pedro Sanchez	1	2	3	4	5	6	8
**esmP6c_3** Pablo Iglesias	1	2	3	4	5	6	8
**esmP6d_3** Albert Rivera	1	2	3	4	5	6	8
**esmP6e_3** Santiago Abascal	1	2	3	4	5	6	8
**esmP6f_3** Carles Puigdemont	1	2	3	4	5	6	8

[PROGRAMMER: THIS LAST QUESTION IS SUBJECT TO CHANGE ONCE THE CAMPAIGN STARTS, SO IT IS IMPORTANT TO KNOW WHEN THE SURVEY IS GOING TO BE LAUNCHED IN ORDER TO UPDATE IT OR CHECK THAT IT IS STILL VALID]

[PROGRAMMER: START OF QUESTIONNAIRE FOR ALL OTHER RESPONDENTS (THOSE NOT SELECTED FOR THE EXPERIMENT START HERE AFTER esmP6f_3 AND HAVE RESPONSE CODE 999 FOR ALL QUESTIONS esm_)]

p1_3 To begin with, to what extent are you interested in politics? A lot, a fair amount, a little or not at all?

1 A lot
2 A fair amount
3 A little
4 Not at all

p2_3 To what extent are you satisfied with the general economic situation in Spain? Please indicate your answer on a scale from 0 to 10 where 0 is "Completely dissatisfied" and 10 is "Completely satisfied".

[PROGRAMMER: VERTICAL ORIENTATION ON MOBILE DEVICES]

**Completely dissatisfied**										**Completely satisfied**
0	1	2	3	4	5	6	7	8	9	10

p47_3 To what extent are you satisfied with the general political situation in Spain? Please indicate your answer on a scale from 0 to 10 where 0 is "Completely dissatisfied" and 10 is "Completely satisfied"

[PROGRAMMER: VERTICAL ORIENTATION ON MOBILE DEVICES]

**Completely dissatisfied**										**Completely satisfied**
0	1	2	3	4	5	6	7	8	9	10

p3_3 In your opinion, what is the main problem that currently exists in Spain? Please choose one of the following options:

[PROGRAMMER: RANDOMLY ROTATE THE ORDER OF THE TOPICS FOR EACH RESPONDENT]

1 Unemployment
2 Drugs
3 The healthcare system
4 Housing
5 Education
6 Domestic ETA terrorism
7 International terrorism (Islamic State/ISIS)
8 Corruption
9 Immigration
10 The Euro
11 Violence against women
12 Political instability
13 The refugee crisis
14 Environmental problems
15 Pensions
16 Citizen insecurity
17 Taxes
18 Parties and politicians in general
20 The situation in Catalonia
21 The economic situation
19 Other________

888 I don't know

In your opinion, how would you rate the situation in Spain with respect to the following issues? Please indicate your answer on a scale from 0 to 10 where 0 is "Very bad" and 10 is "Very good"

[PROGRAMMER: ROTATE THE ORDER OF THE TOPICS AT RANDOM FOR EACH RESPONDENT, SEPARATED INTO SCREENS WITH TWO ITEMS ON EACH FOR A TOTAL OF 3 SCREENS]

[PROGRAMMER: VERTICAL ORIENTATION ON MOBILE DEVICES]

p4a_3 Unemployment

Very bad										Very good
0	1	2	3	4	5	6	7	8	9	10

[PROGRAMMER: DON'T KNOW...888 (GENERATED AUTOMATICALLY IF RESPONDENT SKIPS WITHOUT ANSWERING AND AFTER INSISTING)]

p4b_3 Education

Very bad										Very good
0	1	2	3	4	5	6	7	8	9	10

[PROGRAMMER: DON'T KNOW...888 (GENERATED AUTOMATICALLY IF RESPONDENT SKIPS WITHOUT ANSWERING AND AFTER INSISTING)]

p4c_3 Health

Very bad										Very good
0	1	2	3	4	5	6	7	8	9	10

[PROGRAMMER: DON'T KNOW...888 (GENERATED AUTOMATICALLY IF RESPONDENT SKIPS WITHOUT ANSWERING AND AFTER INSISTING)]

p4d_3 Immigration

Very bad										Very good
0	1	2	3	4	5	6	7	8	9	10

[PROGRAMMER: DON'T KNOW...888 (GENERATED AUTOMATICALLY IF RESPONDENT SKIPS WITHOUT ANSWERING AND AFTER INSISTING)]

p4e_3 The pension system

Very bad										Very good
0	1	2	3	4	5	6	7	8	9	10

[PROGRAMMER: DON'T KNOW...888 (GENERATED AUTOMATICALLY IF RESPONDENT SKIPS WITHOUT ANSWERING AND AFTER INSISTING)]

p4f_3 Corruption

Very bad										Very good
0	1	2	3	4	5	6	7	8	9	10

[PROGRAMMER: DON'T KNOW...888 (GENERATED AUTOMATICALLY IF RESPONDENT SKIPS WITHOUT ANSWERING AND AFTER INSISTING)]

p4g_3 Violence against women

Very bad										Very good
0	1	2	3	4	5	6	7	8	9	10

[PROGRAMMER: DON'T KNOW...888 (GENERATED AUTOMATICALLY IF RESPONDENT SKIPS WITHOUT ANSWERING AND AFTER INSISTING)]

p4h_3 The situation in Catalonia

Very bad										Very good
0	1	2	3	4	5	6	7	8	9	10

[PROGRAMMER: DON'T KNOW...888 (GENERATED AUTOMATICALLY IF RESPONDENT SKIPS WITHOUT ANSWERING AND AFTER INSISTING)]

p37a_3 And how has the economic situation in Spain changed in the last 12 months?

1 It's gotten much worse
2 It's gotten a little worse
3 It's the same
4 It’s gotten a little better
5 It's gotten much better

p37b_3 And how has the economic situation of your household changed in the last 12 months?

1 It's gotten much worse
2 It's gotten a little worse
3 It's the same
4 It’s gotten a little better
5 It's gotten much better

p38a_3 Now, to what extent are you satisfied with the work of the current Spanish government so far? Please indicate your answer on a scale from 0 to 10 where 0 is "Completely dissatisfied" and 10 is "Completely satisfied".

[PROGRAMMER: VERTICAL ORIENTATION ON MOBILE DEVICES]

Completely dissatisfied										Completely satisfied
0	1	2	3	4	5	6	7	8	9	10

p38b_3 Now, to what extent are you satisfied with the work of the PP as the main opposition party so far? Please indicate your answer on a scale from 0 to 10 where 0 is "Completely dissatisfied" and 10 is "Completely satisfied".

[PROGRAMMER: VERTICAL ORIENTATION ON MOBILE DEVICES]

Completely dissatisfied										Completely satisfied
0	1	2	3	4	5	6	7	8	9	10

PROGRAMMER: IN THE FOLLOWING QUESTIONS IF RESPONDENTS GO FORWARD WITHOUT ANSWERING, DISPLAY A MESSAGE THAT SAYS "IF YOU GO FORWARD WITHOUT ANSWERING THIS QUESTION, YOUR ANSWER WILL BE RECORDED AS "I DON'T KNOW / DID NON'T ANSWER" (888). DO YOU AGREE?" WITH RESPONSE OPTIONS "YES" AND "NO".

p48_3 Can you tell me with what level of interest you are following the electoral campaign?

1. With no interest
2. With little interest
3. With some interest
4. With much interest

p49_3 During this electoral campaign, how often do you follow the political and electoral information through the general information newspapers?

1. Every day or almost every day
2. Several days of the week
3. Only on weekends
4. From time to time
5. Never or hardly ever

p50_3 And how often do you follow political and electoral information on television?

1. Every day or almost every day
2. Several days of the week
3. Only on weekends
4. From time to time
5. Never or hardly ever

p51_3 And how often do you follow political and electoral information on the radio?

1. Every day or almost every day
2. Several days of the week
3. Only on weekends
4. From time to time
5. Never or hardly ever

p52_3 And how often do you follow political and electoral information on the Internet?

1. Every day or almost every day
2. Several days of the week
3. Only on weekends
4. From time to time
5. Never or hardly ever **[SKIP to QUESTION p6_3]**

[PROGRAMMER: FOR ALL BUT THOSE WHO RESPONDED, "NEVER OR HARDLY EVER" ON THE PREVIOUS INTERNET QUESTION. FOR THOSE RESPONDENTS, CODE RESPONSE AS 999]

p53_3 To date, which of the following websites have you consulted to obtain political and electoral information? You can choose more than one option.

1. Media pages (newspapers, radio, etc.) [p53a_3]
2. Party/candidate pages [p53b_3]
3. Pages of citizen organizations or civic movements [p53c_3]
4. Blogs and discussion forums [p53d_3]
5. Social networks (Facebook, Tuenti, Twitter, etc.) [p53e_3]
6. Other____________[p53f_3]

888. I don't want to answer [p53g_3]

Now we'll talk about aspects of your ideological preferences. Remember again how important it is that you read the questions carefully and choose the answer that best fits what you think and feel. The results and quality of this international research depend on your effort and attention to answer. We remind you that your answers will remain anonymous and will only be treated, along with those of other respondents, in a statistical manner.

p6_3 When talking about politics, people talk about "left" and "right". Can you please tell us where you would position yourself on a scale of 0 to 10 where 0 means "left" and 10 means "right"?

[PROGRAMMER: VERTICAL ORIENTATION ON MOBILE DEVICES]

Left										Right
0	1	2	3	4	5	6	7	8	9	10

PROGRAMMER: IF RESPONDENT GOES FORWARD WITHOUT ANSWERING, DISPLAY A MESSAGE THAT SAYS "IF YOU WILL ADVANCE WITHOUT ANSWERING THIS QUESTION, YOUR ANSWER WILL BE RECORDED AS "DON'T KNOW / DON'T ANSWER". DO YOU AGREE? WITH RESPONSE OPTIONS OF "YES" AND "NO".

PROGRAMMER: DON'T KNOW...888 (GENERATED AUTOMATICALLY IF RESPONDENTS MOVE ON TO THE NEXT QUESTION WITHOUT ANSWERING AND AFTER INSISTING)

And where would you place each of the following political parties on this same scale?

[PROGRAMMER: RANDOMLY ROTATE THE ORDER OF THE PARTIES FOR EACH RESPONDENT]

[PROGRAMMER: ON MOBILE DEVICES, RANDOMLY ROTATE FOR EACH RESPONDENT, VERTICAL ORIENTATION AND SEPARATE INTO SCREENS WITH TWO OR THREE ITEMS PER SCREEN].

		Left										Right	I don't know
**P7a_3**	PP (People's Party)	0	1	2	3	4	5	6	7	8	9	10	888
**P7b_3**	PSOE (Spanish Socialist Workers' Party)	0	1	2	3	4	5	6	7	8	9	10	888
**P7c_3**	Podemos (En comú podem)	[illegible]	[illegible]	[illegible]	[illegible]	[illegible]	[illegible]	[illegible]	[illegible]	[illegible]	[illegible]	[illegible]	888
**P7e_3**	Ciudadanos (C's - Ciutadans)	0	1	2	3	4	5	6	7	8	9	10	888
**P7f_3**	ERC (Esquerra Republicana de Catalunya)	0	1	2	3	4	5	6	7	8	9	10	888
**P7g_3**	JxCat (Junts per Catalunya)	0	1	2	3	4	5	6	7	8	9	10	888
**P7h_3**	EAJ-PNV (Euzko Alderdi Jeltzalea, Basque Nationalist Party)	0	1	2	3	4	5	6	7	8	9	10	888
**P7i_3**	EH-Bildu (Euskal Herria-Bildu)	0	1	2	3	4	5	6	7	8	9	10	888
**P7n_3**	Coalición Canaria	0	1	2	3	4	5	6	7	8	9	10	888
**P7m_3**	Compromís	0	1	2	3	4	5	6	7	8	9	10	888
**P7o_3**	En Marea	0	1	2	3	4	5	6	7	8	9	10	888
**P7l_3**	Vox	0	1	2	3	4	5	6	7	8	9	10	888

p8_3 Nowadays, the Autonomous Communities can legislate, together with the Government and the National Legislature, on some aspects of the citizens' daily life, such as health and education. However, not everyone considers that this should be the case.

On this subject, could you tell me where you would position yourself on the following scale from 0 to 10?

[PROGRAMMER: VERTICAL ORIENTATION ON MOBILE DEVICES]

The Spanish Government should regain its powers										The Autonomous Communities should be able to legislate on major issues in citizens' daily lives
0	1	2	3	4	5	6	7	8	9	10

And on this same issue, where do you think the following political parties are positioned on the same scale?

[PROGRAMMER: RANDOMLY ROTATE THE ORDER OF THE PARTIES FOR EACH RESPONDENT]

[PROGRAMMER: ON MOBILE DEVICES, RANDOMLY ROTATE ORDER FOR EACH RESPONDENT, VERTICAL ORIENTATION AND SEPARATE INTO SCREENS WITH TWO OR THREE ITEMS PER SCREEN].

		The Spanish Government should regain its powers										The Autonomous Communities should be able to legislate on major issues in citizens' daily lives	I don't know
**P9a_3**	PP (People's Party)	0	1	2	3	4	5	6	7	8	9	10	888
**P9b_3**	PSOE (Spanish Socialist Workers' Party)	0	1	2	3	4	5	6	7	8	9	10	888
**P9c_3**	Podemos (En comú podem)	0	1	2	3	4	5	6	7	8	9	10	888
**P9e_3**	Ciudadanos (C's - Ciutadans)	0	1	2	3	4	5	6	7	8	9	10	888
**P9f_3**	ERC (Esquerra Republicana de Catalunya)	0	1	2	3	4	5	6	7	8	9	10	888
**P9g_3**	JxCat (Junts per Catalunya)	0	1	2	3	4	5	6	7	8	9	10	888
**P9h_3**	EAJ-PNV (Euzko Alderdi Jeltzalea, Basque Nationalist Party)	0	1	2	3	4	5	6	7	8	9	10	888
**P9i_3**	EH-Bildu (Euskal Herria-Bildu)	0	1	2	3	4	5	6	7	8	9	10	888
**P9n_3**	Coalición Canaria	0	1	2	3	4	5	6	7	8	9	10	888
**P9m_3**	Compromís	0	1	2	3	4	5	6	7	8	9	10	888
**P9o_3**	En Marea	0	1	2	3	4	5	6	7	8	9	10	888
**P9l_3**	Vox	0	1	2	3	4	5	6	7	8	9	10	888

Now we would like to know your opinion on some national issues that are the subject of public debate. Please indicate your response on a scale from 0 to 10

[PROGRAMMER: RANDOMLY ROTATE QUESTIONS p10a_3 to p10h_3 FOR EACH RESPONDENT, VERTICAL ORIENTATION ON MOBILE DEVICES]

p10a_3 Generally speaking, would you say that immigrants have to adapt to the customs of Spain and their region or that they should be able to maintain their customs despite living in another country?

They have to adapt to the customs of Spain										They should be able to keep their customs
0	1	2	3	4	5	6	7	8	9	10

p10b_3 And, do you think that private initiative (private companies) or, on the other hand, state intervention is the best way to solve the problems of the Spanish economy?

Private initiative is the best way										State intervention is the best way
0	1	2	3	4	5	6	7	8	9	10

p10c_3 Would you say that same-sex marriages should be prohibited or allowed by law?

They should be forbidden by law										They should be allowed by law
0	1	2	3	4	5	6	7	8	9	10

p10d_3 And, do you think that the main public services should be carried out by private companies or by public institutions of the State?

They should be carried out by private companies										They should be carried out by public institutions
0	1	2	3	4	5	6	7	8	9	10

p10e_3 Would you say that women should have the right to abortion?

Women should not have the right to abortion										Women should have the right to abortion
0	1	2	3	4	5	6	7	8	9	10

p10f_3 Would you say that income and wealth are distributed fairly among regular people in Spain or that wealth should be redistributed more fairly?

Wealth is fairly distributed										Wealth should be redistributed more fairly
0	1	2	3	4	5	6	7	8	9	10

p10g_3 And, do you think a woman should be prepared to give up her job for the sake of her family or should she be able to work?

She should be prepared to quit her job for the sake of her family										She should be able to work
0	1	2	3	4	5	6	7	8	9	10

p10h_3 Would you say that immigration to Spain should be reduced or increased?

Immigration to Spain should be reduced										Immigration to Spain should be increased
0	1	2	3	4	5	6	7	8	9	10

p10i_3 Would you say that the solution for Catalonia…

... is through the rapid application of the Constitutional Article 155										… is through granting the right of self-determination with a referendum
0	1	2	3	4	5	6	7	8	9	10

[PROGRAMMER: FOR QUESTIONS p10A_3 TO p10H_3 PROGRAMMER: DON'T KNOW...888 (GENERATED AUTOMATICALLY IF RESPONDENTS MOVE ON TO THE NEXT QUESTION WITHOUT ANSWERING AND AFTER INSISTING)

FOR QUESTIONS p10A_3 TO p10H_3 PROGRAMMER: IF RESPONDENT GOES FORWARD WITHOUT ANSWERING, DISPLAY A MESSAGE THAT SAYS "IF YOU ADVANCE WITHOUT ANSWERING THIS QUESTION, YOUR ANSWER WILL BE RECORDED AS "DON'T KNOW / DON'T ANSWER". DO YOU AGREE?" AND GIVE RESPONSE OPTIONS "YES" AND "NO".]

We would also like to know your feelings about some groups of people in Spanish society, using this thermometer.

Ratings between 60 and 100 mean that you have rather favourable feelings toward that group of people, with 100 being very favourable; while ratings between 0 and 40 mean instead that you have no favourable feelings toward the group, with 0 being very unfavourable. If you do not have particularly favourable or unfavourable feelings toward a group, you should choose a 50 grade rating.

[PROGRAMMER: ROTATE THE ORDER OF QUESTIONS RANDOMLY WITHIN EACH GROUP]

[SOCIAL GROUP]

[PROGRAMMER: ON MOBILE DEVICES, RANDOMLY ROTATE ORDER FOR EACH RESPONDENT, VERTICAL ORIENTATION AND SEPARATE INTO SCREENS WITH TWO OR THREE ITEMS PER SCREEN].

		Unfavourable feelings				No feelings				Favourable feelings
**p11a_3**	The Basques	0	15	30	40	50	60	70	85	100
**p11b_3**	The Catalans	0	15	30	40	50	60	70	85	100
**p11c_3**	The people of Madrid	0	15	30	40	50	60	70	85	100
**p11d_3**	The Andalusians	0	15	30	40	50	60	70	85	100
**p11e_3**	Refugees	0	15	30	40	50	60	70	85	100
**p11s_3**	Immigrants	0	15	30	40	50	60	70	85	100

[VOTING GROUPS]

And what about these groups of people?

[PROGRAMMER: ON MOBILE DEVICES, RANDOMLY ROTATE ORDER FOR EACH RESPONDENT, VERTICAL ORIENTATION AND SEPARATE INTO SCREENS WITH TWO OR THREE ITEMS PER SCREEN].

		Unfavourable feelings				No feelings				Favourable feelings
**p11f_3**	PP voters	0	15	30	40	50	60	70	85	100
**p11g_3**	PSOE voters	0	15	30	40	50	60	70	85	100
**p11h_3**	Ciudadanos voters	0	15	30	40	50	60	70	85	100
**p11i_3**	Podemos voters	0	15	30	40	50	60	70	85	100
**p11r_3**	Vox voters	0	15	30	40	50	60	70	85	100

[LEADERSHIP GROUP]

And what are your feelings about these leaders?

[PROGRAMMER: ON MOBILE DEVICES, RANDOMLY ROTATE ORDER FOR EACH RESPONDENT, VERTICAL ORIENTATION AND SEPARATE INTO SCREENS WITH TWO OR THREE ITEMS PER SCREEN].

		Unfavourable feelings				No feelings				Favourable feelings
**p11j_3**	Pablo Casado	0	15	30	40	50	60	70	85	100
**p11k_3**	Pedro Sanchez	0	15	30	40	50	60	70	85	100
**p11l_3**	Albert Rivera	0	15	30	40	50	60	70	85	100
**p11m_3**	Pablo Iglesias	0	15	30	40	50	60	70	85	100
**p11n_3**	Iñigo Urkullu	0	15	30	40	50	60	70	85	100
**p11o_3**	Carles Puigdemont	0	15	30	40	50	60	70	85	100
**p11p_3**	Oriol Junqueras	0	15	30	40	50	60	70	85	100
**P11q_3**	Santiago Abascal	0	15	30	40	50	60	70	85	100

Now we would like to know how much you trust various groups of people. For each, indicate the extent to which you trust the people in that group on a scale of 0 to 10.

[PROGRAMMER: RANDOMLY ROTATE THE ORDER OF QUESTIONS FOR EACH RESPONDENT]

[PROGRAMMER: ON MOBILE DEVICES, RANDOMLY ROTATE ORDER FOR EACH RESPONDENT, VERTICAL ORIENTATION AND SEPARATE INTO SCREENS WITH TWO OR THREE ITEMS PER SCREEN].

		I don't trust it at all									I have every confidence
**p13a_3**	Your family	0	1	2	3	4	5	6	7	8	9	10
**p13b_3**	Your neighbours	0	1	2	3	4	5	6	7	8	9	10
**p13c_3**	People you know personally	0	1	2	3	4	5	6	7	8	9	10
**p13d_3**	People you meet for the first time	0	1	2	3	4	5	6	7	8	9	10
**p13e_3**	People of another religion	0	1	2	3	4	5	6	7	8	9	10
**p13f_3**	People of other nationalities	0	1	2	3	4	5	6	7	8	9	10
**p13g_3**	The Catalans	0	1	2	3	4	5	6	7	8	9	10
**p13h_3**	The Basques	0	1	2	3	4	5	6	7	8	9	10
**p13i_3**	People from Madrid	0	1	2	3	4	5	6	7	8	9	10
**p13j_3**	People from Andalusia	0	1	2	3	4	5	6	7	8	9	10
**p13k_3**	Immigrants residing in our country	0	1	2	3	4	5	6	7	8	9	10

FOR QUESTIONS p13a_3 TO p13k_3 [PROGRAMMER: DON'T KNOW...888 (AUTOMATICALLY GENERATED IF RESPONDENTS MOVE ON TO THE NEXT QUESTION WITHOUT ANSWERING AND AFTER INSISTING)]

[PROGRAMMER: DON'T KNOW...888 (GENERATED AUTOMATICALLY IF RESPONDENTS GO FORWARD WITHOUT ANSWERING AND AFTER INSISTING)]

FOR QUESTIONS p13a_3 TO p13j_3 [PROGRAMMER: IF RESPONDENTS GO FORWARD WITHOUT ANSWERING, DISPLAY A MESSAGE THAT SAYS "IF YOU GO FORWARD WITHOUT ANSWERING ONE OF THE QUESTIONS ON THIS SCREEN, YOUR ANSWER FOR THAT QUESTION WILL BE RECORDED AS "DON'T KNOW / DON'T ANSWER". DO YOU AGREE?" WITH RESPONSE OPTIONS "YES" AND "NO".]

How much do you trust various citizen groups? For each, indicate the extent to which you trust the people in that group on a scale from 0 to 10.

[PROGRAMMER: RANDOMLY ROTATE THE ORDER OF QUESTIONS FOR EACH RESPONDENT]

[PROGRAMMER: ON MOBILE DEVICES, RANDOMLY ROTATE ORDER FOR EACH RESPONDENT, VERTICAL ORIENTATION AND SEPARATE INTO SCREENS WITH TWO OR THREE ITEMS PER SCREEN].

		I don't trust it at all									I have every confidence
**p14a_3**	PP voters	0	1	2	3	4	5	6	7	8	9	10
**p14b_3**	PSOE voters	0	1	2	3	4	5	6	7	8	9	10
**p14c_3**	Ciudadanos voters	0	1	2	3	4	5	6	7	8	9	10
**p14d_3**	Podemos and IU voters	0	1	2	3	4	5	6	7	8	9	10
**p14e_3**	ERC voters	0	1	2	3	4	5	6	7	8	9	10
**p14f_3**	Junts per Catalunya voters	0	1	2	3	4	5	6	7	8	9	10
**p14g_3**	EAJ-PNV voters	0	1	2	3	4	5	6	7	8	9	10
**p14h_3**	Vox voters	0	1	2	3	4	5	6	7	8	9	10

FOR QUESTIONS p14a_3 TO p14h_3 [PROGRAMMER: DON'T KNOW...888 (GENERATED AUTOMATICALLY IF RESPONDENTS MOVE ON TO THE NEXT QUESTION WITHOUT ANSWERING AND AFTER INSISTING)]

FOR QUESTIONS p14a_3 TO p14h_3 [PROGRAMMER: IF RESPONDENTS GO FORWARD WITHOUT ANSWERING, DISPLAY A MESSAGE THAT SAYS "IF YOU WILL GO FORWARD WITHOUT ANSWERING ONE OF THE QUESTIONS ON THIS SCREEN, YOUR ANSWER WILL BE RECORDED AS "DOESN'T KNOW / DOESN'T ANSWER" FOR THAT QUESTION. DO YOU AGREE?" WITH RESPONSE OPTIONS "YES" AND "NO".]

[IMPORTANT: ON MOBILE DEVICES ORIENTATION MUST BE HORIZONTAL]

[PROGRAMMER: ROTATE QUESTIONS RANDOMLY AND PRESENT THEM ON SEPARATE SCREENS]

[IMPORTANT: ON MOBILE DEVICES ORIENTATION MUST BE HORIZONTAL]

Could you please tell us on a scale from 1 to 7, where 1 is "I don't trust at all" and 7 is "I completely trust", how much you trust each of the following political institutions...

[PROGRAMMER: PUT ON NEW SCREEN]

trust1a_3 ...the Spanish Parliament

I don't trust it at all						I completely trust it
1	2	3	4	5	6	7

trust1b_3 ...the Spanish government

[PROGRAMMER: PUT ON NEW SCREEN]

I don't trust it at all						I completely trust it
1	2	3	4	5	6	7

trust1c_3 ...the Parliament of **[PROGRAMMER:** AUTONOMOUS COMMUNITY**]**

[PROGRAMMER: PUT ON NEW SCREEN]

I don't trust it at all						I completely trust it
1	2	3	4	5	6	7

trust1d_3 ...the government of **[PROGRAMMER:** AUTONOMOUS COMMUNITY**]**

[PROGRAMMER: PUT ON NEW SCREEN]

I don't trust it at all						I completely trust it
1	2	3	4	5	6	7

trust1e_3 ...politicians in Spain

[PROGRAMMER: PUT ON NEW SCREEN]

I don't trust them at all						I completely trust them
1	2	3	4	5	6	7

trust1f_3 ...political parties in Spain

[PROGRAMMER: PUT ON NEW SCREEN]

I don't trust them at all.						I completely trust them
1	2	3	4	5	6	7

trust1g_3 ...the Spanish police

[PROGRAMMER: PUT ON NEW SCREEN]

I don't trust it at all						I completely trust it
1	2	3	4	5	6	7

trust1h_3 ...the Spanish judicial system

[PROGRAMMER: PUT ON NEW SCREEN]

I don't trust it at all						I completely trust it
1	2	3	4	5	6	7

trust1i_3 ...the European Parliament

[PROGRAMMER: PUT ON NEW SCREEN]

I don't trust it at all						I completely trust it
1	2	3	4	5	6	7

trust1j_3 ...the government of the European Union (The European Commission)

[PROGRAMMER: PUT ON NEW SCREEN]

I don't trust it at all						I completely trust it
1	2	3	4	5	6	7

p12a_3 In general, would you say that you can trust most people, or that you can never be too careful in dealing with others? Please place yourself on the following scale from 0 to 10.

[PROGRAMMER: ON MOBILE DEVICES, VERTICAL ORIENTATION]

You can never be too careful										Most people can be trusted
0	1	2	3	4	5	6	7	8	9	10

p12b_3 And do you think that most people would try to take advantage of you if they could, or that they would be honest with you?

[PROGRAMMER: ON MOBILE DEVICES, VERTICAL ORIENTATION]

Most people would try to take advantage of me										Most people would be honest with me
0	1	2	3	4	5	6	7	8	9	10

p12c_3 Would you say that most of the time people try to help others or that they mainly look out for themselves?

[PROGRAMMER: ON MOBILE DEVICES, VERTICAL ORIENTATION]

Most of the time people look out for themselves										Most of the time people try to help others
0	1	2	3	4	5	6	7	8	9	10

I would now ask you to pay attention to this list of political leaders and tell me in each case whether you know them and how you would assess their political activity on the following scale:

[PROGRAMMER: ROTATE ORDER OF QUESTIONS AT RANDOM]

p40a_3 Pablo Casado

1 I know him

2 I don't know him **[PROGRAMMER: GO TO NEXT CANDIDATE AND THE NEXT QUESTION IS 999 MISSING]**

p41a_3

[PROGRAMMER: VERTICAL ORIENTATION ON MOBILE DEVICES]

Very badly										Very good
0	1	2	3	4	5	6	7	8	9	10

p40b_3 Pedro Sánchez

1 I know him

2 I don't know him **[PROGRAMMER: GO TO NEXT CANDIDATE AND THE NEXT QUESTION IS 999 MISSING]**

p41b_3

[PROGRAMMER: VERTICAL ORIENTATION ON MOBILE DEVICES]

Very badly										Very good
0	1	2	3	4	5	6	7	8	9	10

p40c_3 Albert Rivera

1 I know him
2 I don't know him [PROGRAMMER: GO TO NEXT CANDIDATE AND THE NEXT QUESTION IS 999 MISSING]

p41c_3

[PROGRAMMER: VERTICAL ORIENTATION ON MOBILE DEVICES]

Very badly										Very good
0	1	2	3	4	5	6	7	8	9	10

p40d_3 Pablo Iglesias

1 I know him
2 I don't know him [PROGRAMMER: GO TO NEXT CANDIDATE AND THE NEXT QUESTION IS 999 MISSING]

p41d_3

[PROGRAMMER: VERTICAL ORIENTATION ON MOBILE DEVICES]

Very badly										Very good
0	1	2	3	4	5	6	7	8	9	10

p40e_3 Alberto Garzón

1 I know him
2 I don't know him [PROGRAMMER: GO TO NEXT CANDIDATE AND THE NEXT QUESTION IS 999 MISSING]

p41e_3

[PROGRAMMER: VERTICAL ORIENTATION ON MOBILE DEVICES]

Very badly										Very good
0	1	2	3	4	5	6	7	8	9	10

p40f_3 Iñigo Urkullu [PROGRAMMER: ASK ONLY IN THE BASQUE COUNTRY]

1 I know him
2 I don't know him [PROGRAMMER: GO TO NEXT CANDIDATE AND THE NEXT QUESTION IS 999 MISSING]

p41f_3

[PROGRAMMER: VERTICAL ORIENTATION ON MOBILE DEVICES]

Very badly										Very good
0	1	2	3	4	5	6	7	8	9	10

p40g_3 Carles Puigdemont [PROGRAMMER: ASK ONLY IN CATALONIA]

1 I know him
2 I don't know him [PROGRAMMER: GO TO NEXT CANDIDATE AND THE NEXT QUESTION IS 999 MISSING]

p41g_3

[PROGRAMMER: VERTICAL ORIENTATION ON MOBILE DEVICES]

Very badly										Very good

0	1	2	3	4	5	6	7	8	9	10

p40h_3 Oriol Junqueras [PROGRAMMER: ASK ONLY IN CATALONIA]

1 I know him
2 I don't know him **[PROGRAMMER: GO TO NEXT CANDIDATE AND THE NEXT QUESTION IS 999 MISSING]**

p41h_3

[PROGRAMMER: VERTICAL ORIENTATION ON MOBILE DEVICES]

**Very badly**										**Very good**
0	1	2	3	4	5	6	7	8	9	10

HERE I PROPOSE TO PUT THIS LEADERSHIP BRAND BATTERY. AS THERE ARE SEVERAL QUESTIONS FOR EACH OF THE FOUR LEADERS, I PROPOSE THAT FOR EACH WAVE (THIS THIRD AND FOURTH) ONLY TWO LEADER BATTERIES APPEAR AND THAT THEY ARE SELECTED IN A RANDOM WAY. THIS WAY, AT THE END OF THE FOUR WAVES WE WILL HAVE THIS INFORMATION FOR THE FOUR LEADERS.

PROGRAMMER: IF IN THE PREVIOUS BATTERY IT SAYS IN p40a_3, p40b_3, p40c_3, p40d_3 NOT TO KNOW THE LEADER, DO NOT MAKE THE NEXT BATTERY CORRESPONDING TO THAT LEADER. IN THAT CASE AUTOMATICALLY CODE ALL THESE QUESTIONS AS 888, I DON'T KNOW IT.

[BATTERY CASADO]

We'd now like to know what you think of Pablo Casado. Read the list of adjectives below and tell us how much you agree or disagree that each characteristic describes him.

**PROGRAMMER: RANDOMLY ROTATE THE RESPONSE CATEGORIES**		Strongly agree	Somewhat agree	Neither agree nor disagree	Somewhat disagree	Strongly disagree
**p54a_3**	Decisive	1	2	3	4	5
**p54b_3**	Intelligent	1	2	3	4	5
**p54c_3**	Charismatic	1	2	3	4	5
**p54d_3**	Incompetent	1	2	3	4	5
**p54e_3**	Caring	1	2	3	4	5
**p54f_3**	Reliable	1	2	3	4	5
**p54g_3**	Dishonest	1	2	3	4	5
**p54h_3**	Arrogant	1	2	3	4	5

p55_3 To what extent do you consider that the personal characteristics above that you believe define Pablo Casado define you as well?

1 A lot
2 A fair amount
3 Somewhat
4 A little
5 Not at all

p56_3 To what extent do you admire the qualities that define Pablo Casado?

1 A lot
2 A fair amount
3 Somewhat

4 A little
5 Not at all

To what extent do you agree or disagree with the following statements?

PROGRAMMER: RANDOMLY ROTATE THE RESPONSE CATEGORIES		Strongly agree	Somewhat agree	Neither agree nor disagree	Somewhat disagree	Strongly disagree
**p57a_3**	I identify with those who express support for Pablo Casado	1	2	3	4	5
**p57b_3**	I identify with Pablo Casado	1	2	3	4	5
**p57c_3**	Pablo Casado shares my beliefs or convictions	1	2	3	4	5
**p57d_3**	I have no problem telling people that I voted for Pablo Casado	1	2	3	4	5
**p57e_3**	Pablo Casado represents the ideals of his party	1	2	3	4	5

[BATTERY SÁNCHEZ]

We'd like to know what you think of Pedro Sánchez. Read the list of adjectives below and tell us how much you agree or disagree that each characteristic describes him.

PROGRAMMER: RANDOMLY ROTATE THE RESPONSE CATEGORIES		Strongly agree	Somewhat agree	Neither agree nor disagree	Somewhat disagree	Strongly disagree
**p58a_3**	Decisive	1	2	3	4	5
**p58b_3**	Intelligent	1	2	3	4	5
**p58c_3**	Charismatic	1	2	3	4	5
**p58d_3**	Incompetent	1	2	3	4	5
**p58e_3**	Caring	1	2	3	4	5
**p58f_3**	Reliable	1	2	3	4	5
**p58g_3**	Dishonest	1	2	3	4	5
**p58h_3**	Arrogant	1	2	3	4	5

p59_3 To what extent do you consider that the personal characteristics above that you think define Pedro Sánchez define you as well?

1 A lot
2 A fair amount
3 Somewhat
4 A little
5 Not at all

p60_3 To what extent do you admire the qualities that define Pedro Sánchez?

1 A lot
2 A fair amount
3 Somewhat
4 A little
5 Not at all

To what extent do you agree or disagree with the following statements?

PROGRAMMER: RANDOMLY ROTATE THE RESPONSE CATEGORIES		Strongly agree	Somewhat agree	Neither agree nor disagree	Somewhat disagree	Strongly disagree
**p61a_3**	I identify with those who express support for Pedro Sánchez	1	2	3	4	5
**p61b_3**	I identify with Pedro Sánchez	1	2	3	4	5
**p61c_3**	Pedro Sánchez shares my beliefs or convictions	1	2	3	4	5
**p61d_3**	I have no problem telling people that I voted for Pedro Sánchez	1	2	3	4	5
**p61e_3**	Pedro Sánchez represents the ideals of his party	1	2	3	4	5

[BATTERY RIVERA]

We'd like to know what you think of Albert Rivera. Read the list of adjectives below and tell us how much you agree or disagree that each characteristic describes him.

PROGRAMMER: RANDOMLY ROTATE THE RESPONSE CATEGORIES		Strongly agree	Somewhat agree	Neither agree nor disagree	Somewhat disagree	Strongly disagree
**p62a_3**	Decisive	1	2	3	4	5
**p62b_3**	Intelligent	1	2	3	4	5
**p62c_3**	Charismatic	1	2	3	4	5
**p62d_3**	Incompetent	1	2	3	4	5
**p62e_3**	Caring	1	2	3	4	5
**p62f_3**	Reliable	1	2	3	4	5
**p62g_3**	Dishonest	1	2	3	4	5
**p62h_3**	Arrogant	1	2	3	4	5

p63_3 To what extent do you consider that the personal characteristics above that you think define Albert Rivera define you as well?

1 A lot
2 A fair amount
3 Somewhat
4 A little
5 Not at all

p64_3 To what extent do you admire the qualities that define Albert Rivera?

1 A lot
2 A fair amount
3 Somewhat
4 A little
5 Not at all

To what extent do you agree or disagree with the following statements?

PROGRAMMER: RANDOMLY ROTATE THE RESPONSE CATEGORIES		Strongly agree	Somewhat agree	Neither agree nor disagree	Somewhat disagree	Strongly disagree
**p65a_3**	I identify with those who express support for Albert	1	2	3	4	5

	Rivera					
**p65b_3**	I identify with Albert Rivera	1	2	3	4	5
**p65c_3**	Albert Rivera shares my beliefs or convictions	1	2	3	4	5
**p65d_3**	I have no problem telling people that I voted for Albert Rivera	1	2	3	4	5
**p65e_3**	Albert Rivera represents the ideals of his party	1	2	3	4	5

[BATTERY IGLESIAS]

We'd like to know what you think of Pablo Iglesias. Read the list of adjectives below and tell us how much you agree or disagree that each characteristic describes him.

**PROGRAMMER: RANDOMLY ROTATE THE RESPONSE CATEGORIES**		Strongly agree	Somewhat agree	Neither agree nor disagree	Somewhat disagree	Strongly disagree
**p66a_3**	Decisive	1	2	3	4	5
**p66b_3**	Intelligent	1	2	3	4	5
**p66c_3**	Charismatic	1	2	3	4	5
**p66d_3**	Incompetent	1	2	3	4	5
**p66e_3**	Caring	1	2	3	4	5
**p66f_3**	Reliable	1	2	3	4	5
**p66g_3**	Dishonest	1	2	3	4	5
**p66h_3**	Arrogant	1	2	3	4	5

p67_3 To what extent do you consider that the personal characteristics above that you believe define Pablo Iglesias define you as well?

1 A lot
2 A fair amount
3 Somewhat
4 A little
5 Not at all

p68_3 To what extent do you admire the qualities that define Pablo Iglesias?

1 A lot
2 A fair amount
3 Somewhat
4 A little
5 Not at all

To what extent do you agree or disagree with the following statements?

**PROGRAMMER: RANDOMLY ROTATE THE RESPONSE CATEGORIES**		Strongly agree	Somewhat agree	Neither agree nor disagree	Somewhat disagree	Strongly disagree
**p69a_3**	I identify with those who express support for Pablo Iglesias	1	2	3	4	5
**p69b_3**	I identify with Pablo Iglesias	1	2	3	4	5
**p69c_3**	Pablo Iglesias shares my beliefs or convictions	1	2	3	4	5

**p69d_3**	I have no problem telling people that I voted for Pablo Iglesias	1	2	3	4	5
**p69e_3**	Pablo Iglesias represents the ideals of his party	1	2	3	4	5

FINAL PART LEADERSHIP BRAND

[PROGRAMMER: IF RESPONDENT ADVANCES WITHOUT ANSWERING, PRESENT A MESSAGE THAT SAYS "If you advance without answering this question, your answer will be recorded as "Don't know / Don't answer", do you agree? With response options "Yes" and "No".

Now, indicate through what means and how often you are keep informed about current issues. Keep in mind the importance of reading the questions carefully and choosing the answer that best fits your thoughts and opinions. The results and quality of this international research depend on your effort and attention to your responses.

Could you please say how often you keep yourself informed about current political issues, news or opinions through…

[PROGRAMMER: RANDOMLY ROTATE THE ORDER OF ITEMS FOR EACH RESPONDENT]

[PROGRAMMER: ON MOBILE DEVICES, VERTICAL ORIENTATION]

		**Never**	**Less than once a month**	**Once a month**	**Several times a month**	**Once a week**	**Several times a week**	**Every day**	**Several times a day**
**p17a_3**	Newspapers	0	1	2	3	4	5	7	8
**p17b_3**	Radio	0	1	2	3	4	5	7	8
**p17c_3**	Magazines	0	1	2	3	4	5	7	8
**p17d_3**	Television	0	1	2	3	4	5	7	8
**p17e_3**	Social networks	0	1	2	3	4	5	7	8

p26a_3 How often do you talk about politics or political issues with relatives, friends, colleagues or acquaintances?

1 Less than once a month
2 Once a month
3 Several times a month
4 Once a week
5 Several times a week
6 Every day
0 Never **[GO TO p19a_3]**

[PROGRAMMER: IN THIS CASE AUTOMATICALLY CODE 999 FOR QUESTIONS p27a_3, p28a_3 and p29a_3]

p27a_3 How often do you agree with the views of the people with whom you talk about politics?

3 Always
2 Many times
1 Sometimes

0 Never
888 Doesn't Know/No Answer

p28a_3 Also, how often do you disagree with the views of the people with whom you talk about politics?

3 Always
2 Many times
1 Sometimes
0 Never
888 Doesn't Know/No Answer

p29a_3 Do you think the people you talk to about politics...?

3 They support the same party as you
2 They divide their support among different parties
1 They support a different party than yours
0 They do not support any party
888 Doesn't Know/No Answer

Do you have an account on one of the following social networks?

		Yes	No
**p19b_3**	Facebook	1	0
**p19c_3**	Google +	1	0
**p19d_3**	LinkedIn	1	0
**p19e_3**	Instagram	1	0
**p19f_3**	Flickr	1	0
**p19g_3**	YouTube	1	0
**p19i_3**	WhatsApp	1	0
**p19h_3**	Other ________	1	0

[PROGRAMMER: IF RESPONDENT SAYS "NO" ON p19a_3, GO TO p26b_3]

p26b_3 How often do you discuss politics or current political issues on social networks, Facebook, Twitter or any other blog?

[PROGRAMMER: ASK ONLY THOSE WHO ANSWERED 1/YES ON ONE OF THE p19a_3-p19h_3; PUT 999 FOR ALL OTHERS]

1 Less than once a month
2 Once a month
3 Several times a month 4
Once a week
5 Several times a week
6 Every day
0 Never **[GO TO p30_3]**
[PROGRAMMER: IN THIS CASE AUTOMATICALLY CODE 999 FOR QUESTIONS p27b_3 to p29b_3]

p27b_3 How often do you agree with the views of the people with whom you talk about politics in these forums?

3 Always
2 Many times
1 Sometimes
0 Never
888 don't know don't answer

p28b_3 Also, how often do you disagree with the views of the people with whom you talk about politics in

these forums?

3 Always
2 Many times
1 Sometimes
0 Never
888 don't know don't answer

p29b_3 Do you think that the people you talk to about politics in these forums...

3 They support the same party as you
2 They divide their support among different parties
1 They support a different party than yours
0 They don't support any party
888 don't know don't answer

[PROGRAMMER: THIS QUESTION IS ONLY FOR THOSE WHO ANSWERED YES (1) to some of the social networks in P19a, P19b, P19d, P19e and P19f.]

How often have you seen political information on Twitter or other social networks coming from... ?

	Every day or almost every day	Several days a week	Only on weekends	From time to time	Never or hardly ever	I don't follow these profiles
**p70a_3 Family and friends**	1	2	3	4	5	999
**p70b_3 Political parties and candidates**	1	2	3	4	5	999
**p70c_3 Media**	1	2	3	4	5	999
**p70d_3 Journalists**	1	2	3	4	5	999
**p70e_3 Celebrities**	1	2	3	4	5	999

[PROGRAMMER: THIS QUESTION IS ONLY FOR THOSE WHO HAVE ANSWERED YES (1) to some of the social networks in P19a, P19b, P19d, P19e and P19f.]

How much do you trust the information shared on social networks from...?

	Completely	Somewhat	Neither a little nor a lot	A little	Not at all
**p71a_3** Family and friends	1	2	3	4	5

**p71b_3** Political parties and candidates	1	2	3	4	5
**p71c_3** Media	1	2	3	4	5
**p71d_3** Journalists	1	2	3	4	5
**p71e_3** Celebrities	1	2	3	4	5

Now we'll talk about aspects of your political preferences. Remember again the importance of reading the questions carefully and choosing the answer that best fits your thoughts and opinions. The results and quality of this international research depend on your efforts and attention to detail. We remind you that your answers will remain anonymous and will only be treated, along with those of other respondents, in a statistical manner.

p35_3 Do you consider yourself close to any political party?

1 Yes **[GO TO p35a_3]**
0 No **[GO TO s8_3]**

p35a_3 Which one?

[PROGRAMMER: ASK IF 1 ON p35_3. CODE 999 FOR THE OTHERS]

1 PP (Popular Party)
2 PSOE (Spanish Socialist Workers' Party)
3 Podemos and other affiliated municipal lists (En Comú Podem, Ahora Madrid)
4 IU (United Left)
5 Ciudadanos (C's - Ciutadans)
6 ERC (Esquerra Republicana de Catalunya)
7 7 JxCat (Junts per Catalunya)
8 EAJ - PNV (Euzko Alderdi Jeltzalea - Basque Nationalist Party)
9 EH - Bildu (Euskal Herria - Bildu)
11 CC (Canary Islands Coalition)
13 VOX
12 Others___________

p35b_3 And how close do you feel to this party?

[PROGRAMMER: ASK IF 1 ON p35_3. code 999 TO THE OTHERS]

3 Very close
2 Somewhat close
1 Not very close
0 Not at all close

p35c_3 Is it important for you to be from the [PROGRAMMER: PUT PARTY NAME CHOSEN FROM p35a_3]?

[PROGRAMMER: ASK IF 1 ON p35_3. CODE 999 FOR OTHERS]

3 Extremely important
2 Very important
1 Not very important
0 Not at all important

p35d_3 How well does the word supporters of [PROGRAMMER: PUT PARTY NAME FROM p35a_3] describe you?

[PROGRAMMER: ASK IF 1 ON p35_3. CODE 999 FOR THE OTHERS]

3 Extremely well
2 Very good
1 Not very well
0 Not at all

p35e_3 When you talk about [PROGRAMMER: INSERT PARTY NAME CHOSEN IN p35a_2], how often do you use the word "we"?

3 Always
2 Most of the time
1 Sometimes
0 Never

[PROGRAMMER: ASK IF 1 ON p35_3. CODE 999 FOR OTHERS]

p35f_3 To what extent do you consider yourself "one" of the [PROGRAMMER: PUT PARTY NAME FROM p35a_3]?

[PROGRAMMER: ASK IF 1 ON p35_3. CODE 999 FOR OTHERS]

3 A lot
2 Some
1 Little
0 Not at all

I would now like to ask you what you think of the political parties that have the most electoral support. Please rate each of them on this scale from 0 to 10 where 0 means that you don't like the party at all, and 10 means that you like it very much.

		I don't like it at all										I like it very much	I don't know
**p72a_3**	PP (People's Party)	0	1	2	3	4	5	6	7	8	9	10	888
**p72b_3**	PSOE (Spanish Socialist Workers' Party)	0	1	2	3	4	5	6	7	8	9	10	888
**p72c_3**	Unidas Podemos (En Comú Podem)	0	1	2	3	4	5	6	7	8	9	10	888
**p72e_3**	Ciudadanos (C's - Ciutadans)	0	1	2	3	4	5	6	7	8	9	10	888
**p72f_3**	ERC (Esquerra Republicana de Catalunya)	0	1	2	3	4	5	6	7	8	9	10	888
**p72g_3**	JxCat (Junts per Catalunya)	0	1	2	3	4	5	6	7	8	9	10	888
**p72h_3**	EAJ-PNV (Euzko Alderdi Jeltzalea, Basque Nationalist Party)	0	1	2	3	4	5	6	7	8	9	10	888
**p72i_3**	EH-Bildu (Euskal Herria- Bildu)	0	1	2	3	4	5	6	7	8	9	10	888
**p72l_3**	Vox	0	1	2	3	4	5	6	7	8	9	10	888

**FROM HERE, ONLY TO THE RESPECTIVE AUTONOMOUS COMMUNITIES**													
**p72p_3**	FAC (Citizens' Forum) **ASTURIAS**	0	1	2	3	4	5	6	7	8	9	10	888
**p72n_3**	CC (Canarian Coalition) **CANARY ISLANDS**	0	1	2	3	4	5	6	7	8	9	10	888
**p72m_3**	Compromís **VALENCIAN C.**	0	1	2	3	4	5	6	7	8	9	10	888
**p72o_3**	En Marea **GALICIA**	0	1	2	3	4	5	6	7	8	9	10	888

p73_3 Now, we would like you to tell us what is the probability that you will vote in the next general election on April 28th, using a scale from 0 to 10, where 0 means "you will definitely not vote" and 10 means "you will definitely vote".

**You're definitely not going to vote**										**You're definitely going to vote**
00	01	02	03	04	05	06	07	08	09	10

There are many political parties in Spain that would like to count on your vote. What is the probability that you will ever vote for one of the following political parties? Please specify your opinion on a scale from 0 to 10, where 0 means "Not at all likely" and 10 means "Very likely".

		**Not at all likely**										**Very likely**	**I don't know**
**p74a_3**	PP (People's Party)	00	01	02	03	04	05	06	07	08	09	10	888
**p74b_3**	PSOE (Spanish Socialist Workers' Party)	00	01	02	03	04	05	06	07	08	09	10	888
**p74c_3**	Unidas Podemos (En Comú Podem)	00	01	02	03	04	05	06	07	08	09	10	888
**p74e_3**	Ciudadanos (C's - Ciutadans)	00	01	02	03	04	05	06	07	08	09	10	888
**p74f_3**	ERC (Esquerra Republicana de Catalunya)	00	01	02	03	04	05	06	07	08	09	10	888
**p74g_3**	JxCat (Junts per Catalunya)	00	01	02	03	04	05	06	07	08	09	10	888
**p74h_3**	EAJ-PNV (Euzko Alderdi Jeltzalea, Basque Nationalist Party)	00	01	02	03	04	05	06	07	08	09	10	888
**p74i_3**	EH-Bildu (Euskal Herria- Bildu)	00	01	02	03	04	05	06	07	08	09	10	888
**p74l_3**	Vox	00	01	02	03	04	05	06	07	08	09	10	888
	**FROM HERE, ONLY TO THE RESPECTIVE AUTONOMOUS COMMUNITIES**												
**p74p_3**	FAC (Citizens' Forum) **ASTURIAS**	00	01	02	03	04	05	06	07	08	09	10	888
**p74n_3**	CC (Canarian Coalition) **CANARY ISLANDS**	00	01	02	03	04	05	06	07	08	09	10	888
**p74m_3**	Compromís **VALENCIAN C.**	00	01	02	03	04	05	06	07	08	09	10	888
**p74o_3**	En Marea **GALICIA**	00	01	02	03	04	05	06	07	08	09	10	888

**p74q_3**	Partido Regionalista de Cantabria (PRC) CANTABRIA	00	01	02	03	04	05	06	07	08	09	10	888

p75_3 Which party do you plan to vote for in the next general election on April 28?

PP (1)
PSOE (2)
Unidas Podemos (3)
C's (5)
Vox (13)
Junts per Catalunya (7)
PNV-EAJ (8)
EH-Bildu (9)
ERC (6)
CC (11)
En Comú Podem (4)
Compromís (14)
En Marea (15)
Partido Regionalista de Cantabria (16) Other (12)
Blank vote (20)
I will not vote (21)
I do not have the right to vote (22)
I don't know (23)
I don't want to say it (24)

[PROGRAMMER: IF RESPONDENT DOESN'T ANSWER, CODE 888]

s8_3 And which best describes your employment situation in the last seven days? Please choose only one of the following options.

1 Employed, but on temporary leave (includes temporary maternity/paternity leave, accident, illness or holidays).
2. Employed, self-employed, or in a family business
2 Studying, even if you have been on holiday (includes company paid training)
3 Unemployed and actively seeking work
4 Unemployed, wanting to find a job but not actively looking for one
5 With chronic illness or permanent disability
6 Retired
7 Homemaker, stay-at-home parent, or caregiver

s9_3 Which of the statements below best describes how you feel about your current household income?

1 With our current income we live comfortably
2 With our current income we get by
3 With our current income we have difficulties
4 With our current income we have many difficulties

s10_3 Have you been fired from your primary employment at any time in the past year?

1 Yes
2 No

Currently, to what extent do you feel concerned about...

[PROGRAMMER: ROTATE ORDER RANDOMLY FOR EACH RESPONDENT]

		Not at all concerned	Not too concerned	Quite concerned	Very concerned
**s11a_3**	Paying your household bills	0	1	2	3
**s11b_3**	Having to reduce your standard of living	0	1	2	3
**s11c_3**	Having a job	0	1	2	3
**s11d_3**	Paying off loans from the bank or paying mortgage bills	0	1	2	3
