# Supplementary material for: Political and affective polarisation in a democracy in crisis: The E-Dem panel survey dataset (Spain, 2018–2019)
Source: Data Brief. 2020 Jul 23;32:106059. doi: 10.1016/j.dib.2020.106059 (PMC7451797; doi:10.1016/j.dib.2020.106059)
Supplement: Supplementary file 5 [file mmc5.pdf]

Online political participation and deliberation in a democracy in crisis

Wave IV Questionnaire

May 2019

Project: *Online Political Participation and Deliberation in a Democracy in Crisis: A New Methodological Approach (E-Dem) (2017-2020)*

PI: Mariano Torcal. Ministry of Economy and Competitiveness, State Programme for the Promotion of Scientific and Technical Research of Excellence, 2017. Ref: CSO2016-79772-P.

Universitat Pompeu Fabra Barcelona

RECSM Research and Expertise Centre for Survey Methodology

[GENERAL INSTRUCTIONS]

1. Do not force the panellist to answer all the questions

2. Allow them not to answer the questions by moving on to the next one, although a warning message must be issued where they have to confirm their choice every 4 questions

3. In some important questions the message applies

4. Some knowledge questions include the category don't know or prefer not to answer

5. In the data file, the name of variables must appear exactly as in the questionnaire.

6. It is also important to start the questionnaire with a short introduction:

This survey aims to provide the data necessary to analyse aggregate opinions on current issues such as immigration or the pension system, as well as on political trends. It is a study led by researchers from the Pompeu Fabra University, within a European research project on online political participation and deliberation. All the information you and other respondents provide will be shared with that University anonymously and used only for the research purposes mentioned above. If you wish to exercise your right to data protection, you may write to the Department of Political Science at the Pompeu Fabra University, located at c/ Ramon Trias Fargas, 25 -27, 08005 Barcelona (Edificio Jaume I - Campus Ciutadella) or to the following e-mail address: departament.cpis@upf.edu.

Below, we ask you to confirm if you would like to participate in this interesting survey:

- ○ Yes, I want to participate
- ○ No, I'd rather not participate

SECOND SCREEN:

You may not remember, a few weeks ago you participated in a survey designed by a group of national and international researchers, as part of a project interested in studying the opinion of people like you on issues related to our political system and our society, led by a professor at the Pompeu Fabra University. To this end, we would like to count again on your participation in another survey that has an approximate duration of 20-25 minutes. In this way, you will be part of the same 2,500 people from all over Spain who also participated in the previous study.

Your answers are very important and will help to know the opinions of the Spanish people about the political current situation and the functioning of the democracy in the country. The problems and challenges of our political and social systems require a good study of citizens' opinions and therefore your attentive and dedicated participation is essential. In any case, as with the previous survey, your answers will remain in total anonymity and will only be subject to statistical analysis together with the other hundreds of participants.

We'd like to start by asking you this:

p19a_4 Do you have a Twitter account?

1. Yes. **[PROGRAMMER: GO TO QUESTION pi17e_3]**
2. No. **[PROGRAMMER: SKIP TO QUESTION p1_4 OF THE GENERAL QUESTIONNAIRE]**

p19a1_4 Are you following the election campaign at all on Twitter?

---

1. Yes **[PROGRAMMER: GO TO THE EXPERIMENT]**
2. No. **[PROGRAMMER: SKIP TO QUESTION p1_4 OF THE GENERAL QUESTIONNAIRE]**

EXPERIMENT:

PROGRAMMER: ONLY FOR THOSE WHO CLAIM TO HAVE A TWITTER ACCOUNT AND FOLLOW THE INFORMATION ABOUT THE CAMPAIGN ON THIS CHANNEL. FOR THE OTHER RESPONDENTS, ALL QUESTIONS UNTIL p1_4 ARE CODED WITH 999. THOSE NOT SELECTED FOR THE EXPERIMENT HAVE FOR ALL THESE QUESTIONS A 999 CODE

We would like to provide you with some information about some of the discussions that are taking place among voters related to the upcoming elections. To do so, we invite you to explore one or more of the following accounts.

With the link below you can find some Twitter accounts where fundamental aspects for this election campaign are being discussed. We invite you to choose one of them, read what is being discussed; you may even participate in it If you wish - we leave that to your choice. We will give you approximately five days to do so. After that period of time, you will receive an invitation to return to the survey.

PROGRAMMER: RANDOMLY SELECT OPTION A OR OPTION B

OPTION A

PARTIDO SOCIALISTA OBRERO ESPAÑOL (PSOE)
C: Josep Borrell Fontelles
https://twitter.com/josepborrellf?lang=es

UNIDAS PODEMOS CAMBIAR EUROPA (Podemos-IU)
C: Maria Eugenia Rodriguez Palop
https://twitter.com/meugeniarpalop?lang=es

CIUDADANOS-PARTIDO DE LA CIUDADANÍA (Cs)
C: Luis Garicano Gabilondo.
https://twitter.com/lugaricano?lang=es

VOX (VOX)
C: Jorge Buxadé Villalba
https://twitter.com/jorgebuxade?lang=es

PARTIDO POPULAR (PP)
C:Dolors Montserrat Montserrat.
https://twitter.com/dolorsmm?lang=es

COMPROMÍS PER EUROPA/COMPROMISO POR EUROPA (CPE)
C: Jordi Sebastià Talavera (COMMITMENT).
https://twitter.com/sebastia_jordi?lang=es

[PROGRAMMER: ONLY CATALONIA, BASQUE COUNTRY, ASTURIAS, ARAGON AND CANARY ISLANDS]
AHORA REPÚBLICAS
C: Oriol Junqueras
https://twitter.com/junqueras

[PROGRAMMER: CATALONIA ONLY]
JUNTA PER CATALUNYA C: Carles Puigdemont.
https://twitter.com/krls?lang=es

[PROGRAMMER: ONLY BASQUE COUNTRY, BALEARIC, VALENCIA AND GALICIA]
COALICIÓN POR UNA EUROPA SOLIDARIA
PNV, Coalición Canaria, Compromiso por Galicia, Agrupación Atarrabia, Proposta per les Illes Balears and Democrates Valencians will go together to the European elections under this name
C: Izaskun Bilbao (EAJ-PNV)
https://twitter.com/izaskunbilbaob?lang=es

OPTION B:

European Parliament
https://twitter.com/pe_espana?lang=es

European Parliament in Spain
https://twitter.com/europarl_es?lang=es

European Commission in Spain
https://twitter.com/uemadrid

euronews in Spanish
https://twitter.com/euronewses?lang=es

esm0a_4. Would you like to participate in this experiment?

We remind you that your answers are very important and will help to know the opinions of the Spanish people about the current political situation and the functioning of the democracy in the country. The problems and challenges of our political and social systems require a high-quality study of citizens' opinion and, therefore, your attentive and dedicated participation is essential. In any case, as with the previous survey, your answers will remain in total anonymity and they will only be subject to statistical analysis together with hundreds of other participants.

1. **Yes I want to participate → [PROGRAMMER: PROVIDE MESSAGE: "you will be contacted after 5 days"]**
2. **I do not want to participate →[PROGRAMMER: FOLLOW p1_4 THE REST OF QUESTIONS esm_ ARE CODED 999]**

[PROGRAMMER: AFTER FIVE DAYS, THOSE SELECTED FOR THE EXPERIMENT (THAT IS, THOSE WHO ANSWERED "yes" to esm0a_4, p19a_4 and p19a1_4) ARE RECONTACTED FIVE DAYS LATER, AND THE QUESTIONNAIRE STARTS HERE]

[EXPERIMENT FILTER QUESTIONS TO BE CARRIED OUT AFTER FIVE DAYS]

A few days ago we asked you to follow some of the politicians' accounts on the social network Twitter.
esmP1_4. To begin with, could you tell me if you finally did that?

1. Yes **[PROGRAMMER: CONTINUE WITH esmP7_4]**
2. No **[PROGRAMMER: GO TO GENERAL QUESTIONNAIRE p1_4 THE REST OF QUESTIONS esm_ CODED 999]**

esmP7_4 Could you indicate which one of the accounts you followed in these five days? If you have followed more than one, indicate the one you have followed the most.

[FOR THOSE WHO WERE SELECTED FOR OPTION A]

PARTIDO SOCIALISTA OBRERO ESPAÑOL (PSOE)…………………………1
C: Josep Borrell Fontelles
T: @JosepBorrellF

UNIDAS PODEMOS CAMBIAR EUROPA (Podemos-IU)…………………2
C: Maria Eugenia Rodriguez Palop
T: @MEugeniaRPalop

CIUDADANOS-PARTIDO DE LA CIUDADANÍA (Cs)…………………………3
C: Luis Garicano Gabilondo.
T: @lugaricano

VOX (VOX) ………… 4
C: Jorge Buxadé Villalba
T: @Jorgebuxade

PARTIDO POPULAR (PP)……………………………………………………………5
C:Dolors Montserrat Montserrat.
T: @DolorsMM

COMPROMÍS PER EUROPA/COMPROMISO POR EUROPA (CPE)…………6
C: Jordi Sebastià Talavera (COMMITMENT).
T: @Sebastia_Jordi

[PROGRAMMER: ONLY CATALONIA, BASQUE COUNTRY, ASTURIAS, ARAGON AND CANARY ISLANDS]
AHORA REPÚBLICAS……………………………………………………………………7
C: Oriol Junqueras
T: @junqueras

[PROGRAMMER: CATALONIA ONLY]
JUNTS PER CATALUNYA………………………………………………………………8
C: Carles Puigdemont.
T: @KRLS

[PROGRAMMER: ONLY BASQUE COUNTRY, BALEARIC, VALENCIA AND GALICIA]
COALICIÓN POR UNA EUROPA SOLIDARIA……………………………… 9
C: Izaskun Bilbao (EAJ-PNV)
T: @IzaskunBilbaoB

[FOR THOSE WHO WERE SELECTED FOR OPTION B]

European Parliament
@PE_English…………………………………………………………………… ………10

European Parliament in Spain
@Europarl_EN…………………………………………………………………… …….11

European Commission
@UEmadrid…………………………………………………………………….. ...…..12

euronews english
@euronewse………………………………………………………………………… 13

esmP8_4 Did you follow this account before we asked you to do so or did you begin to follow it only for the survey?

1. I followed it before

2. I've followed it only now

[PROGRAMMER: ROTATE THESE CATEGORIES]

esmP9_4 Could you indicate which of the following topics you think have been the most discussed in that account?

1. Issues related to the European Union
2. Issues related to Catalonia and the process of independence
3. Issues related to Spanish unity
4. Issues related to the economic situation in Spain
5. Issues related to the social situation in Spain
6. Issues related to the territorial model in Spain
7. Other current issues

esmP10_4 In general, to what extent would you say you agree with the most frequent positions in that account?

1. Strongly agree
2. Somewhat agree
3. Neither agree nor disagree
4. Somewhat disagree
5. Strongly disagree

esmP11_4 In general, how did you find the tone of the discussions that took place on that account?

1. Interesting
2. Very intolerant
3. Boring
4. Disrespectful
5. Informative
6. None of the above

888. I don't know

[PROGRAMMER: ROTATE THE FIRST FIVE OPTIONS]

esmP12_4 How much do you trust the information that you saw through that Twitter account?

1. Very reliable
2. Somewhat reliable
3. Somewhat unreliable
4. Not at all reliable

[PROGRAMMER: BEGINNING OF QUESTIONNAIRE FOR EVERYONE. THOSE NOT SELECTED FOR THE EXPERIMENT START HERE AFTER esmP12_4. AND HAVE 999 RECORDED FOR ALL QUESTIONS WITH PREFIX esm_]

p1_4 To begin with, to what extent are you interested in politics? A lot, a fair amount, a little or not at all?

1 A lot
2 A fair amount
3 A little
4 Not at all

p2_4 To what extent are you satisfied with the general economic situation in Spain? Indicate your answer on a scale from 0 to 10 where 0 is "Completely dissatisfied" and 10 is "Completely satisfied".

[PROGRAMMER: VERTICAL ORIENTATION ON MOBILE DEVICES]

Completely dissatisfied									Completely satisfied
0	1	2	3	4	5	6	7	8	9	10

p47_4 To what extent are you satisfied with the general political situation in Spain? Please indicate your answer on a scale from 0 to 10 where 0 is "Completely dissatisfied" and 10 is "Completely satisfied"

[PROGRAMMER: VERTICAL ORIENTATION ON MOBILE DEVICES]

Completely dissatisfied										Completely satisfied
0	1	2	3	4	5	6	7	8	9	10

p3_4 In your opinion, what is the main problem that currently exists in Spain? Please choose one of the following options:

[PROGRAMMER: RANDOMLY ROTATE THE ORDER OF THE TOPICS FOR EACH RESPONDENT]

1 Unemployment
2 Drugs
3 The healthcare system
4 Housing
5 Education
6 Domestic ETA terrorism
7 International terrorism (Islamic State/ISIS)
8 Corruption
9 Immigration
10 The Euro
11 Violence against women
12 Political instability
13 The refugee crisis
14 Environmental problems
15 Pensions
16 Citizen insecurity
17 Taxes
18 Parties and politicians in general
20 The situation in Catalonia
21 The economic situation
19 Others_______
888 I don't know

In your opinion, how would you rate the situation in Spain with respect to the following issues? Please indicate your answer on a scale from 0 to 10 where 0 is "Very bad" and 10 is "Very good"

[PROGRAMMER: RANDOMLY ROTATE THE ORDER OF THE TOPICS FOR EACH RESPONDENT, SEPARATED INTO SCREENS WITH TWO ITEMS PER SCREEN FOR A TOTAL OF 3 SCREENS]

[PROGRAMMER: VERTICAL ORIENTATION ON MOBILE DEVICES]

p4a_4 Unemployment

Very bad										Very good
0	1	2	3	4	5	6	7	8	9	10

[PROGRAMMER: DON'T KNOW...888 (GENERATED AUTOMATICALLY IF RESPONDENTS MOVE ON TO THE NEXT QUESTION WITHOUT ANSWERING AND AFTER INSISTING)]

p4b_4 Education

Very bad										Very good
0	1	2	3	4	5	6	7	8	9	10

[PROGRAMMER: DON'T KNOW...888 (GENERATED AUTOMATICALLY IF RESPONDENTS MOVE ON TO THE NEXT QUESTION WITHOUT ANSWERING AND AFTER INSISTING)]

p4c_4 Health

Very bad										Very good
0	1	2	3	4	5	6	7	8	9	10

[PROGRAMMER: DON'T KNOW...888 (GENERATED AUTOMATICALLY IF RESPONDENTS MOVE ON TO THE NEXT QUESTION WITHOUT ANSWERING AND AFTER INSISTING)]

p4d_4 Immigration

Very bad										Very good
0	1	2	3	4	5	6	7	8	9	10

[PROGRAMMER: DON'T KNOW...888 (GENERATED AUTOMATICALLY IF RESPONDENTS MOVE ON TO THE NEXT QUESTION WITHOUT ANSWERING AND AFTER INSISTING)]

p4e_4 The pension system

Very bad										Very good
0	1	2	3	4	5	6	7	8	9	10

[PROGRAMMER: DON'T KNOW...888 (GENERATED AUTOMATICALLY IF RESPONDENTS MOVE ON TO THE NEXT QUESTION WITHOUT ANSWERING AND AFTER INSISTING)]

p4f_4 Corruption

Very bad										Very good
0	1	2	3	4	5	6	7	8	9	10

[PROGRAMMER: DON'T KNOW...888 (GENERATED AUTOMATICALLY IF RESPONDENTS MOVE ON TO THE NEXT QUESTION WITHOUT ANSWERING AND AFTER INSISTING)]

p4g_4 Violence against women

Very bad										Very good
0	1	2	3	4	5	6	7	8	9	10

[PROGRAMMER: DON'T KNOW...888 (GENERATED AUTOMATICALLY IF RESPONDENTS MOVE ON TO THE NEXT QUESTION WITHOUT ANSWERING AND AFTER INSISTING)]

p4h_4 The situation in Catalonia

Very bad										Very good
0	1	2	3	4	5	6	7	8	9	10

[PROGRAMMER: DON'T KNOW...888 (GENERATED AUTOMATICALLY IF RESPONDENTS MOVE ON TO THE NEXT QUESTION WITHOUT ANSWERING AND AFTER INSISTING)]

p37a_4 And how has the economic situation in Spain changed in the last 12 months?

1 It's gotten much worse
2 It's gotten a little worse
3 It's the same
4 It's gotten a little better
5 It's gotten much better

p37b_4 And how has the economic situation of your household changed in the last 12 months?

1 It's gotten much worse
2 It's gotten a little worse
3 It's the same
4 It's gotten a little better
5 It's gotten much better

p76_4 In general, are you very satisfied, quite satisfied, not too satisfied or not at all satisfied with the way democracy works in the European Union?

1. Very satisfied
2. Quite satisfied
3. Not too satisfied
4. Not satisfied at all
5. I don't know
6. I prefer not to answer

p77_4 In general terms, do you consider Spain being a member of the European Union to be a good thing, a bad thing, or neither a good nor a bad thing?

1. A good thing
2. A bad thing
3. Neither a good nor a bad thing
4. I don't know
5. I prefer not to answer

PROGRAMMER: IN THE FOLLOWING QUESTIONS IF RESPONDENTS GO FORWARD WITHOUT ANSWERING, DISPLAY A MESSAGE THAT SAYS "IF YOU WILL GO FORWARD WITHOUT ANSWERING THIS QUESTION, YOUR ANSWER WILL BE RECORDED AS "DON'T KNOW / DON'T ANSWER" (888), DO YOU AGREE?" WITH RESPONSE OPTIONS "YES" AND "NO".

p48_4 Can you tell me with what level of interest you are following the electoral campaign for the European and regional elections?

1. With no interest
2. With little interest
3. With some interest
4. With great interest

p49_4 During this electoral campaign, how often do you follow the political and electoral information through the general information newspapers?

1. Every day or almost every day
2. Several days of the week
3. Only on weekends

4. From time to time
5. Never or hardly ever

p50_4 And how often do you follow political and electoral information on television?

1. Every day or almost every day
2. Several days of the week
3. Only on weekends
4. From time to time
5. Never or hardly ever

p51_4 And how often do you follow political and electoral information through the radio?

1. Every day or almost every day
2. Several days of the week
3. Only on weekends
4. From time to time
5. Never or hardly ever

p52_4 And how often do you follow political and electoral information through the Internet?

1. Every day or almost every day
2. Several days of the week
3. Only on weekends
4. From time to time
5. Never or hardly ever **[SKIP to QUESTION p6_4]**

[PROGRAMMER: To all but those who responded NO INTERNET in the previous question. OTHERISE, CODE AS 999]

p53_4 Which of the following websites have you consulted to date to obtain this political and electoral information? You can choose more than one option

1. Media pages (newspapers, radio, etc.) **[p53a_4]**
2. Party/candidate pages **[p53b_4]**
3. Pages of citizen organizations or civic movements **[p53c_4]**
4. Blogs and discussion forums [p53d_4]
5. Social networks (Facebook, Tuenti, Twitter, etc.) **[p53e_4]**
6. Others____________**[p53f_4]**

888. I don't want to answer **[p53g_4]**

Now we'll talk about aspects of your ideological preferences. Remember again how important it is that you read the questions carefully and choose the answer that best fits what you think and feel. The results and quality of this international research depend on your effort and attention to answer. We remind you that your answers will remain anonymous and will only be treated, along with those of other respondents, in a statistical manner.

p78_4 Some people say that European integration should go further. Others say it has already gone too far. What is your opinion? Please indicate your position using the following 11-point scale where 0 means that European integration "has already gone too far" and 10 means that "it should go further". Which number on this scale best describes your position?

[PROGRAMMER: VERTICAL ORIENTATION ON MOBILE DEVICES]

**It's already gone too far**										**It should go further**
0	1	2	3	4	5	6	7	8	9	10

PROGRAMMER: IN THE FOLLOWING QUESTIONS IF RESPONDENTS GO FORWARD WITHOUT ANSWERING, DISPLAY A MESSAGE THAT SAYS "IF YOU WILL GO FORWARD WITHOUT ANSWERING THIS QUESTION, YOUR ANSWER WILL BE RECORDED AS "DON'T KNOW / DON'T ANSWER" (888), DO YOU AGREE?" WITH RESPONSE OPTIONS "YES" AND "NO".

[PROGRAMMER: DON'T KNOW...888 (GENERATED AUTOMATICALLY IF RESPONDENTS MOVE ON TO THE NEXT QUESTION WITHOUT ANSWERING AND AFTER INSISTING)]

And where would you place each of the following political parties on this same scale?

[PROGRAMMER: RANDOMLY ROTATE THE ORDER OF THE PARTIES FOR EACH RESPONDENT]

[PROGRAMMER: ON MOBILE DEVICES, RANDOMLY ROTATE ORDER FOR EACH RESPONDENT, VERTICAL ORIENTATION AND SEPARATE INTO SCREENS WITH TWO OR THREE ITEMS PER SCREEN].

		It's already gone too far										It should go further	I don't know
**P79a_4**	PP (People's Party)	0	1	2	3	4	5	6	7	8	9	10	888
**P79b_4**	PSOE (Spanish Socialist Workers' Party)	0	1	2	3	4	5	6	7	8	9	10	888
**P79c_4**	Unidas Podemos (En Comú Podem)	0	1	2	3	4	5	6	7	8	9	10	888
**P79e_4**	Ciudadanos (C's - Ciutadans)	0	1	2	3	4	5	6	7	8	9	10	888
**P79f_4**	ERC (Esquerra Republicana de Catalunya)	0	1	2	3	4	5	6	7	8	9	10	888
**P79g_4**	JxCat (Junts per Catalunya)	0	1	2	3	4	5	6	7	8	9	10	888
**P79h_4**	EAJ-PNV (Euzko Alderdi Jeltzalea, Basque Nationalist Party)	0	1	2	3	4	5	6	7	8	9	10	888
**P79i_4**	EH-Bildu (Euskal Herria-Bildu)	0	1	2	3	4	5	6	7	8	9	10	888
**P79n_4**	Coalición Canaria	0	1	2	3	4	5	6	7	8	9	10	888
**P79m_4**	Compromís	0	1	2	3	4	5	6	7	8	9	10	888
**P79o_4**	En Marea	0	1	2	3	4	5	6	7	8	9	10	888
**P79l_4**	Vox	0	1	2	3	4	5	6	7	8	9	10	888

THEY ARE ELIMINATED:
p6_3
p7a_3 TO p7l_3

p8_4 Nowadays, the Autonomous Communities can legislate, together with the Government and the National Legislature, on some aspects of the citizens' daily life, such as health and education. However, not everyone considers that this should be the case.

On this subject, could you tell me where you would position yourself on the following scale from 0 to 10?

[PROGRAMMER: VERTICAL ORIENTATION ON MOBILE DEVICES]

The Spanish Government should regain its powers										The Autonomous Communities should be able to legislate on major issues in citizens' daily lives
0	1	2	3	4	5	6	7	8	9	10

And on this same issue, where do you think the following political parties are positioned on this same scale?

[PROGRAMMER: RANDOMLY ROTATE THE ORDER OF THE PARTIES FOR EACH RESPONDENT]

[PROGRAMMER: ON MOBILE DEVICES, RANDOMLY ROTATE ORDER FOR EACH RESPONDENT, VERTICAL ORIENTATION AND SEPARATE INTO SCREENS WITH TWO OR THREE INDICATORS PER SCREEN].

		**The Spanish Government should regain its powers**										**The Autonomous Communities should be able to legislate on major issues in citizens' daily lives**	**I don't know**
**P9a_4**	PP (People's Party)	0	1	2	3	4	5	6	7	8	9	10	888
**P9b_4**	PSOE (Spanish Socialist Workers' Party)	0	1	2	3	4	5	6	7	8	9	10	888
**P9c_4**	Unidas Podemos (En Comú Podem)	0	1	2	3	4	5	6	7	8	9	10	888
**P9e_4**	Ciudadanos (C's - Ciutadans)	0	1	2	3	4	5	6	7	8	9	10	888
**P9f_4**	ERC (Esquerra Republicana de Catalunya)	0	1	2	3	4	5	6	7	8	9	10	888
**P9g_4**	JxCat (Junts per Catalunya)	0	1	2	3	4	5	6	7	8	9	10	888
**P9h_4**	EAJ-PNV (Euzko Alderdi Jeltzalea, Basque Nationalist Party)	0	1	2	3	4	5	6	7	8	9	10	888
**P9i_4**	EH-Bildu (Euskal Herria-Bildu)	0	1	2	3	4	5	6	7	8	9	10	888
**P9n_4**	Coalición Canaria	0	1	2	3	4	5	6	7	8	9	10	888
**P9m_4**	Compromís	0	1	2	3	4	5	6	7	8	9	10	888
**P9o_4**	En Marea	0	1	2	3	4	5	6	7	8	9	10	888
**P9l_4**	Vox	0	1	2	3	4	5	6	7	8	9	10	888

Now we would like to know your opinion on some national issues that are the subject of public debate. Please indicate your response on a scale from 0 to 10

[PROGRAMMER: RANDOMLY ROTATE TOPICS p10a_4 to p10h_4 FOR EACH RESPONDENT, VERTICAL ORIENTATION ON MOBILE DEVICES]

p10a_4 Would you say that, in general, immigrants have to adapt to the customs of Spain and their region or that they should be able to maintain their customs despite living in another country?

**They have to adapt to the customs of Spain**									**They should be able to keep their customs**
0	1	2	3	4	5	6	7	8	9	10

p10b_4 And, do you think that private initiative (private companies) or state intervention is the best way to solve the problems of the Spanish economy?

**Private initiative is the best way**									**State intervention is the best way**

0	1	2	3	4	5	6	7	8	9	10

p10c_4 Would you say that same-sex marriages should be prohibited or allowed by law?

They should be forbidden by law										They should be allowed by law
0	1	2	3	4	5	6	7	8	9	10

p10d_4 And, do you think that main public services should be provided by private companies or by public institutions of the State?

They should be carried out by private companies										They should be carried out by public institutions
0	1	2	3	4	5	6	7	8	9	10

p10e_4 Would you say that women should have the right to abortion?

Women should not have the right to abortion										Women should have the right to abortion
0	1	2	3	4	5	6	7	8	9	10

p10f_4 Would you say that income and wealth are distributed fairly among regular people in Spain or that wealth should be redistributed more fairly?

Wealth is fairly distributed										Wealth should be redistributed more fairly
0	1	2	3	4	5	6	7	8	9	10

p10g_4 And, do you think a woman should be prepared to give up her job for the sake of her family or should she be able to work?

She should be prepared to quit her job for the sake of her family										She should be able to work
0	1	2	3	4	5	6	7	8	9	10

p10h_4 Would you say that immigration to Spain should be reduced or increased?

Immigration to Spain should be reduced										Immigration to Spain should be increased
0	1	2	3	4	5	6	7	8	9	10

p10i_4 Would you say that the solution for Catalonia…

… is through the rapid application of the Constitutional Article 155										… is through granting the right of self-determination with a referendum
0	1	2	3	4	5	6	7	8	9	10

FOR QUESTIONS p10a_4 TO p10h_4 PROGRAMMER: DON'T KNOW...888 (GENERATED AUTOMATICALLY IF RESPONDENTS MOVE ON TO THE NEXT QUESTION WITHOUT ANSWERING AND AFTER INSISTING)

FOR QUESTIONS p10a_4 TO p10h_4 PROGRAMMER: IF RESPONDENTS FO FORWARD WITHOUT ANSWERING, DISPLAY A MESSAGE THAT SAYS "IF YOU ADVANCE WITHOUT ANSWERING THIS QUESTION, YOUR ANSWER WILL BE RECORDED AS "DON'T KNOW / DON'T ANSWER", DO YOU AGREE?" WITH RESPONSE OPTIONS "YES" AND "NO".

We would also like to know your feelings about some groups of people in Spanish society, using this thermometer.

Ratings between 60 and 100 mean that you have rather favourable feelings toward that group of people, with 100 being very favourable; while ratings between 0 and 40 mean instead that you have no favourable feelings toward the group, with 0 being very unfavourable. If you do not have particularly favourable or unfavourable feelings toward a group, you should choose a 50 grade rating.

[PROGRAMMER: ROTATE THE ORDER OF QUESTIONS RANDOMLY WITHIN EACH GROUP]

[SOCIAL GROUPS]

[PROGRAMMER: ON MOBILE DEVICES, ROTATE RANDOM MODE ORDER FOR EACH RESPONDENT, VERTICAL ORIENTATION AND SEPARATE INTO SCREENS WITH TWO OR THREE ITEMS PERSCREEN].

		Unfavourable feelings				No feelings				Favourable feelings
**p11a_4**	The Basques	0	15	30	40	50	60	70	85	100
**p11b_4**	The Catalans	0	15	30	40	50	60	70	85	100
**p11c_4**	The people of Madrid	0	15	30	40	50	60	70	85	100
**p11d_4**	The Andalusians	0	15	30	40	50	60	70	85	100
**p11e_4**	Refugees	0	15	30	40	50	60	70	85	100
**p11s_4**	Immigrants	0	15	30	40	50	60	70	85	100

[VOTING GROUP]

And what about these groups of people?

[PROGRAMMER: ON MOBILE DEVICES, ROTATE RANDOM MODE ORDER FOR EACH RESPONDENT, VERTICAL ORIENTATION AND SEPARATE INTO SCREENS WITH TWO OR THREE ITEMS PERSCREEN].

		Unfavourable feelings				No feelings				Favourable feelings
**p11f_4**	The PP voters	0	15	30	40	50	60	70	85	100
**p11g_4**	PSOE voters	0	15	30	40	50	60	70	85	100
**p11h_4**	Ciudadanos voters	0	15	30	40	50	60	70	85	100
**p11i_4**	Unidas Podemos voters	0	15	30	40	50	60	70	85	100
**p11r_4**	Vox voters	0	15	30	40	50	60	70	85	100

[LEADERSHIP GROUP]

And what are your feelings about these leaders?

[PROGRAMMER: ON MOBILE DEVICES, ROTATE RANDOM MODE ORDER FOR EACH RESPONDENT, VERTICAL ORIENTATION AND SEPARATE INTO SCREENS WITH TWO OR THREE ITEMS PERSCREEN].

		Unfavourable feelings				No feelings				Favourable feelings
**p11j_4**	Pablo Casado	0	15	30	40	50	60	70	85	100
**p11k_4**	Pedro Sanchez	0	15	30	40	50	60	70	85	100
**p11l_4**	Albert Rivera	0	15	30	40	50	60	70	85	100
**p11m_4**	Pablo Iglesias	0	15	30	40	50	60	70	85	100
**p11n_4**	Iñigo Urkullu	0	15	30	40	50	60	70	85	100
**p11o_4**	Carles Puigdemont	0	15	30	40	50	60	70	85	100
**p11p_4**	Oriol Junqueras	0	15	30	40	50	60	70	85	100
**P11q_4**	Santiago Abascal	0	15	30	40	50	60	70	85	100

Now we would like to know how much you trust various groups of people. For each, indicate the extent to which you trust the people in that group on a scale of 0 to 10.

[PROGRAMMER: RANDOMLY ROTATE THE ORDER OF QUESTIONS FOR EACH RESPONDENT]

[PROGRAMMER: ON MOBILE DEVICES, RANDOMLY ROTATE ORDER FOR EACH RESPONDENT, VERTICAL ORIENTATION AND SEPARATE INTO SCREENS WITH TWO OR THREE ITEMS PER SCREEN].

		I don't trust at all										I trust completely
p13a_4	Your Family	0	1	2	3	4	5	6	7	8	9	10
p13b_4	Your neighbours	0	1	2	3	4	5	6	7	8	9	10
p13c_4	People you know personally	0	1	2	3	4	5	6	7	8	9	10
p13d_4	People you meet for the first time	0	1	2	3	4	5	6	7	8	9	10
p13e_4	People of another religion	0	1	2	3	4	5	6	7	8	9	10
p13f_4	People of other nationalities	0	1	2	3	4	5	6	7	8	9	10
p13g_4	The Catalans	0	1	2	3	4	5	6	7	8	9	10
p13h_4	The Basques	0	1	2	3	4	5	6	7	8	9	10
p13i_4	People from Madrid	0	1	2	3	4	5	6	7	8	9	10
p13j_4	People from Andalusia	0	1	2	3	4	5	6	7	8	9	10
p13k_4	Immigrants residing in our country	0	1	2	3	4	5	6	7	8	9	10

FOR QUESTIONS p13a_4 TO p13k_4 [PROGRAMMER: DON'T KNOW...888 (AUTOMATICALLY GENERATED IF RESPONDENTS MOVE ON TO THE NEXT QUESTION WITHOUT ANSWERING AND AFTER INSISTING)

PROGRAMMER: DON'T KNOW...888 (GENERATED AUTOMATICALLY IF RESPONDENTS GO FORWARD WITHOUT ANSWERING AND AFTER INSISTING)]

FOR QUESTIONS p13A_4 TO p13J_4 [PROGRAMMER: IF RESPONDENTS GO FORWARD WITHOUT ANSWERING, DISPLAY A MESSAGE THAT SAYS "IF YOU ADVANCE WITHOUT ANSWERING ONE OF THE QUESTIONS ON THIS SCREEN, YOUR ANSWER WILL BE RECORDED AS "DON'T KNOW / DON'T ANSWER" FOR THAT QUESTION, DO YOU AGREE?" WITH RESPONSE OPTIONS "YES" AND "NO".]

How much do you trust various citizen groups? For each, indicate the extent to which you trust the people in that group on a scale from 0 to 10.

[PROGRAMMER: RANDOMLY ROTATE THE ORDER OF QUESTIONS FOR EACH RESPONDENT]

[PROGRAMMER: ON MOBILE DEVICES, RANDOMLY ROTATE ORDER FOR EACH RESPONDENT, VERTICAL ORIENTATION AND SEPARATE INTO SCREENS WITH TWO OR THREE ITEMS PER SCREEN].

		I don't trust it at all										I have every confidence
p14a_4	PP voters	0	1	2	3	4	5	6	7	8	9	10
p14b_4	PSOE voters	0	1	2	3	4	5	6	7	8	9	10
p14c_4	Ciudadanos voters	0	1	2	3	4	5	6	7	8	9	10
p14d_4	Unidas Podemos voters	0	1	2	3	4	5	6	7	8	9	10
p14e_4	ERC voters	0	1	2	3	4	5	6	7	8	9	10
p14f_4	Junts per Catalunya voters	0	1	2	3	4	5	6	7	8	9	10
p14g_4	EAJ-PNV voters	0	1	2	3	4	5	6	7	8	9	10
p14h_4	Vox voters	0	1	2	3	4	5	6	7	8	9	10

FOR QUESTIONS p14a_4 TO p14h_4 [PROGRAMMER: DON'T KNOW...888 (AUTOMATICALLY GENERATED IF YOU SKIP WITHOUT ANSWERING AND AFTER INSISTING)]

FOR QUESTIONS p14a_4 TO p14h_4 [PROGRAMMER: IF RESPONDENTS GO FORWARD WITHOUT ANSWERING, DISPLAY A MESSAGE THAT SAYS "YOU WILL ADVANCE WITHOUT ANSWERING ONE OF THE QUESTIONS ON THIS SCREEN AND YOUR ANSWER WILL BE RECORDED AS "DON'T KNOW / DON'T ANSWER" FOR THAT QUESTION, DO YOU AGREE?" AND AS ANSWERS "YES" AND "NO".]

IMPORTANT: ON MOBILE DEVICES ORIENTATION MUST BE HORIZONTAL

[PROGRAMMER: ROTATE QUESTIONS RANDOMLY AND PRESENT THEM SEPARATELY ON SCREEN]

[IMPORTANT: ON MOBILE DEVICES ORIENTATION MUST BE HORIZONTAL]

Could you please tell us on a scale of 1 to 7, where 1 is "I don't trust at all" and 7 is "I completely trust", how much you trust each of the following political institutions...

[PROGRAMMER: PUT ON NEW SCREEN]

trust1a_4 ...the Spanish Parliament

I don't trust at all						I completely trust
1	2	3	4	5	6	7

trust1b_4 ...the Spanish government

[PROGRAMMER: PUT ON NEW SCREEN]

I don't trust at all						I completely trust
1	2	3	4	5	6	7

trust1c_4 ...the Parliament of **[PROGRAMMER: NAME CCAA]**

[PROGRAMMER: PUT ON NEW SCREEN]

I don't trust at all						I completely trust
1	2	3	4	5	6	7

trust1d_4 ...the government of **[PROGRAMMER: NAME CCAA]**

[PROGRAMMER: PUT ON NEW SCREEN]

I don't trust at all						I completely trust
1	2	3	4	5	6	7

trust1e_4 ...the politicians in Spain

[PROGRAMMER: PUT ON NEW SCREEN]

I don't trust at all						I completely trust
1	2	3	4	5	6	7

trust1f_4 ...the political parties in Spain

[PROGRAMMER: PUT ON NEW SCREEN]

I don't trust at all						I completely trust
1	2	3	4	5	6	7

trust1g_4 ...the Spanish police

[PROGRAMMER: PUT ON NEW SCREEN]

I don't trust at all						I completely trust
1	2	3	4	5	6	7

trust1h_4 ...the Spanish judicial system

[PROGRAMMER: PUT ON NEW SCREEN]

I don't trust at all						I completely trust
1	2	3	4	5	6	7

trust1i_4 ...the European Parliament

[PROGRAMMER: PUT ON NEW SCREEN]

I don't trust at all						I completely trust
1	2	3	4	5	6	7

trust1j_4 ...the government of the European Union (The European Commission)

[PROGRAMMER: PUT ON NEW SCREEN]

I don't trust at all						I completely trust
1	2	3	4	5	6	7

p12a_4 Would you say that, in general, you can trust most people or that you can never be too careful in dealing with others? Please place yourself on the following scale from 0 to 10.

[PROGRAMMER: ON MOBILE DEVICES, VERTICAL ORIENTATION]

You can never be too careful										Most people can be trusted
0	1	2	3	4	5	6	7	8	9	10

p12b_4 And do you think that most people would try to take advantage of you if they could, or that they would be honest with you?

[PROGRAMMER: ON MOBILE DEVICES, VERTICAL ORIENTATION]

Most people would try to take advantage of me										Most people would be honest with me
0	1	2	3	4	5	6	7	8	9	10

p12c_4 Would you say that most of the time people try to help others or that they mainly look out for themselves?

[PROGRAMMER: ON MOBILE DEVICES, VERTICAL ORIENTATION]

**Most of the time people look out for themselves**										**Most of the time people try to help others**
0	1	2	3	4	5	6	7	8	9	10

I would now ask you to pay attention to this list of political leaders and tell me in each case whether you know them and how you would assess their political activity on the following scale:

[PROGRAMMER: ROTATE ORDER OF QUESTIONS AT RANDOM]

p40a_4 Pablo Casado

1 I know him
2 I don't know him **[PROGRAMMER: GO TO NEXT CANDIDATE AND THE NEXT QUESTION IS 999 MISSING]**

p41a_4

[PROGRAMMER: VERTICAL ORIENTATION ON MOBILE DEVICES]

**Very badly**										**Very good**
0	1	2	3	4	5	6	7	8	9	10

p40b_4 Pedro Sánchez

1 I know him
2 I don't know him **[PROGRAMMER: GO TO NEXT CANDIDATE AND THE NEXT QUESTION IS 999 MISSING]**

p41b_4

[PROGRAMMER: VERTICAL ORIENTATION ON MOBILE DEVICES]

**Very badly**										**Very good**
0	1	2	3	4	5	6	7	8	9	10

p40c_4 Albert Rivera

1 I know him
2 I don't know him **[PROGRAMMER: GO TO NEXT CANDIDATE AND THE NEXT QUESTION IS 999 MISSING]**

p41c_4

[PROGRAMMER: VERTICAL ORIENTATION ON MOBILE DEVICES]

**Very badly**										**Very good**
0	1	2	3	4	5	6	7	8	9	10

p40d_4 Pablo Iglesias

1 I know him
2 I don't know him **[PROGRAMMER: GO TO NEXT CANDIDATE AND THE NEXT QUESTION IS 999 MISSING]**

p41d_4

[PROGRAMMER: VERTICAL ORIENTATION ON MOBILE DEVICES]

Very badly										Very good
0	1	2	3	4	5	6	7	8	9	10

p40e_4 Alberto Garzón

1 I know him
2 I don't know him **[PROGRAMMER: GO TO NEXT CANDIDATE AND THE NEXT QUESTION IS 999 MISSING]**

p41e_4

[PROGRAMMER: VERTICAL ORIENTATION ON MOBILE DEVICES]

Very badly										Very good
0	1	2	3	4	5	6	7	8	9	10

p40i_4 Santiago Abascal [PROGRAMMER: ASK ONLY IN CATALONIA]

1 I know him
2 I don't know him **[PROGRAMMER: GO TO NEXT CANDIDATE AND THE NEXT QUESTION IS 999 MISSING]**

p41i_4

[PROGRAMMER: VERTICAL ORIENTATION ON MOBILE DEVICES]

Very badly										Very good
0	1	2	3	4	5	6	7	8	9	10

p40f_4 Iñigo Urkullu [PROGRAMMER: ASK ONLY IN THE BASQUE COUNTRY]

1 I know him
2 I don't know him **[PROGRAMMER: GO TO NEXT CANDIDATE AND THE NEXT QUESTION IS 999 MISSING]**

p41f_4

[PROGRAMMER: VERTICAL ORIENTATION ON MOBILE DEVICES]

Very badly										Very good
0	1	2	3	4	5	6	7	8	9	10

p40g_4 Carles Puigdemont [PROGRAMMER: ASK ONLY IN CATALONIA]

1 I know him
2 I don't know him **[PROGRAMMER: GO TO NEXT CANDIDATE and the next question is 999 missing]**

p41g_4

[PROGRAMMER: VERTICAL ORIENTATION ON MOBILE DEVICES]

Very badly										Very good
0	1	2	3	4	5	6	7	8	9	10

p40h_4 Oriol Junqueras [PROGRAMMER: ASK ONLY IN CATALONIA]

1 I know him
2 I don't know him **[PROGRAMMER: GO TO NEXT CANDIDATE AND THE NEXT QUESTION IS 999 MISSING]**

p41h_4

[PROGRAMMER: VERTICAL ORIENTATION ON MOBILE DEVICES]

Very badly										Very good
0	1	2	3	4	5	6	7	8	9	10

Remember that this battery was asked in the third wave, but since there are several questions for each of the four leaders, in the third wave only two batteries of two leaders were asked per respondent. In this wave, each respondent must be assigned the battery of leaders that was not asked in the third wave.

[PROGRAMMER: IF IN THE PREVIOUS BATTERY THE RESPONDENT SAYS IN p40a_4, p40b_4p, p40c_4, p40d_4 NOT TO KNOW THE LEADER, DO NOT MAKE THE NEXT BATTERY CORRESPONDING TO THAT LEADER. IN THAT CASE AUTOMATICALLY CODE ALL THESE QUESTIONS AS 888, I DON'T KNOW.]

[BATTERY CASADO]

We'd like to know what you think of Pablo Casado. Read the list of adjectives below and tell us how much you agree or disagree that each characteristic describes him.

**PROGRAMMER: RANDOMLY ROTATE THE RESPONSE CATEGORIES**		Strongly agree	Somewhat agree	Neither agree nor disagree	Somewhat disagree	Strongly disagree
**p54a_4**	Decisive	1	2	3	4	5
**p54b_4**	Intelligent	1	2	3	4	5
**p54c_4**	Charismatic	1	2	3	4	5
**p54d_4**	Incompetent	1	2	3	4	5
**p54e_4**	Caring	1	2	3	4	5
**p54f_4**	Reliable	1	2	3	4	5
**p54g_4**	Dishonest	1	2	3	4	5
**p54h_4**	Arrogant	1	2	3	4	5

p55_4 To what extent do you consider that the personal characteristics above that you believe define Pablo Casado define you as well?

1 A lot
2 A fair amount
3 Somewhat
4 A little
5 Not at all

p56_4 To what extent do you admire the qualities that define Pablo Casado?

1 A lot
2 A fair amount
3 Somewhat
4 A little
5 Not at all

To what extent do you agree or disagree with the following statements?

**PROGRAMMER: RANDOMLY ROTATE**	Strongly	Somewhat	Neither agree nor	Somewhat	Strongly disagree

THE RESPONSE CATEGORIES		agree	agree	disagree	disagree	
**p57a_4**	I identify with those who express support for Pablo Casado	1	2	3	4	5
**p57b_4**	I identify with Pablo Casado	1	2	3	4	5
**p57c_4**	Pablo Casado shares my beliefs or convictions	1	2	3	4	5
**p57d_4**	I have no problem telling people that I voted for Pablo Casado	1	2	3	4	5
**p57e_4**	Pablo Casado represents the ideals of his party	1	2	3	4	5

[BATTERY SÁNCHEZ]

We'd like to know what you think of Pedro Sánchez. Read the list of adjectives below and tell us how much you agree or disagree that each characteristic describes him.

**PROGRAMMER: RANDOMLY ROTATE THE RESPONSE CATEGORIES**		Strongly agree	Somewhat agree	Neither agree nor disagree	Somewhat disagree	Strongly disagree
**p58a_4**	Decisive	1	2	3	4	5
**p58b_4**	Intelligent	1	2	3	4	5
**p58c_4**	Charismatic	1	2	3	4	5
**p58d_4**	Incompetent	1	2	3	4	5
**p58e_4**	Caring	1	2	3	4	5
**p58f_4**	Reliable	1	2	3	4	5
**p58g_4**	Dishonest	1	2	3	4	5
**p58h_4**	Arrogant	1	2	3	4	5

p59_4 To what extent do you consider that the personal characteristics above that you think define Pedro Sánchez define you as well?

1 A lot
2 A fair amount
3 Somewhat
4 A little
5 Not at all

p60_4 To what extent do you admire the qualities that define Pedro Sánchez?

1 A lot
2 A fair amount
3 Somewhat
4 A little
5 Not at all

To what extent do you agree or disagree with the following statements?

PROGRAMMER: RANDOMLY ROTATE THE RESPONSE CATEGORIES		Strongly agree	Somewhat agree	Neither agree nor disagree	Somewhat disagree	Strongly disagree
**p61a_4**	I identify with those who express support for Pedro Sánchez	1	2	3	4	5
**p61b_4**	I identify with Pedro Sánchez	1	2	3	4	5
**p61c_4**	Pedro Sánchez shares my beliefs or convictions	1	2	3	4	5
**p61d_4**	I have no problem telling people that I voted for Pedro Sánchez	1	2	3	4	5
**p61e_4**	Pedro Sánchez represents the ideals of his party	1	2	3	4	5

[BATTERY RIVERA]

We'd like to know what you think of Albert Rivera. Read the list of adjectives below and tell us how much you agree or disagree that each characteristic describes him.

PROGRAMMER: RANDOMLY ROTATE THE RESPONSE CATEGORIES		Strongly agree	Somewhat agree	Neither agree nor disagree	Somewhat disagree	Strongly disagree
**p62a_4**	Decisive	1	2	3	4	5
**p62b_4**	Intelligent	1	2	3	4	5
**p62c_4**	Charismatic	1	2	3	4	5
**p62d_4**	Incompetent	1	2	3	4	5
**p62e_4**	Caring	1	2	3	4	5
**p62f_4**	Reliable	1	2	3	4	5
**p62g_4**	Dishonest	1	2	3	4	5
**p62h_4**	Arrogant	1	2	3	4	5

p63_4 To what extent do you consider that the personal characteristics above that you think define Albert Rivera define you as well?

1 A lot
2 A fair amount
3 Somewhat
4 A little
5 Not at all

p64_4 To what extent do you admire the qualities that define Albert Rivera?

1 A lot
2 A fair amount
3 Somewhat
4 A little
5 Not at all

To what extent do you agree or disagree with the following statements?

**PROGRAMMER: RANDOMLY ROTATE THE RESPONSE CATEGORIES**		Strongly agree	Somewhat agree	Neither agree nor disagree	Somewhat disagree	Strongly disagree
**p65a_4**	I identify with those who express support for Albert Rivera	1	2	3	4	5
**p65b_4**	I identify with Albert Rivera	1	2	3	4	5
**p65c_4**	Albert Rivera shares my beliefs or convictions	1	2	3	4	5
**p65d_4**	I have no problem telling people that I voted for Albert Rivera	1	2	3	4	5
**p65e_4**	Albert Rivera represents the ideals of his party	1	2	3	4	5

[BATTERY IGLESIAS]

We'd like to know what you think of Pablo Iglesias. Read the list of adjectives below and tell us how much you agree or disagree that each characteristic describes him.

**PROGRAMMER: RANDOMLY ROTATE THE RESPONSE CATEGORIES**		Strongly agree	Somewhat agree	Neither agree nor disagree	Somewhat disagree	Strongly disagree
**p66a_4**	Decisive	1	2	3	4	5
**p66b_4**	Intelligent	1	2	3	4	5
**p66c_4**	Charismatic	1	2	3	4	5
**p66d_4**	Incompetent	1	2	3	4	5
**p66e_4**	Caring	1	2	3	4	5
**p66f_4**	Reliable	1	2	3	4	5
**p66g_4**	Dishonest	1	2	3	4	5
**p66h_4**	Arrogant	1	2	3	4	5

p67_4 To what extent do you consider that the personal characteristics above that you believe define Pablo Iglesias   define you as well?

1 A lot
2 A fair amount
3 Somewhat
4 A little
5 Not at all

p68_4 To what extent do you admire the qualities that define Pablo Iglesias?

1 A lot
2 A fair amount
3 Somewhat
4 A little

5 Not at all

To what extent do you agree or disagree with the following statements?

PROGRAMMER: RANDOMLY ROTATE THE RESPONSE CATEGORIES		Strongly agree	Somewhat agree	Neither agree nor disagree	Somewhat disagree	Strongly disagree
**p69a_4**	I identify with those who express support for Pablo Iglesias	1	2	3	4	5
**p69b_4**	I identify with Pablo Iglesias	1	2	3	4	5
**p69c_4**	Pablo Iglesias shares my beliefs or convictions	1	2	3	4	5
**p69d_4**	I have no problem telling people that I voted for Pablo Iglesias	1	2	3	4	5
**p69e_4**	Pablo Iglesias represents the ideals of his party	1	2	3	4	5

FINAL PART LEADERSHIP BRAND

[PROGRAMMER: IF RESPONDENTS GO FORWARD WITHOUT ANSWERING, DISPLAY A MESSAGE THAT SAYS "if YOU ADVANCE WITHOUT ANSWERING THIS QUESTION, YOUR ANSWER WILL BE RECORDED AS "DON'T KNOW / DON'T ANSWER" FOR THAT QUESTION, DO YOU AGREE?" WITH RESPONSE OPTIONS "YES" AND "NO".]

Do you have an account on any of the following social networks?

		Yes	No
**p19b_4**	Facebook	1	0
**p19c_4**	Google +	1	0
**p19d_4**	LinkedIn	1	0
**p19e_4**	Instagram	1	0
**p19f_4**	Flickr	1	0
**p19g_4**	YouTube	1	0
**p19i_4**	WhatsApp	1	0
**p19h_4**	Other	1	0

[PROGRAMMER: IF RESPONDENT SAYS "NO" ON p19a_4 p19b_4, p19d_4 or p19i_4 GO TO p35_4]

p26b_4 How often do you discuss politics or current political issues on social networks, Facebook, Twitter or any other blog?

[PROGRAMMER: ASK ONLY THOSE WHO ANSWERED 1/YES ON ONE OF THE p19a_4-p19h_4; PUT 999 IF THEY SAY NO TO ALL OF THEM]

1 Less than once a month
2 Once a month
3 Several times a month
4 Once a week
5 Several times a week
6 Every day
0 Never **[GO TO p35_4]**
[PROGRAMMER: IN THIS CASE, AUTOMATICALLY GENERATE 999 FOR QUESTIONS p27b_4 to p29b_4]

p27b_4 How often do you agree with the views of the people with whom you talk about politics in these forums?

3 Always
2 Many times
1 Sometimes
0 Never
888 don't know/don't answer

p28b_4 Also, how often do you disagree with the views of the people with whom you talk about politics in these forums?

3 Always
2 Many times
1 Sometimes
0 Never
888 don't know/don't answer

p29b_4 Do you think that the people you talk to about politics in these forums...

3 They support the same party as you
2 They divide their support among different parties
1 They support a different party than yours
0 They don't support any party
888 don't know/don't answer

PROGRAMMER: THIS QUESTION IS ONLY FOR THOSE WHO HAVE ANSWERED YES (1) TO SOME OF THE SOCIAL NETWORKS IN p19a, p19b, p19d, p19e and p19f

How often have you seen political information on Twitter or other social networks coming from... ?

	Every day or almost every day	Several days a week	Only on weekends	From time to time	Never or hardly ever
**p70a_4 Family and friends**	1	2	3	4	5
**p70b_4 Political parties and candidates**	1	2	3	4	5
**p70c_4 Media**	1	2	3	4	5
**p70d_4 Journalists**	1	2	3	4	5
**p70e_3 Celebrit ies**	1	2	3	4	5

[PROGRAMMER: THIS QUESTION IS ONLY FOR THOSE WHO HAVE ANSWERED YES (1) to some of the social networks in p19a, p19b, p19d, p19e and p19f.]

How much do you trust the information shared on social networks from...?

	Completely	Somewhat	Neither a little nor a lot	Little	Not at all
**p71a_4** Family and friends	1	2	3	4	5
**p71b_4** Political parties and candidates	1	2	3	4	5
**p71c_4** Media	1	2	3	4	5
**p71d_4** Journalists	1	2	3	4	5
**p71e_4** Celebrities	1	2	3	4	5

Now we'll talk about aspects of your political preferences. Remember again the importance of reading the questions carefully and choosing the answer that best fits your thoughts and opinions. The results and quality of this international research depend on your efforts and attention to detail. We remind you that your answers will remain anonymous and will only be treated, along with those of other respondents, in a statistical manner.

p35_4 Do you consider yourself close to any political party?

1 Yes **[GO TO p35a_4]**
0 No **[GO TO s8_4]**

p35a_4 Which one?

[PROGRAMMER: ASK IF 1 ON p35_4. CODE 999 TO THE OTHERS]

1 PP (Popular Party)
2 PSOE (Spanish Socialist Workers' Party)
3 Podemos and other affiliated municipal lists (En Comú Podem, Ahora Madrid)
4 IU (United Left)
5 Ciudadanos (C's - Ciutadans)
6 ERC (Esquerra Republicana de Catalunya)
7 JxCat (Junts per Catalunya)
8 EAJ - PNV (Euzko Alderdi Jeltzalea - Basque Nationalist Party)
9 EH - Bildu (Euskal Herria - Bildu)
11 CC (Canary Islands Coalition)
13 VOX
12 Others___________

p35b_4 And how close do you feel to this party?

[PROGRAMMER: ASK IF 1 ON p35_4. PUT 999 TO THE OTHERS]

3 Very close

2 Somewhat close
1 Not very close
0 Not at all close

I would now like to ask you what you think of the political parties that have the most electoral support. Please rate each of them on this scale from 0 to 10 where 0 means that you don't like the party at all, and 10 means that you like it very much.

		**I don't like it at all**										**I like it very much**	**I don't know**
**p72a_4**	PP (People's Party)	0	1	2	3	4	5	6	7	8	9	10	888
**p72b_4**	PSOE (Spanish Socialist Workers' Party)	0	1	2	3	4	5	6	7	8	9	10	888
**p72c_4**	Unidas Podemos (En Comú Podem)	0	1	2	3	4	5	6	7	8	9	10	888
**p72e_4**	Ciudadanos (C's - Ciutadans)	0	1	2	3	4	5	6	7	8	9	10	888
**p72f_4**	ERC (Esquerra Republicana de Catalunya)	0	1	2	3	4	5	6	7	8	9	10	888
**p72g_4**	JxCat (Junts per Catalunya)	0	1	2	3	4	5	6	7	8	9	10	888
**p72h_4**	EAJ-PNV (Euzko Alderdi Jeltzalea, Basque Nationalist Party)	0	1	2	3	4	5	6	7	8	9	10	888
**p72i_4**	EH-Bildu (Euskal Herria- Bildu)	0	1	2	3	4	5	6	7	8	9	10	888
**p72l_4**	Vox	0	1	2	3	4	5	6	7	8	9	10	888
**FROM HERE, ONLY TO THE RESPECTIVE AUTONOMOUS COMMUNITIES**													
**p72p_4**	FAC (Citizens' Forum) **ASTURIAS**	0	1	2	3	4	5	6	7	8	9	10	888
**p72n_4**	CC (Canarian Coalition) **CANARY ISLANDS**	0	1	2	3	4	5	6	7	8	9	10	888
**p72m_4**	Compromís **VALENCIAN C.**	0	1	2	3	4	5	6	7	8	9	10	888
**p72o_4**	En Marea **GALICIA**	0	1	2	3	4	5	6	7	8	9	10	888

p73_4 Now, we would like you to tell us what is the probability that you will vote in the next election on May 26th, using a scale from 0 to 10, where 0 means "you will definitely not vote" and 10 means "you will definitely vote".

**You're definitely not going to vote**										**You're definitely going to vote**
00	01	02	03	04	05	06	07	08	09	10

There are many political parties in Spain that would like to count on your vote. What is the probability that you will ever vote for one of the following political parties? Please specify your opinion on a scale from 0 to 10, where 0 means "Not at all likely" and 10 means "Very likely".

		Not at all likely										Very likely	I don't know
**p74a_4**	PP (People's Party)	00	01	02	03	04	05	06	07	08	09	10	888
**p74b_4**	PSOE (Spanish Socialist Workers' Party)	00	01	02	03	04	05	06	07	08	09	10	888
**p74c_4**	Unidas Podemos (En Comú Podem)	00	01	02	03	04	05	06	07	08	09	10	888
**p74e_4**	Ciudadanos (C's - Ciutadans)	00	01	02	03	04	05	06	07	08	09	10	888
**p74f_4**	ERC (Esquerra Republicana de Catalunya)	00	01	02	03	04	05	06	07	08	09	10	888
**p74g_4**	JxCat (Junts per Catalunya)	00	01	02	03	04	05	06	07	08	09	10	888
**p74h_4**	EAJ-PNV (Euzko Alderdi Jeltzalea, Basque Nationalist Party)	00	01	02	03	04	05	06	07	08	09	10	888
**p74i_4**	EH-Bildu (Euskal Herria- Bildu)	00	01	02	03	04	05	06	07	08	09	10	888
**p74l_4**	Vox	00	01	02	03	04	05	06	07	08	09	10	888
	**FROM HERE, ONLY TO THE RESPECTIVE AUTONOMOUS COMMUNITIES**												
**p74p_4**	FAC (Citizens' Forum) **ASTURIAS**	00	01	02	03	04	05	06	07	08	09	10	888
**p74n_4**	CC (Canarian Coalition) **CANARY ISLANDS**	00	01	02	03	04	05	06	07	08	09	10	888
**p74m_4**	Compromís **VALENCIAN C.**	00	01	02	03	04	05	06	07	08	09	10	888
**p74o_4**	En Marea **GALICIA**	00	01	02	03	04	05	06	07	08	09	10	888
**p74q_4**	Partido Regionalista de Cantabria (PRC) **CANTABRIA**	00	01	02	03	04	05	06	07	08	09	10	888

p80_4. As you may already know, elections for the European Parliament will be held the upcoming 26th of May. In these elections, for which party or coalition do you intend to vote?

1. Partido Socialista Obrero Español (PSOE)
2. Partido Popular (PP)
3. Ciudadanos-Partido de la ciudadanía
4. Unidas Podemos Cambiar Europa (Podemos e Izquierda Unida)
5. VOX
6. Partido Animalista contra el Maltrato Animal (PACMA)
7. Ahora Repúblicas (ERC+EH Bildu+BNG+AA+AC)
8. Coalición por una Europa Solidaria. CEUS (EAJ-PNV+CCa-PNC+CxG+The PI+DV+GBAI)
9. Junts
10. Compromís per Europa/Compromiso por Europa. CPE (Commitment+En Marea+Nueva Canarias+Més per Mallorca+Chunta Aragonesista+Tierra Comunera+Iniciativa del Pueblo Andaluz+Izquierda Andalucista+Greens for Europe)
11. Another party or coalition, which one?_______________
12. Null vote
13. White Vote
14. I will not vote
15. I haven't decided yet

888. I don't know

999. I don't answer

[PROGRAMMER IF RESPONDENT SKIPS THE QUESTION WITHOUT ANSWERING, CODE 888]

[PROGRAMMER: ONLY IN ARAGON, ASTURIAS, BALEARIC ISLANDS, CANARY ISLANDS, CANTABRIA, CASTILE AND LEON, CASTILE-LA MANCHA, EXTREMADURA, LA RIOJA, MADRID, MURCIA, NAVARRA]

p81_4 As you may already know, on May 26th there will also be regional elections in your community. In these elections, for which party or coalition do you plan to vote?

1. PSOE
2. PP
3. Ciudadanos
4. Unidas Podemos
5. Podemos
6. Izquierda Unida
7. Equo
8. VOX
9. PACMA
10. Navarra Suma
11. Geroa Bai
12. EH Bildu
13. Coalición Canária
14. Nueva Canarias
15. Foro de Ciudadanos (FAC)
16. CHA (Chunta Aragonesista)
17. Partido Aragonés (PAR)
16. PI (Proposta per les Illes Balears)
17. Més per Mallorca-Més per Menorca (PSM-Entesa)
18. PRC (Partido Regionalista de Cantabria)
19. UPL (Unión del Pueblo Leonés)
20. Partido Riojano (PR+)
21. Más Madrid
22. Another party or coalition, which one?_______________________
23. Null vote
24. White vote
25. I will not vote
26. I haven't decided yet

888. I don't know
999. I don't answer

[PROGRAMMER: IF RESPONDENT SKIPS THE QUESTION WITHOUT ANSWERING, CODE 999]

s8_4 And which best describes your employment situation in the last seven days? Please choose only one of the following options.

1 Employed, but on temporary leave (includes temporary maternity/paternity leave, accident, illness or holidays).
2. Employed, self-employed, or in a family business
2 Studying, even if you have been on holiday (includes company paid training)
3 Unemployed and actively seeking work
4 Unemployed, wanting to find a job but not actively looking for one
5 With chronic illness or permanent disability
6 Retired
7 Homemaker, stay-at-home parent, or caregiver

s9_4 Which of the statements below best describes how you feel about your current household income?

1 With our current income we live comfortably

2 With our current income we get by
3 With our current income we have difficulties
4 With our current income we have many difficulties

s10_4 Have you been fired from your primary employment at any time in the past year?

1 Yes
2 No

Currently, to what extent do you feel concerned about...

[PROGRAMMER: ROTATE ORDER RANDOMLY FOR EACH RESPONDENT]

		**Not at all concerned**	**Not too concerned**	**Quite concerned**	**Very concerned**
**s11a_4**	Paying your household bills	0	1	2	3
**s11b_4**	Having to reduce your standard of living	0	1	2	3
**s11c_4**	Having a job	0	1	2	3
**s11d_4**	Paying off loans from the bank or paying mortgage bills	0	1	2	3
